# Bisphenol-A impairs hippocampal neurogenesis by disrupting kinesin-1-dependent mitochondrial trafficking

**DOI:** 10.1016/j.jbc.2026.111375

**Published:** 2026-03-14

**Authors:** Phoolmala, Saurabh Tiwari, Ranjeet Kumar Yadav, Shweta Singh Chauhan, Rajnish Kumar Chaturvedi

**Affiliations:** 1 Molecular Neurotoxicology and Cell Integrity Laboratory, Systems Toxicology and Health Risk Assessment Group, FEST Division, CSIR-Indian Institute of Toxicology Research (CSIR-IITR), Lucknow, Uttar Pradesh (U.P.), India; 2 Academy of Scientific and Innovative Research (AcSIR), Ghaziabad, India; 3 Computational Toxicology Facility, Toxicoinformatics Research Group, CSIR-Indian Institute of Toxicology Research (CSIR-IITR), Lucknow, Uttar Pradesh (U.P.), India

**Keywords:** bisphenol-A, xenoestrogen, neurogenesis, hippocampus, mitochondrial trafficking, kinesin-1 (KIF5A), dynein, SNPH, neurotoxicity

## Abstract

Mitochondrial trafficking ensures proper distribution of mitochondria in energy-demanding neural stem cells (NSC) and neurons for neuronal function and survival. We studied the effects of xenoestrogen bisphenol-A (BPA), found in consumable plastic products, on axonal bi-directional mitochondrial trafficking/movement in neurons. Time-lapse live-cell imaging revealed that BPA exposure impaired anterograde and retrograde axonal mitochondrial trafficking, resulting in altered mitochondrial distribution and density in hippocampal NSC–derived neurons. *In silico* docking studies identified plausible binding of BPA with kinesin-1, dynein, and syntaphilin (SNPH). BPA postnatal exposure reduced mRNA expression and protein levels of mitochondrial trafficking motor proteins kinesin-1 (KIF5A) and dynein, and increased SNPH in the rat hippocampus. BPA significantly reduced colocalization of KIF5A and dynein with TOMM20, Nestin and β-III tubulin *in vitro* and Sox-2 and NeuN *in vivo* and increased SNPH colocalization with TOMM20, Nestin, and Sox-2, indicating impaired mitochondrial trafficking during NSC proliferation and differentiation. Transmission electron microscopy revealed reduced axonal mitochondrial density, synaptic density, increased damaged mitochondria, and synaptic loss following BPA exposure. Pharmacological activation (kinesore; a modulator of kinesin-1 activity) of KIF5A-mediated mitochondrial transport mitigated BPA-mediated impairments in NSC proliferation and neuronal differentiation. BPA-mediated inhibition of mitochondrial distribution, bioenergetics, and synaptic function was reversed by kinesore, as evidenced by increased mitochondrial and synaptic density, increased mitochondrial motility, and reduced damage to synapses and mitochondria, leading to cognitive improvements. These findings implicate the role of kinesin-1 (KIF5A) in reversing BPA-mediated impaired mitochondrial transport, reduced hippocampal neurogenesis, and cognitive deficits in rats.

The brain consumes 20% of the body’s resting total energy, even though it makes up just 2% of its mass (1). Mitochondria, the cell's energy powerhouse, produce ATP, fueling numerous biological processes crucial for neuronal development, including axonal growth and survival, and functions like generating action potentials and facilitating synaptic transmission (2, 3). Neural stem cells (NSCs) consistently multiply and differentiate into neuronal cells by neurogenesis, which depends on ATP, vital for NSC proliferation, differentiation, migration, synaptogenesis, and synaptic plasticity in the hippocampus (4, 5). Disruptions in ATP production or mitochondrial function may hinder new neuron generation, leading to various neurological disorders (6, 7). Consequently, to ensure sufficient ATP levels, neurons have evolved specialized systems called mitochondrial dynamics, including mitochondrial trafficking (8, 9). Mitochondrial dynamics process, comprising mitochondrial biogenesis, fission and fusion, mitochondrial transport, and mitophagy, is essential for maintaining mitochondrial homeostasis (10). Disruption of these processes impairs mitochondrial quality control and bioenergetics, ultimately leading to defective neurogenesis and increased vulnerability to neurodevelopmental and neurodegenerative disorders (8, 11).

Mitochondrial trafficking in neurons refers to the movement of mitochondria within neurons. Long-distance intracellular mitochondrial transport requires ATP hydrolysis and is primarily achieved through microtubules in neurons (12). Kinesin-1 and cytoplasmic dynein are the primary microtubule-associated mitochondrial trafficking motor proteins that transport mitochondria along microtubules (12). Kinesin-1 moves mitochondria anterogradely, primarily toward the axonal terminals, whereas dynein moves them retrogradely, toward the soma/cell body (13). Microtubule organization and polarity in axons are critically important for the precise movement of mitochondria from the soma to axonal and synaptic terminals (14, 15). Kinesin-1 motor proteins, known as KIF5, include three isoforms, including KIF5A, KIF5B, and KIF5C. KIF5A and KIF5C are highly expressed in the brain, whereas KIF5B is expressed in almost all cell types (13, 16). Depending on variations in synaptic activity and bioenergetic state, transition takes place from motile mitochondria (which are only 30% in neurons) to stationary, and from stationary to remobilized (17, 18). Further, syntaphilin (SNPH), a "static anchor," primarily targets the axonal mitochondria's outer membrane and arrests transport by anchoring mitochondria to microtubules (19, 20). Therefore, any defects in mitochondrial trafficking machinery, motor proteins, and mitochondrial delivery can cause blockades of proper distribution and localization of mitochondria in axons, linked to abnormal morphology of cells, synaptic failure, oxidative stress, and axonal degeneration (21, 22, 23, 24, 25). Recent studies suggest that defects in mitochondrial transport/trafficking in axons lead to impaired ATP production and altered calcium buffering at synaptic terminals, causing synaptic dysfunction, neuronal degeneration, and pathogenesis of neurodegenerative diseases (26). A few studies also suggest that environmental chemical exposure causes impairment of mitochondrial trafficking (27, 28).

Bisphenol-A (BPA, 2, 2′-*bis* (4-hydroxyphenyl) propane), an environmental endocrine-disrupting chemical (EDC), is broadly utilized in the production of polycarbonate consumer plastic materials and epoxy resins (29, 30, 31). Several epidemiological reports revealed the presence of BPA metabolites and their chemical traces in blood, breast milk, urine, and pregnant women's serum (32, 33, 34). Humans are exposed to BPA through BPA-containing food, water, and drinking materials. It exerts harmful effects and pathogenesis of brain diseases through crossing of the placental and blood–brain barrier (35, 36, 37). Our previous studies also found BPA-mediated neurotoxicity *via* inhibited hippocampal neurogenesis/oligodendrogenesis and myelination in the rat brain, causing deficiencies in learning and memory (29, 30, 38). We also found reduced proliferation and neuronal differentiation of NSC after BPA exposure in the rat brain *via* the impaired Wnt/β-catenin signaling pathway (31). Similarly, BPA exposure inhibits the ubiquitin proteasomal system by affecting proteasomal activity and thus inhibiting neurogenesis (39).

Our studies also suggested excessive levels of dynamin-related protein 1 (DRP1)–dependent abnormal mitochondrial dynamics, as well as reduced levels of growth factor Erv1-like (GFER) and peroxisome proliferator–activated receptor gamma coactivator-1 alpha–dependent mitochondrial biogenesis, contributing to neurodegeneration and cognitive deficits (40, 41, 42). An additional part of the dynamics is mitochondrial movement beyond fusion and fission (43). However, the effects of BPA exposure on the mechanism of axonal mitochondrial trafficking during neurogenesis in the rat brain have not been studied yet. To address this, we investigated the effects of BPA on axonal mitochondrial trafficking in the rat hippocampus during NSC proliferation and differentiation in the hippocampal-derived NSC culture and the rat brain hippocampus. We found that BPA disrupts axonal mitochondrial movement by modulating motor proteins kinesin-1 (KIF5A) and dynein, altering mitochondrial distribution and density in hippocampal NSC–derived neurons. BPA reduced KIF5A and dynein expression, increased the anchoring protein SNPH levels, and impaired mitochondrial dynamics during NSC proliferation and differentiation. Transmission electron microscopy (TEM) analysis revealed mitochondrial and synaptic loss, linked to cognitive deficits. Kinesin-1 (KIF5A) activation by kinesore reversed BPA-induced mitochondrial and synaptic impairments, highlighting the role of KIF5A in BPA-induced neurotoxicity and reduced hippocampal neurogenesis.

## Results

### BPA-mediated alterations in mitochondrial transport in the rat brain hippocampal NSC–derived neurons

**Figure 1:**
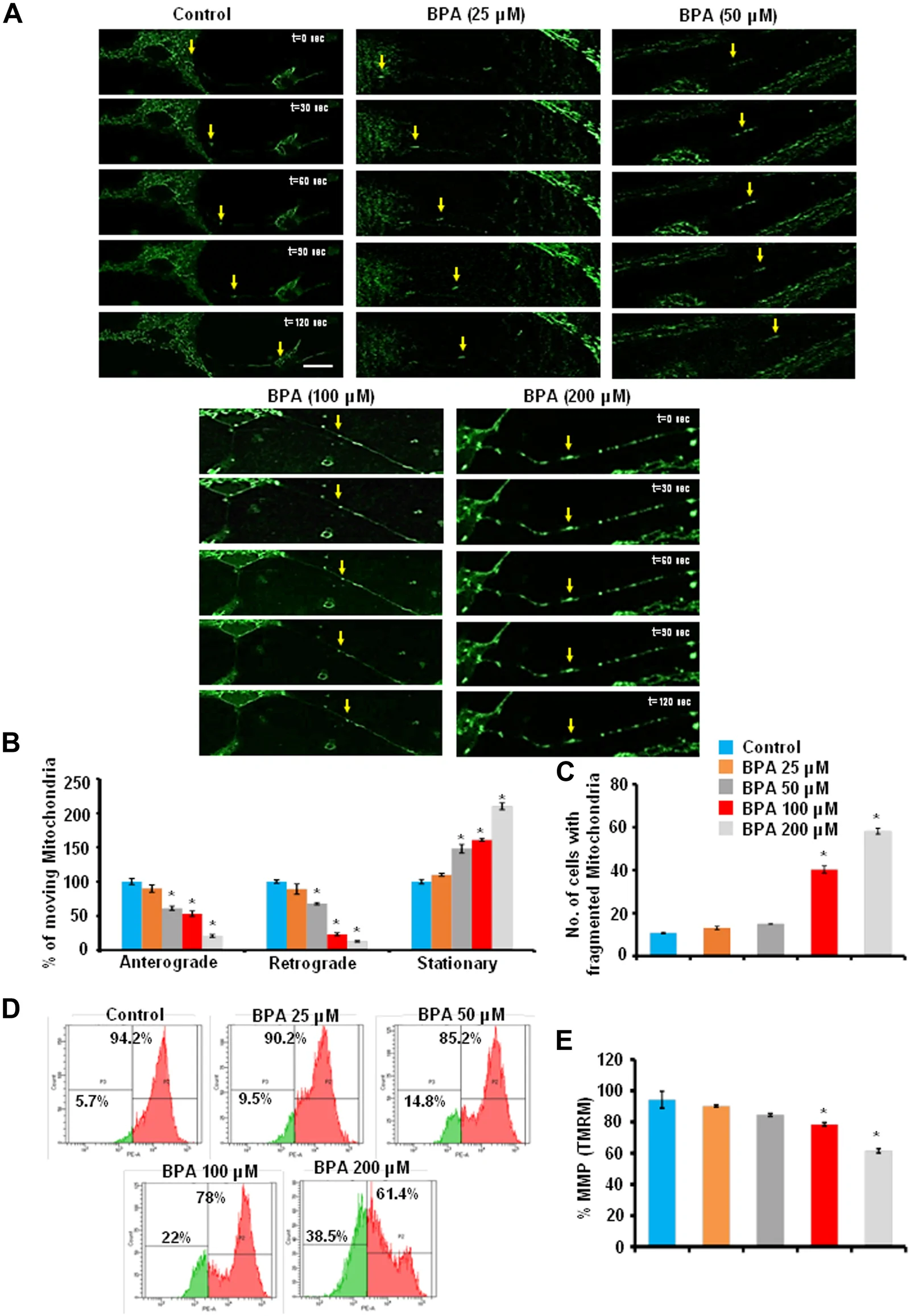
**BPA alters mitochondrial transport in rat hippocampal NSC–derived neurons in the hippocampus.***A* and *B*, effects of different concentrations of BPA (25, 50, 100, and 200 μM) on mitochondrial movement (trafficking) in hippocampal NSC–derived neurons using fluorescence time-lapse live-cell imaging. The movement of fluorescently labeled mitochondria in axons was studied using the mitochondrial fluorescent dye MitoTracker *Green*. BPA exposure caused a significant (∗*p* < 0.05), dose-dependent reduction in anterograde and retrograde mitochondrial movement, with statistically significant effects observed at ≥50 μM, accompanied by an increased proportion of stationary mitochondria. Quantitative analysis was performed in 15 microscopic fields per group. The mean ± SEM (n = 3 individual experiments/group). ∗*p* < 0.05. The scale bar represents 10 μm. *C*, MitoTracker fluorescence imaging revealed a dose-dependent increase in mitochondrial fragmentation, with significant morphological alterations observed at ≥100 μM BPA. *D* and *E*, mitochondrial membrane potential (MMP), assessed by TMRM-based flow cytometry, showed a concentration-dependent reduction in fluorescence intensity, with a significant loss of MMP detected only at ≥100 μM. The data are represented as mean ± SEM (n = 3 individual experiments/group). ∗*p* < 0.05. BPA, bisphenol-A; NSC, neural stem cell; TMRM, tetramethyl rhodamine methyl ester.

Previously, we found that BPA reduces NSC proliferation and differentiation by altering the mitochondrial dynamics process and impairing the ubiquitin-proteasomal system in the hippocampus, ultimately resulting in deficits in learning and memory in rats (39, 40). Since neurogenesis is an energy-driven process, and for normal neurotransmission, mitochondrial movements take place in the anterograde (away from the soma) and retrograde (toward the soma) direction (44). Thus, we evaluated the role of different concentrations (25, 50, 100, and 200 μM) of BPA exposure on mitochondrial movement (trafficking) in the hippocampal NSC–derived neurons using fluorescence time-lapse live-cell imaging by confocal microscope. BPA caused a significant, dose-dependent decrease in axonal mitochondrial anterograde and retrograde movements in NSC-derived neuronal cultures as compared with control cultures, with statistically significant reductions observed at 50 μM and higher BPA concentrations (Fig. 1, *A* and *B*, ∗*p* < 0.05). These results are corroborated by the observation of a significantly increased number of stationary mitochondria in BPA-treated cultures at ≥50 μM concentrations (∗*p* < 0.05). These results imply that BPA alters axonal mitochondrial movement in both the anterograde and retrograde directions and increases the population of static mitochondria in axons in a dose-dependent manner. To determine whether these transport defects reflected a generalized or specific impairment of intracellular organelle trafficking, we additionally examined lysosomal and endosomal movements after BPA exposure. BPA exposure did not produce significant changes in lysosomal or endosomal movement as compared with controls (Fig. S1, *A*–*D*), indicating that BPA-induced trafficking defects may be specific for mitochondria rather than exerting a generalized inhibitory effect on organelle transport.

### BPA increased the number of fragmented mitochondria and reduced mitochondrial membrane potential (MMP) in the rat brain hippocampal NSC–derived neuron cultures

To determine whether BPA exposure affects mitochondrial integrity, we evaluated the effects of different concentrations of BPA exposure on mitochondrial morphology in cultures (40, 45). MitoTracker fluorescence imaging revealed a dose-dependent increase in mitochondrial fragmentation, with significant alterations in mitochondrial morphology observed at 100 μM and above BPA concentrations (Fig. 1*C*, ∗*p* < 0.05). No significant morphological alterations were detected at lower BPA concentrations, indicating that mitochondrial fragmentation occurs predominantly at ≥100 μM and above BPA concentrations. In parallel, MMP was assessed by flow cytometry using tetramethyl rhodamine methyl ester (TMRM) staining in cultures exposed to BPA. A concentration-dependent reduction in TMRM fluorescence intensity was observed, with a significant loss of MMP detected only at an exposure of ≥100 μM BPA relative to control cultures (Fig. 1, *D* and *E*, ∗*p* < 0.05). Together, these results indicate that BPA causes mitochondrial depolarization and fragmentation.

### BPA exposure altered mitochondrial distribution and architecture in the NSC-derived neuron cultures and the rat brain hippocampus

**Figure 2:**
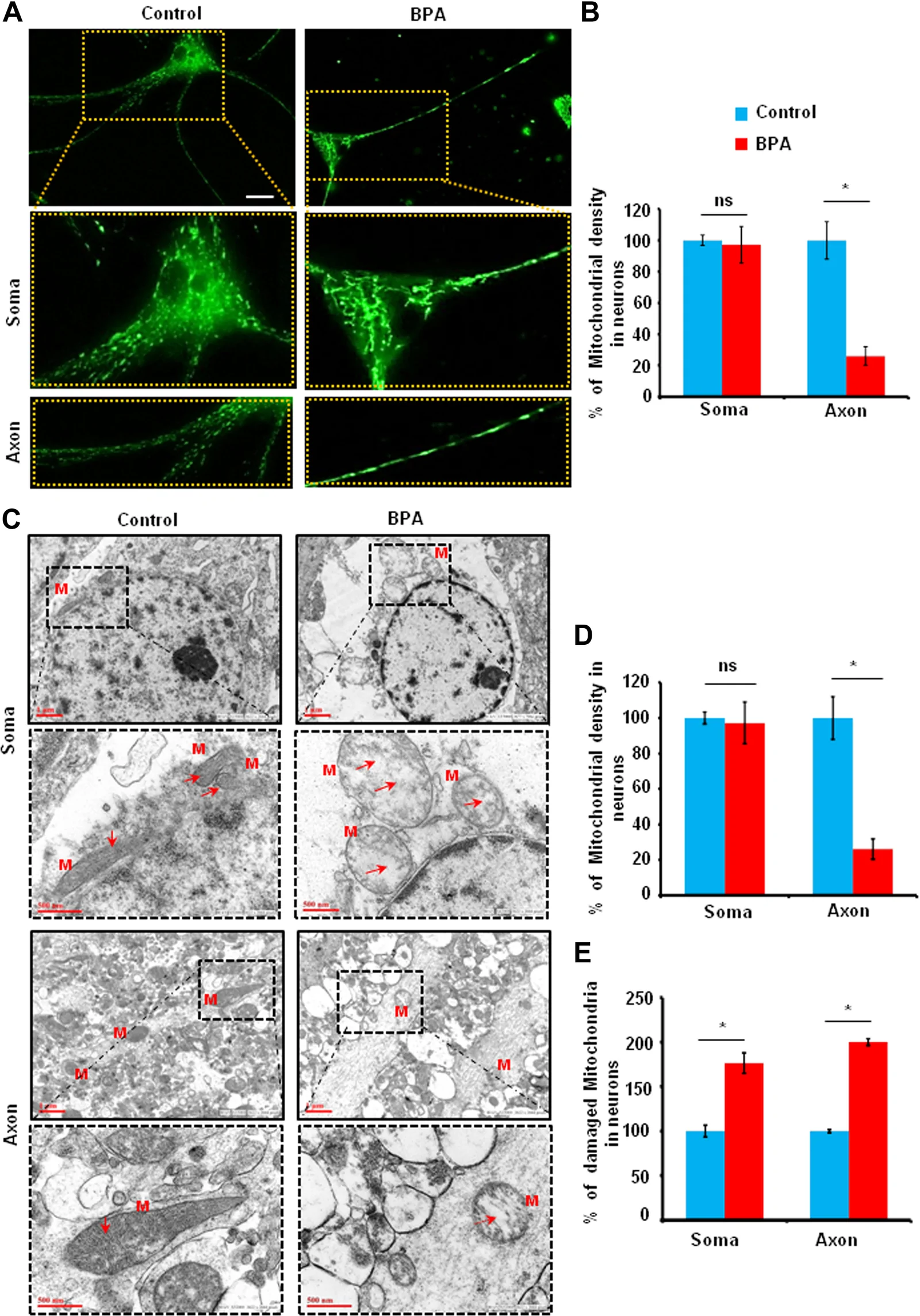
**BPA-mediated impaired mitochondrial density and architecture in the NSC-derived neuron cultures and the hippocampus region of the brain.***A* and *B*, MitoTracker *Green* fluorescence localization *in vitro* suggested BPA (100 μM) treatment significantly (∗*p* < 0.05) reduced mitochondrial density in axons but not in soma. The mean ± SEM (n = 3 individual experiments/group). ∗*p* < 0.05. The scale bar represents 50 μm. *C*–*E*, transmission electron microscopy (TEM)–based ultrastructure analysis *in vivo* suggested that BPA (40 μg/kg body weight) significantly (∗*p* < 0.05) reduced the mitochondrial density in axons but not in soma (the scale bar represents 1 μm). An *inset* showing magnified images of the selected area also revealed an increased number of damaged mitochondria in both axon and soma after BPA exposure as compared with the control (the scale bar represents 500 nm). The data are represented as mean ± SEM (n = 3 rats/group). ∗*p* < 0.05. BPA, bisphenol-A; M, mitochondria; NSC, neural stem cell.

Mitochondria are transported anterogradely by the kinesin-1 (KIF5s) motor protein, and any defects in it decrease the transport of mitochondria along axonal processes (18). Next, we investigated the mitochondrial density in hippocampal NSC–derived neurons following BPA exposure *in vitro* (Fig. 2, *A* and *B*). Through MitoTracker Green fluorescence localization, we observed that BPA (100 μM) exposure significantly (∗*p* < 0.05) reduced mitochondrial density in axons but not in soma (cell body) (Fig. 2, *A* and *B*), suggesting that BPA significantly reduced mitochondrial transport into axons. Furthermore, we studied the effects of BPA exposure on mitochondrial density and architecture in the hippocampal neurons *in vivo*. TEM analysis showed that BPA caused a significant (∗*p* < 0.05) reduction in mitochondrial density only in axons, not in the soma of the rat brain hippocampus neurons at postnatal day (PND) 90, as compared with controls (Fig. 2, *C* and *D*). However, localizing increased damaged mitochondria with abnormal cristae morphology suggested BPA-mediated alterations in mitochondrial trafficking in the axons and soma (Fig. 2, *C* and *E*). Altogether, these findings depict that BPA-mediated alterations in the mitochondrial density and morphology are linked with the reduced mitochondrial trafficking in rat hippocampal neurons.

### Prediction of BPA interaction with mitochondrial trafficking machinery proteins: *In silico* studies

**Figure 3:**
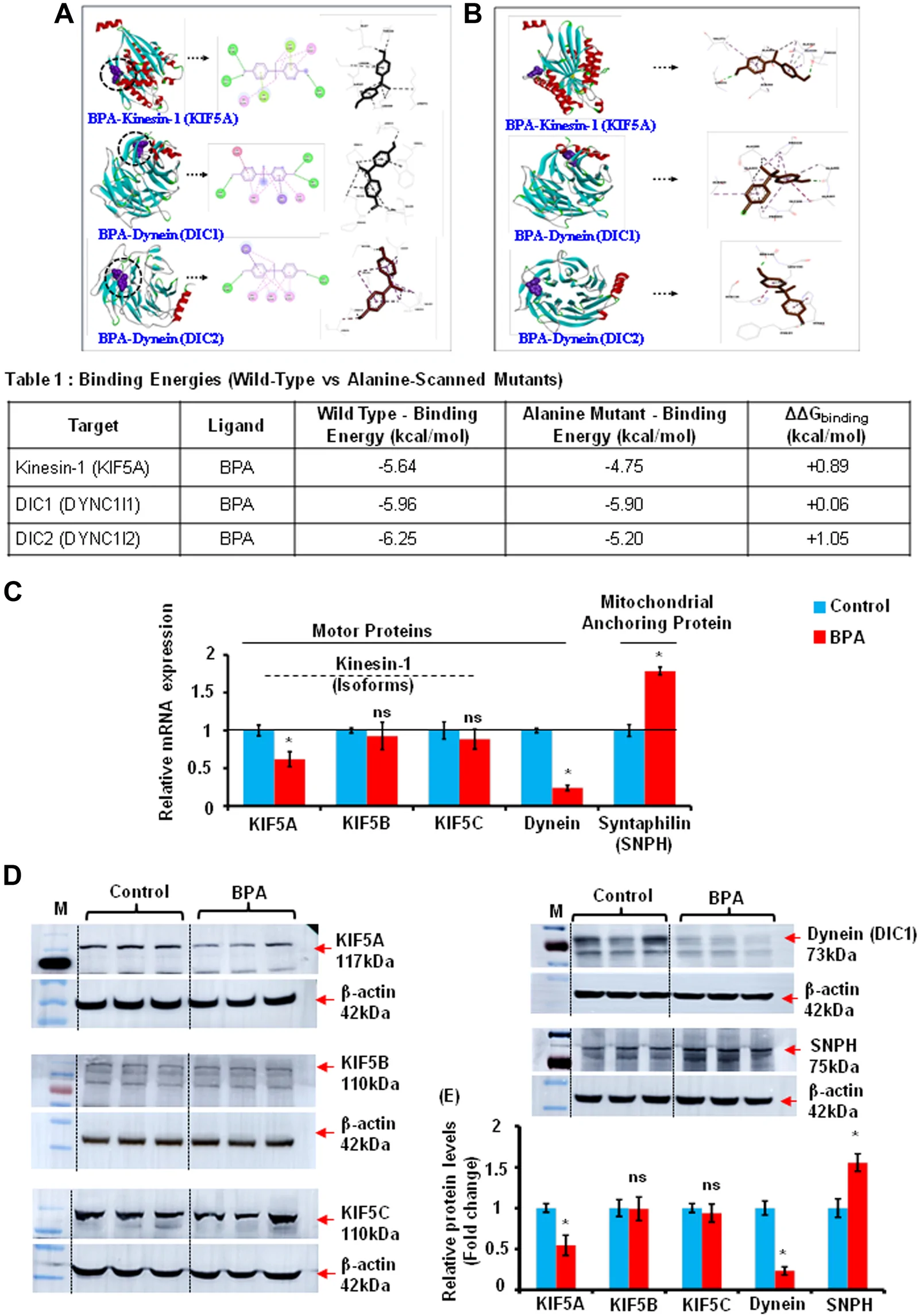
**BPA-mediated alterations in the expression and the levels of mitochondrial trafficking proteins in the hippocampus: *in vivo* and *in silico* prediction studies.***A* and *B*, *in silico* prediction of the interaction of BPA with mitochondrial trafficking machinery proteins. Molecular docking studies of BPA were done within the catalytic site of kinesin-1 (KIF5A), dynein chains (DYNC1/1, DYNC1/2), and syntaphilin (SNPH). The *ribbon diagram* shows the secondary structure of the proteins consisting of coiled-coil α helices and β-sheet strands. Another view is the 2D and 3D surface representation of the binding of ligands to the active site residues of the target. The ligand is displayed in a ball-and-stick model (*maroon-red color*), and amino acid residues of proteins are displayed in an inline format (carbon: *gray*, nitrogen: *blue*, oxygen: *red*, and sulfur: *yellow*). Molecular interactions are represented by *colored dashed lines* as follows: hydrogen bonds: *green*, electrostatic: *orange*, and hydrophobic: *pink*, respectively. *In silico* docking and alanine scanning reveal specific, high-affinity binding of BPA to KIF5A and DYNC1I2, with reduced binding upon alanine mutation. *C, D,* and *E*, effects of BPA treatment (40 μg/kg body weight, oral) during postnatal day (PND) 21–PND90 on the expression/levels of mitochondrial transport–trafficking genes and proteins were studied by quantitative RT–PCR and Western immunoblotting in the hippocampus region of the brain. β-Actin served as a housekeeping control for normalization. BPA exposure significantly (∗*p* < 0.05) reduced mRNA expression and protein levels of KIF5A and dynein (DIC1) and increased SNPH. ∗*p* < 0.05, ns, nonsignificant. The data are represented as mean ± SEM (n = 6 rats/group). ∗*p* < 0.05, ns, nonsignificant. Marker is represented as M. BPA, bisphenol-A.

We found that BPA exposure decreases axonal bidirectional mitochondrial trafficking in the hippocampal NSC–derived neuronal cultures. Several motor proteins are involved in the regulation of axonal mitochondrial trafficking. Thus, next, we performed a molecular docking study to understand the significant interaction/binding of BPA with motor proteins of mitochondrial trafficking machinery using AutoDock Studio (46). We studied the plausible binding of BPA with kinesin-1 isoforms (KIF5A, KIF5B, and KIF5C), dynein heavy chain (DYNH), dynein intermediate chain (DYNC1I1 and DYNC1I2), dynein light chain (DYLL1), and SNPH. BPA interacts and binds with kinesin-1 isoform KIF5A with active participation of specific residues (Thr330, Glu7, Ala270, Leu269, Lys328, Ile327, and Lys274) (Fig. 3*A*). Three hydrogen bonds (H-bonds) are formed between BPA and KIF5A. Three H-bonds between H32, H33, and O1 of BPA with OE1 atoms of Glu7, O atoms of Ala270, and OG1 atoms of Thr330, respectively; two Pi-donor H-bonds between BPA with O atoms of Lys328 and Leu269, and six hydrophobic interactions, in which C6 of BPA with Leu269, Ile327 of KIF5A, C7 of BPA with both Leu279, Lys274 of KIF5A and two unknown atoms of BPA with Leu269 and Lys328. BPA strongly binds to the DYNC1I1 (DIC1) and DYNC1I2 (DIC2). Active participation of specific residues (Val496, Asn615, Pro542, Val543, Trp618, Val543, Pro613, and Ala495) takes place in case of DYNC1I1–BPA interaction (Fig. 3*A*). Total three H-bonds and seven hydrophobic interactions are generated: two H-bonds between O2, H32, and H33 of BPA with O atoms of Asn615, Pro542 and N atoms of Val496; two hydrophobic interactions between C6 of BPA with Val543, Pro613 and last, five hydrophobic interactions among unknown atoms of BPA with CG1 of Val543, Trp618, Pro613, and Ala495. In case of DYNC1I2–BPA interaction, active participation of specific residues (Met388, Phe314, Ile331, Val361, Leu372, and Val323) takes place (Fig. 3*A*). There are total, two H-bonds and nine hydrophobic interactions are formed: two H-bonds between H32 and H33 of BPA with O atoms of Met388, Phe314; two hydrophobic interactions between C6 of BPA with Ile331 and Val361, two hydrophobic interactions between C7 of BPA with Val361 and Leu372, and last, five hydrophobic interactions among unknown atoms of BPA with CB of Ile331, Val323, and Leu372. The docking scores (binding energy) for the interaction of BPA with kinesin-1 (KIF5A), dynein (DYNC1I1 and DYNC1I2) are −5.64 kcal/mol, −5.96 kcal/mol, and −6.25 kcal/mol, respectively (Table 1). Interestingly, we found BPA showed weak binding affinity toward KIF5B, KIF5C, and SNPH as indicated by a poor docking score (Fig. S2*A* and Table S1). Overall molecular docking results exhibited strong binding affinities toward the motor proteins kinesin-1 (KIF5A) and dynein, whereas showing weaker interaction with kinesin-1 (KIF5B, KIF5C) and SNPH, suggesting that BPA may preferentially interfere with mitochondrial transport through modulation of KIF5A.

**Table 1:** Binding energies (wildtype *versus* alanine-scanned mutants)

Target	Ligand	Wildtype-binding energy (kcal/mol)	Alanine mutant–binding energy (kcal/mol)	Δ ΔG_binding_ (kcal/mol)
Kinesin-1 (KIF5A)	BPA	−5.64	−4.75	+0.89
Dynein (DIC1) (DYNC1I1)	BPA	−5.96	−5.90	+0.06
Dynein (DIC2) (DYNC1I2)	BPA	−6.25	−5.20	+1.05

To strengthen the docking analysis and mechanistic interpretation of BPA binding to motor proteins, *in silico* alanine-scanning mutagenesis (47) and subsequent AutoDock-based redocking (41) were carried out for KIF5A, DYNC1I1, and DYNC1I2. Alanine mutations reduced BPA binding affinity in KIF5A and DYNC1I2 (Fig. 3*B* and Table 1). KIF5A and DYNC1I2 displayed loss of affinity upon mutation (ΔΔG_binding_ = +0.89 kcal/mol and +1.05 kcal/mol, respectively), supporting a structured and cooperative binding mode and indicating that BPA recognition relies on specific hydrophobic and aromatic residues (Fig. 3*B*, Table 1). The combined docking and alanine scanning results clearly indicate that BPA may form specific interactions with KIF5A and DYNC1I2. Residue-level energetic contributions confirm that BPA binding to KIF5A and DYNC1I2 depends on defined hotspots, supporting specific molecular recognition rather than a nonspecific association. These *in silico* findings were further supported by *in vitro* immunocytochemical BPA-tagged quantum carbon dot co-localization studies (48). BPA-tagged quantum carbon dots localized in the cells, as evident from colocalization with mitochondrial protein KIF5A (Fig. S2*B*). These results also suggest a plausible interaction of BPA with KIF5A, confirming a probable biochemical association at the protein level.

### BPA alters the mitochondrial trafficking factors in the rat brain hippocampus

*In silico* studies gave a preliminary idea about the plausible binding of BPA with mitochondrial motor proteins. Thus, we validated these findings at the molecular level and examined the effects of BPA on gene expression and protein levels of mitochondrial trafficking regulatory machinery in the rat brain hippocampus at PND90. We performed quantitative RT–PCR (qRT–PCR) and Western blotting analysis of kinesin-1 isoforms (KIF5A, KIF5B, and KIF5C), dynein, and SNPH. Kinesin-1 and dynein are the motor proteins for mitochondrial trafficking in the axon, which are essential for bidirectional transport of mitochondria, respectively (49). SNPH is a static mitochondrial anchoring protein that also predominantly regulates mitochondrial trafficking (50). We observed significantly (∗*p* < 0.05) decreased gene expression of kinesin-1 isoform KIF5A (neuronal and mitochondrial specific) as well as dynein, in the BPA-exposed rats as compared with controls (Fig. 3*C*). BPA treatment led to a significant upregulation of SNPH mRNA expression as compared with the control group, whereas no significant changes were observed in the expression of KIF5B and KIF5C (Fig. 3*C*). Interestingly, no significant changes in the expression of alternate motor and adaptor protein genes (Syntabulin, RanBP2, OGT, Myosin5a, and Myosin5b) and microtubule-associated protein (MAP1B) were observed (Fig. S3).

BPA significantly (∗*p* < 0.05) reduced KIF5A and dynein protein levels and increased SNPH protein levels, whereas no significant changes in KIF5B and KIF5C protein levels (Figs. 3, *D*, *E* and S4, *A*–*E*). Collectively, these findings suggest that BPA treatment causes a significant reduction in both gene expression and protein levels of key components involved in mitochondrial trafficking in the rat hippocampus. The data further suggest that BPA affects motor proteins and mitochondrial anchoring proteins, while having no significant impact on alternate motor proteins or microtubule-associated proteins in the rat brain hippocampus (Fig. 3, *C*–*E*)

### BPA-mediated alterations in mitochondrial trafficking regulatory factors during NSC proliferation and hippocampal NSC–derived neuronal differentiation *in vitro*

**Figure 4:**
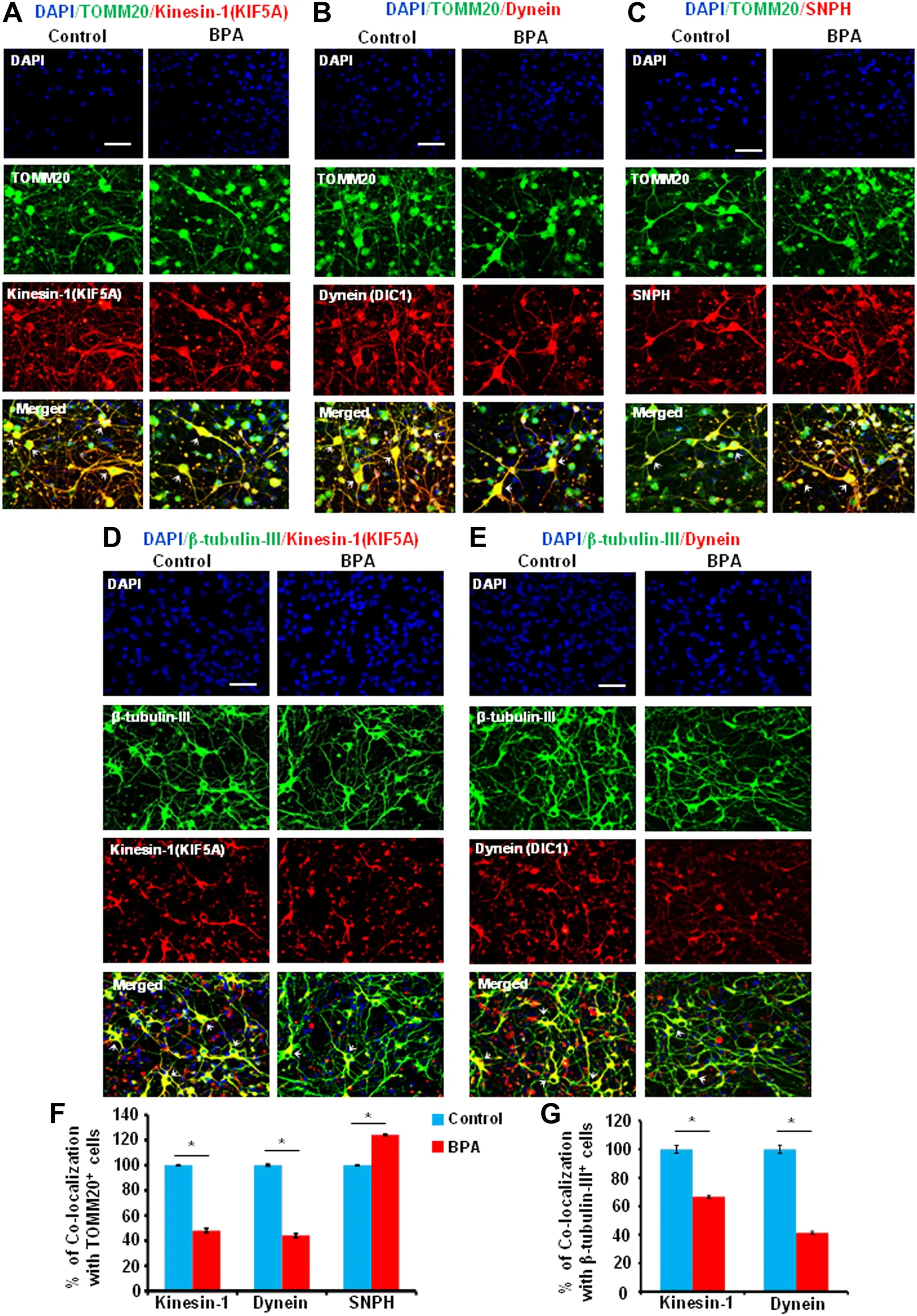
**BPA reduced mitochondrial transport during neuronal differentiation in the rat hippocampal NSC–derived neuronal cultures.***A*–*G*, the representative images of immunofluorescence with the graphs related to quantitative analysis suggested BPA (100 μM) significantly decreased KIF5A and dynein colocalization with TOMM20 and β-tubulin-III. BPA significantly (∗*p* < 0.05) increased SNPH–TOMM20 colocalization in the NSC culture from the rat brain hippocampus. DAPI was used as a nuclear stain. The data are represented as mean ± SEM (n = 3 individual experiments/group). ∗*p* < 0.05. The scale bar represents 50 μm. BPA, bisphenol-A; DAPI, 4,6-diamidino-2-phenylindole; NSC, neural stem cell; SNPH, syntaphilin.

We next examined the effects of BPA on the immunocytochemical localization and protein levels of mitochondrial trafficking motor machinery by double immunocolabeling of KIF5A, dynein, and SNPH with Nestin (neural progenitor marker) in the NSC cultures. Immunofluorescence analysis exhibited that BPA exposure significantly (∗*p* < 0.05) reduced the colocalization of KIF5A–Nestin^+^, dynein–Nestin^+^, and enhanced SNPH–Nestin^+^ colabeled cells as compared with controls (Fig. S5, *A*–*D*). Next, we examined the effects of BPA on mitochondrial trafficking–associated proteins in the mitochondria of NSC-derived neurons by coimmunolabeling of KIF5A, dynein, and SNPH with the mitochondrial marker TOMM20. BPA treatment resulted in a significant (∗*p* < 0.05) reduction in KIF5A–TOMM20^+^ and dynein–TOMM20^+^ colocalization, accompanied by a marked increase in SNPH–TOMM20^+^ colocalization as compared with controls (Fig. 4, *A*–*C* and *F*). To further evaluate mitochondrial trafficking during neuronal differentiation, we studied double immunocolocalization of KIF5A and dynein with βIII-tubulin (a mature neuronal marker). BPA-treated neurons showed a significant (∗*p* < 0.05) decrease in colocalization intensity of both KIF5A–βIII-tubulin^+^ and Dynein–βIII-tubulin^+^ as compared with the control group (Fig. 4, *D*, *E* and *G*). Together, these findings suggest that BPA exposure altered the localization of mitochondrial trafficking proteins, resulting in impaired axonal mitochondrial trafficking activity during both NSC proliferation and neuronal fate determination *in vitro*.

### BPA-mediated alterations in mitochondrial trafficking regulatory factors during NSC proliferation and neuronal fate determination in the rat brain hippocampus

**Figure 5:**
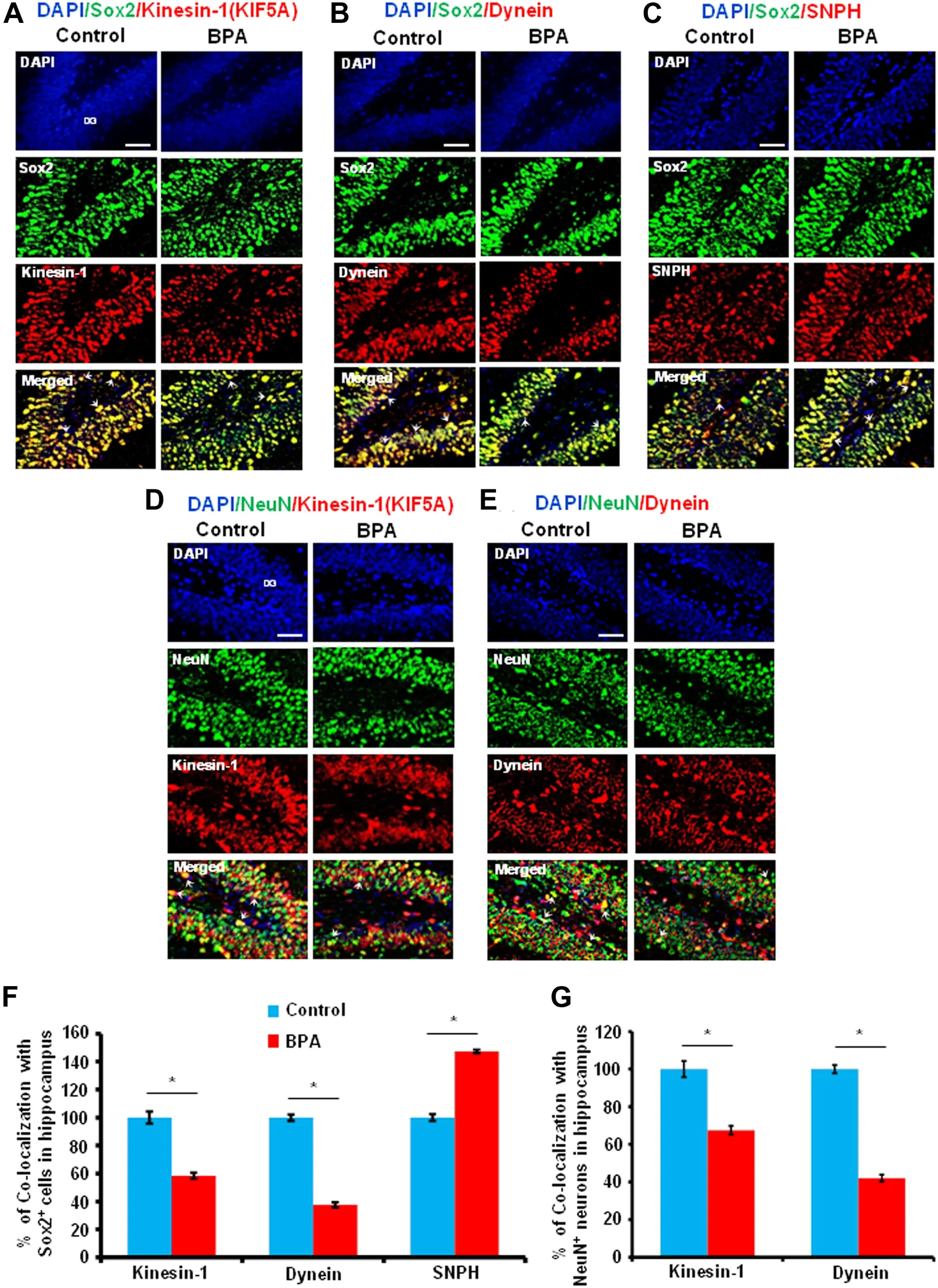
**Chronic exposure to BPA reduced mitochondrial transport during NSC proliferation and neuronal differentiation in the rat brain hippocampus.***A*–*G*, the representative images of immunofluorescence with the graphs related to quantitative analysis suggested BPA (40 μg/kg body weight) significantly (∗*p* < 0.05) decreased KIF5A and dynein colocalization with Sox-2^+^ NSCs and NeuN^+^ neuronal cells, whereas it significantly increased SNPH and Sox-2^+^ cells in NSCs in the hippocampus region of the rat brain. The data are represented as mean ± SEM (n = 5 rats/group, five sections/rat). ∗*p* < 0.05. The scale bar represents 50 μm. BPA, bisphenol-A; NSC, neural stem cell.

To further corroborate the results of our *in vitro* studies, we next examined the effects of BPA on mitochondrial trafficking regulatory factors during NSC proliferation by studying colabeling of KIF5A, dynein, and SNPH with Sox-2 (NSC marker). BPA significantly (∗*p* < 0.05) reduced the colocalization of the KIF5A–Sox-2^+^, dynein–Sox-2^+^, and increased SNPH–Sox-2^+^ colabeled cells in the BPA-treated group as compared with the control (Fig. 5, *A*–*C* and *F*). Furthermore, to determine the effect of BPA on mitochondrial trafficking of specific motor proteins during neuronal differentiation, we performed immunocolocalization studies of KIF5A and dynein with the mature neuronal marker neuronal nuclei (NeuN). BPA significantly (∗*p* < 0.05) reduced the colocalization intensity of the KIF5A–NeuN^+^ and dynein–NeuN^+^ (Fig. 5, *D*, *E* and *G*). Overall, these results suggest that BPA exposure altered axonal mitochondrial trafficking activity during NSC proliferation and neuronal fate determination in the hippocampus.

### BPA-mediated impaired mitochondrial trafficking resulted in synaptic loss in the NSC-derived neuron cultures and the rat brain hippocampus

**Figure 6:**
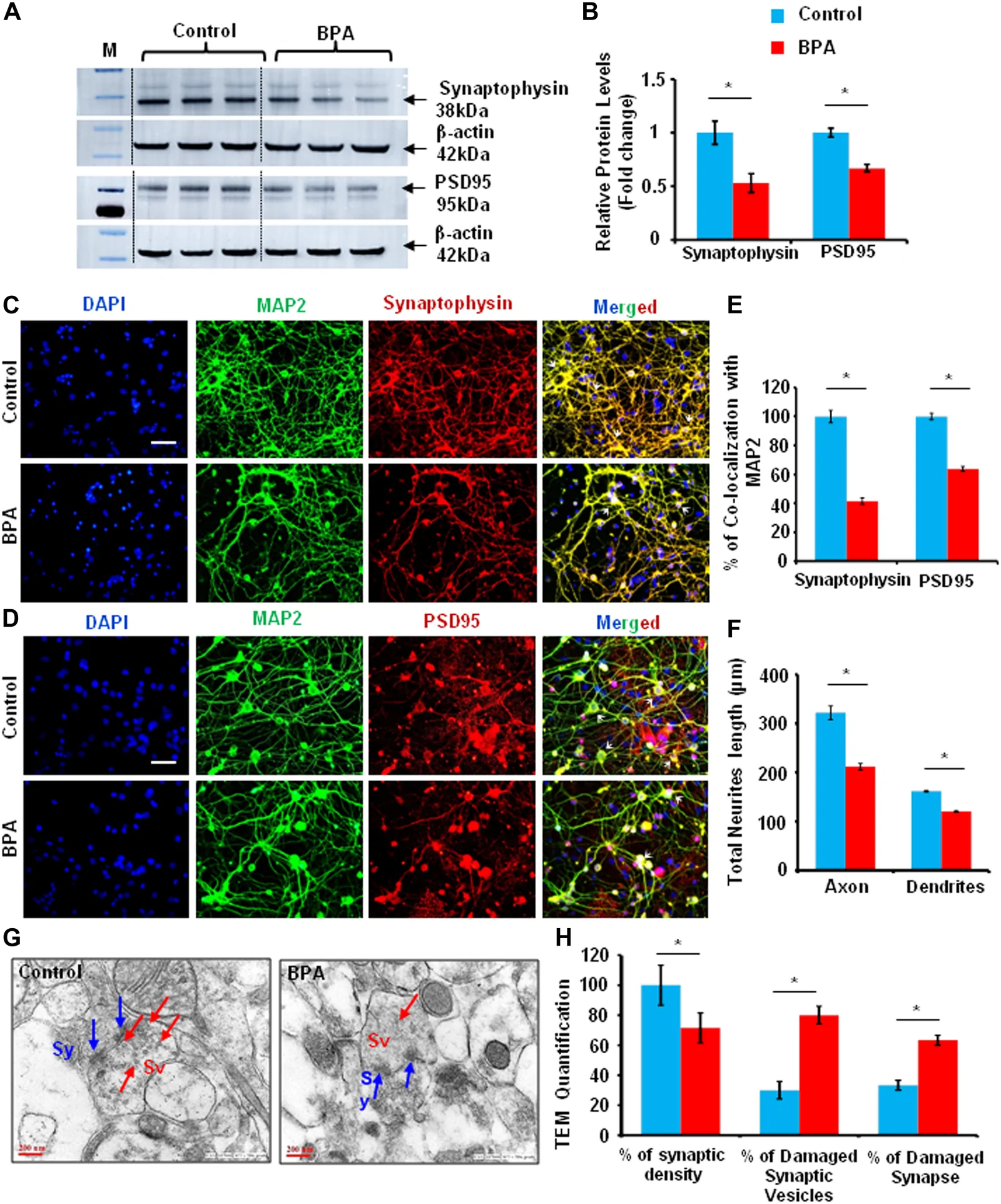
**BPA-mediated impaired mitochondrial trafficking disrupts synaptic integrity, neuronal morphogenesis, and cognitive functions in rats.***A* and *B*, Western blot analysis for synaptic function–related proteins (synaptophysin and PSD95) in the BPA (40 μg/kg body weight)-treated and control rats at PND90 in the hippocampus. BPA significantly (∗*p* < 0.05) decreased synaptic marker synaptophysin and PSD95 protein levels. The quantitative relative protein density was normalized with β-actin. The data are represented as mean ± SEM (n = 6 rats/group). ∗*p* < 0.05. Marker is represented as M. *C*–*F*, the quantitative analysis of the representative immunofluorescence images suggested decreased colocalization of synaptophysin and PSD95 with MAP2 (mature neuron marker) in the BPA-treated group (100 μM) in NSC-derived neuronal cells. BPA treatment significantly decreased the length of dendrites and neurites in differentiated neuronal cells. The data are represented as mean ± SEM. The data are represented as mean ± SEM (n = 3 individual experiments/group). ∗*p* < 0.05. The scale bar represents 50 μm. *G* and *H*, TEM analysis was performed to confirm the effects of BPA-mediated altered synaptic protein levels on synapse morphology, synaptic density, and synaptic vesicle integrity. BPA exposure significantly (∗*p* < 0.05) reduced synaptic density, increased the number of damaged synaptic vesicles with damaged membranes, and elevated the number of structurally damaged synapses as compared with the control. The data are represented as mean ± SEM (n = 3 rats/group). ∗*p* < 0.05. The scale bar represents 200 nm. BPA, bisphenol-A; PND, postnatal day; Sv, synaptic vesicle; Sy, synapse; TEM, transmission electron microscopy.

Disrupted mitochondrial trafficking and loss of mitochondrial cristae alter mitochondrial distribution and function in neurons, reducing energy production for ATP-dependent processes at synaptic terminals and ultimately leading to synaptic dysfunction (51, 52, 53). Synaptic vesicle precursors are transported anterogradely along axons by kinesin-1 motor proteins, and any disruption in kinesin-1-mediated trafficking impairs their delivery, resulting in reduced synaptic density (54, 55). To assess the impact of BPA on synaptic vesicle trafficking and synaptic integrity in the hippocampus, we analyzed the levels of synaptic proteins, including PSD95 (a postsynaptic marker) and synaptophysin (a presynaptic marker) (Fig. 6, *A* and *B*). Immunoblot analysis revealed that BPA exposure significantly (*p* < 0.05) decreased the levels of PSD95 and synaptophysin in the rat hippocampus as compared with controls (Figs.6, *A*, *B* and S6, *A* and *B*). Next, we studied the effects of BPA exposure on synaptophysin and PSD95 coimmunolocalization with MAP2 (neuronal marker). We found that BPA exposure significantly (∗*p* < 0.05) reduced the colocalization of synaptophysin and PSD95 with MAP2 in neuronal cultures (Fig. 6, *C*–*E*). These results suggest that BPA exposure inhibits the levels of synaptic proteins, which are required for synaptic functions and neuronal transmission. Proper neuronal morphogenesis, including the growth and branching of dendrites and axons, requires efficient mitochondrial transport to meet the energy requirements of early neurites (56). Any disruption of mitochondrial trafficking causes impaired neuronal maturation and connectivity (56, 57). Next, we examined the effects of BPA on neuronal morphogenesis *in vitro*. BPA exposure also altered the morphology of neurons and reduced the number of dendritic and axonal fibers in neuronal cultures (∗*p* < 0.05; Fig. 6, *C*, *D* and *F*). On the other hand, the consequences of altered synaptic protein levels on synapse morphology, synaptic density, and synaptic vesicles were also observed through TEM analysis. BPA exposure significantly (∗*p* < 0.05) decreased synaptic density, increased vesicles with damaged membrane morphology, and increased the number of damaged synapses without forming the active zone as compared with control (Fig. 6, *G* and *H*). These findings indicate that BPA-induced neurotoxicity is associated with impaired synaptic functions in the hippocampus.

### Kinesin-1 (KIF5A) activation/modulation by kinesore restores BPA-mediated impaired mitochondrial trafficking during NSC proliferation and neuronal fate determination in hippocampal NSC–derived cultures

**Figure 7:**
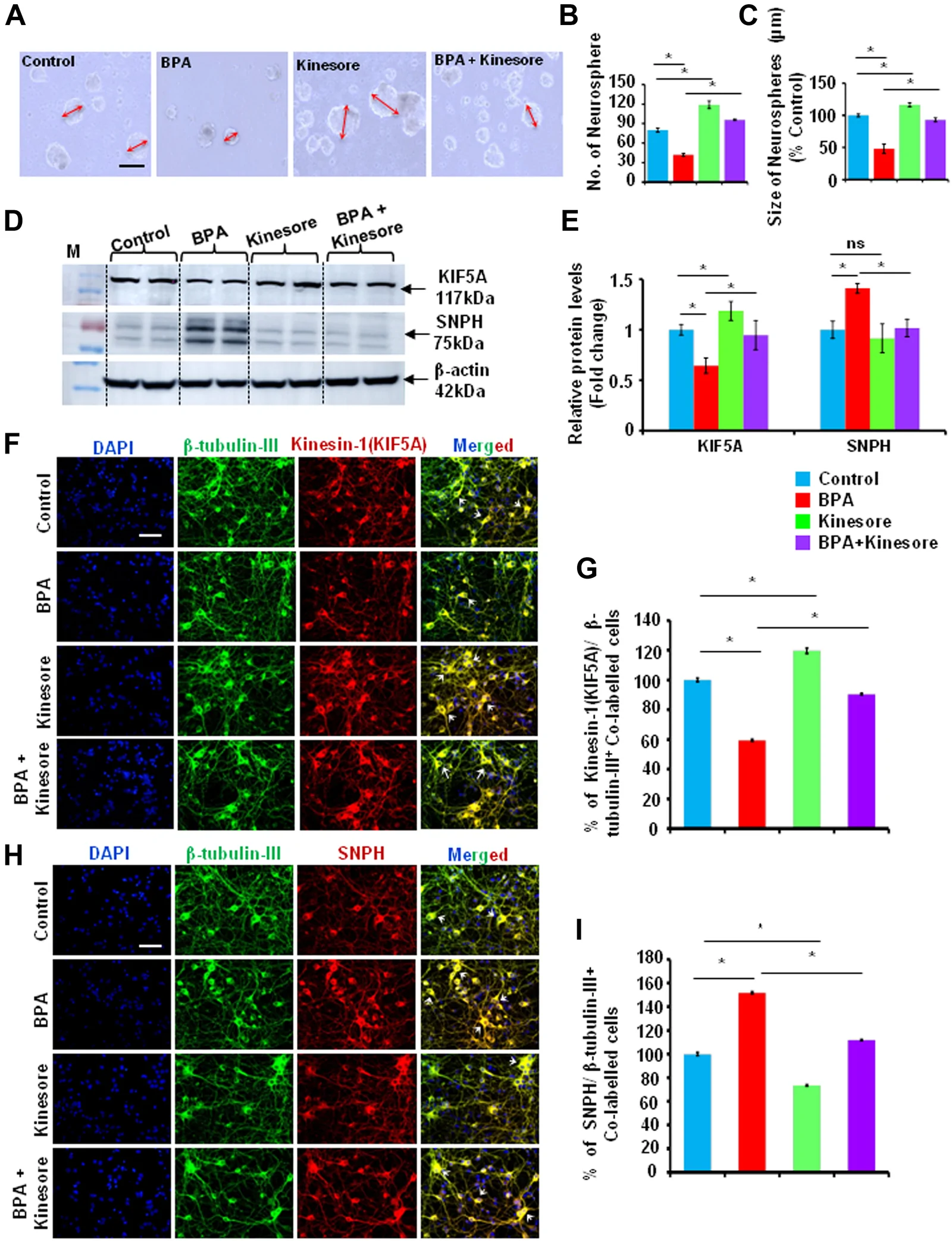
**Activation of kinesin-1 (KIF5A) by kinesore reverses BPA-induced impairments in mitochondrial transport during the proliferation and differentiation of hippocampal NSC–derived cultures.***A*–*C*, NSC cultures were treated with BPA (100 μM) and the KIF5A activator kinesore (50 μM) for 24 h. Neurosphere growth kinetics exhibited a significant decrease in the size and number of neurospheres in the BPA-treated group as compared with the control, whereas there was a significant increase in the kinesore + BPA group as compared with the BPA group. The data are represented as mean ± SEM (n = 3 individual experiments/group). ∗*p* < 0.05. The scale bar represents 100 μm. *D* and *E*, activation of KIF5A by kinesore in BPA-exposed cultures effectively restored the protein levels of both KIF5A and SNPH, compared with cultures treated with BPA alone. β-actin was used as a normalization marker to assess the relative protein density. Marker is represented as M. The data are represented as mean ± SEM (n = 3 individual experiments/group). ∗*p* < 0.05. *F*–*I*, the representative immunofluorescent images and quantitative analysis suggest that KIF5A activation mitigates BPA-mediated alteration in KIF5A and SNPH levels colocalized with β-tubulin III in the hippocampal NSC–derived neuronal culture. DAPI was used as a nuclear stain. The data are represented as mean ± SEM (n = 3 individual experiments/group). ∗*p* < 0.05. The scale bar represents 50 μm. BPA, bisphenol-A; DAPI, 4,6-diamidino-2-phenylindole; NSC, neural stem cell; SNPH, syntaphilin.

Kinesore, a small-molecule activator/modulator of kinesin-1 motor proteins, has recently emerged as a neuroprotective agent, which enhances kinesin-1–driven transport and remodeling of the cytoskeleton (58). Furthermore, to study the specific role of KIF5A in BPA-mediated impairment of mitochondrial trafficking, we carried out pharmacological activation of KIF5A by kinesore in NSC-derived neuronal cultures. We studied whether KIF5A activation could enhance KIF5A-mediated mitochondrial transport, thereby promoting efficient trafficking to regions of high metabolic demand and supporting the proliferation and neuronal fate determination of NSCs. To test this, NSCs were treated with kinesore in the presence and absence of BPA, and proliferation was assessed *via* neurosphere growth kinetics. BPA treatment significantly decreased both the number and size of neurospheres as compared with the control cultures (∗*p* < 0.05; Fig. 7*A*–*C*). Activation of KIF5A led to a significant increase in both the number and relative size of neurospheres in the kinesore-treated group as compared with the control (∗*p* < 0.05; Fig. 7*A*–*C*). Interestingly, KIF5A activation by kinesore restored the BPA-mediated impaired NSC proliferation, as evident from increased neurosphere number and size as compared with BPA-treated cultures (∗*p* < 0.05; Fig. 7*A*–*C*). These findings suggest that KIF5A enhances NSC proliferative potential, as evidenced by increased neurosphere formation and growth, likely through the facilitation of KIF5A-dependent mitochondrial transport.

Our previous findings indicated that BPA-induced downregulation of KIF5A in the rat hippocampus is associated with a concomitant increase in SNPH expression (Fig. 3). BPA exposure resulted in a significant downregulation of KIF5A protein levels, accompanied by an increase in SNPH levels (Fig. 7*D* and *E*). KIF5A activation in BPA-exposed cultures effectively restored KIF5A and SNPH protein levels as compared with BPA alone–treated cultures (Fig. 7*D*, *E* and S7, *A* and *B*). In line with the immunoblot results, kinesore treatment led to a marked increase in KIF5A and a significant decrease in SNPH expression in β III-tubulin–positive neurons in the BPA-treated groups (∗*p* < 0.05; Fig. 7*F*–*I*). Overall, KIF5A activation/modulation restores mitochondrial transport machinery, which may suppress SNPH expression, thereby mitigating BPA-induced disruptions in mitochondrial dynamics.

### **Kinesin-1(KIF5A)-dependent processes are associated with BPA-induced alterations in mitochondrial trafficking, density, and fragmentation in hippocampal NSC-derived neuronal cultures**

**Figure 8:**
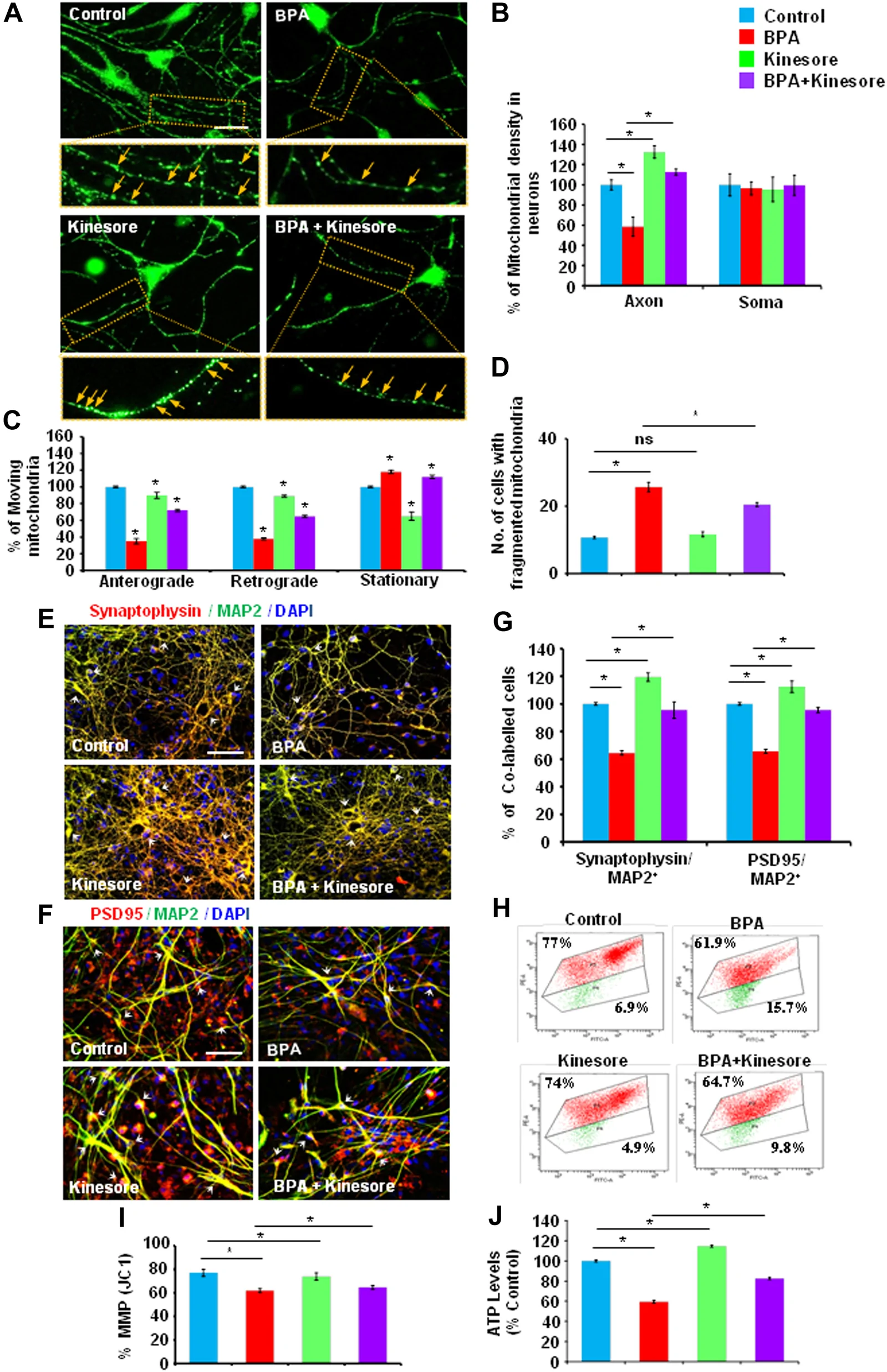
**Kinesin-1 (KIF5A) activation/modulationrescues BPA-mediated impairments in mitochondrial movement, fragmentation, and synaptic dysfunction in hippocampal NSC–derived neurons.** Hippocampal NSCs were treated with BPA (100 μM), and KIF5A activator/modulator kinesore (50 μM) for 24 h. *A*–*C*, MitoTracker Green fluorescence localization suggested that BPA-mediated impaired mitochondrial density and movements were significantly recovered by KIF5A activator kinesore in axons. *Arrows* show mitochondria. The data are represented as mean ± SEM (n = 3 individual experiments/group). ∗*p* < 0.05. The scale bar represents 50 μm. *D*, quantitative analysis suggests that BPA exposure increased mitochondrial fragmentation and kinesore significantly attenuated BPA-induced mitochondrial fragmentation. *E*–*G*, double immunofluorescence staining of synaptophysin and PSD95 with MAP2 in NSC-derived neurons. Cotreatment with kinesore and BPA significantly restored the colocalization of synaptic proteins in neurons (*p* < 0.05), indicating a protective role of KIF5A in maintaining synaptic integrity. DAPI was used as a nuclear stain. The data are represented as mean ± SEM (n = 3 individual experiments/group). ∗*p* < 0.05. The scale bar represents 50 μm. *H*–*J*, MMP was studied through JC-1 by flow cytometry. BPA exposure significantly reduced mitochondrial membrane potential and ATP levels, whereas kinesore significantly restored these effects. The data are represented as mean ± SEM (n = 3 individual experiments/group). ∗*p* < 0.05. BPA, bisphenol-A; DAPI, 4,6-diamidino-2-phenylindole; MMP, mitochondrial membrane potential; NSC, neural stem cell.

To further study the regulatory effects of KIF5A on mitochondrial density, we performed a quantitative analysis of mitochondrial distribution and movement in NSC-derived hippocampal neuron cultures in the presence and absence of BPA and kinesore (KIF5A activator/modulator) (Fig. 8*A*–*C*). BPA treatment resulted in a significant reduction in mitochondrial density and anterograde and retrograde transport within axons (∗*p* < 0.05), with no significant change in the soma as compared with the control cultures (Fig. 8*A*–*C*). Kinesore treatment enhanced axonal mitochondrial density and movement without affecting soma (Fig. 8*A*–*C*). Cotreatment with kinesore and BPA significantly (∗*p* < 0.05) restored mitochondrial density and movement in axons as compared with BPA-treated cultures (Fig. 8*A*–*C* and S8). These findings suggest that KIF5A activation mitigates the BPA-mediated impairments in mitochondrial density and transport to axonal compartments. Impairments in this transport machinery, KIF5A, can lead to reduced mitochondrial density at distal neuronal compartments. In parallel, mitochondrial morphology analysis revealed that BPA increased mitochondrial fragmentation, an effect that was further enhanced by KIF5A inhibition. Activation/modulation of KIF5A by kinesore attenuated BPA-induced mitochondrial fragmentation (∗*p* < 0.05), indicating preservation of mitochondrial integrity (Fig. 8*D*). Together, these findings demonstrate that BPA-mediated impaired KIF5A activity is a critical determinant of mitochondrial transport, axonal mitochondrial density, and structural integrity.

### Kinesin-1 (KIF5A) regulates BPA-mediated alterations in synaptic function in the hippocampal NSC–derived neuronal cultures

KIF5A predominantly plays a vital role in preserving synaptic function and integrity by transporting essential synaptic vesicle precursors within neurons (59, 60). Impairment in KIF5A-driven transport can result in the mislocalization of synaptic components, ultimately disrupting effective neurotransmission (54, 55). Accumulated evidence indicates that KIF5A plays a pivotal role in regulating synaptic function and maintaining synaptic integrity. Disruption in mitochondrial transport alters their density and localization at synapses, compromising the energy supply and calcium balance required for proper synaptic function (61). Therefore, we further studied the role of KIF5A in BPA-mediated impaired synaptic function. To assess the impact of KIF5A activation (kinesore) on synaptic function and integrity, we examined the expression of key synaptic marker proteins, synaptophysin and PSD95, in NSC-derived neuronal cultures using double immunofluorescence labeling with the neuronal marker MAP2. BPA exposure caused reduced colocalization of synaptophysin and PSD95 with MAP2-positive neurons as compared with controls (∗*p* < 0.05; Fig. 8*E*–*G*), indicating compromised synaptic protein distribution and reduced synaptic density. Kinesore-alone treatment significantly increased synaptophysin–MAP2 and PSD95–MAP2 colocalization as compared with control cultures. Kinesore + BPA cotreatment markedly restored the colocalization of both synaptophysin and PSD95 with MAP2 in neurons (∗*p* < 0.05), suggesting a protective role of KIF5A modulation in preserving synaptic structure, potentially through modulation of mitochondrial transport and energy supply. Overall, these findings suggested that disruption in KIF5A function is linked to impaired trafficking of both mitochondria and synaptic vesicles, leading to synaptic dysfunction and degeneration (Fig. 8*E*–*G*). The involvement of Kinesin-1(KIF5A)-dependent processes in maintaining synaptic function is supported by independent functional observations of increased synaptic markers by kinesore.

### Kinesin-1 (KIF5A) activation/modulation by kinesore mitigated BPA-mediated mitochondrial dysfunction, bioenergetic deficits, and caspase activation in the hippocampal NSC–derived neurons

Normal mitochondrial trafficking and mitochondrial morphology help in maintaining stable and continuous ATP supply and neuronal homeostasis (18). Any defects in mitochondrial trafficking cause the accumulation of defective mitochondria and a reduction in energy production, leading to impairment in the generation of the proton gradient. We assessed the role of BPA-mediated impaired mitochondrial movement on mitochondrial functions, MMP, ATP levels, and caspase activation. BPA exposure significantly (∗*p* < 0.05) reduced MMP and ATP levels (Fig. 8*H*–*J*). Cotreatment with kinesore significantly mitigated the BPA-induced mitochondrial dysfunction, resulting in improved MMP and elevated ATP levels relative to BPA-treated neurons (∗*p* < 0.05). Kinesore treatment significantly increased MMP and ATP levels in neuronal cultures as compared with control cultures. These results highlight the role of KIF5A-mediated mitochondrial transport in maintaining mitochondrial bioenergetics under neurotoxic conditions.

Given the detrimental effects of KIF5A dysregulation on axonal mitochondrial transport and synaptic maintenance, previous reports have implicated KIF5A loss of function in various forms of neuronal injury (62, 63, 64). To assess whether restoring KIF5A expression confers neuroprotection against BPA-induced stress, we examined caspase-3 activation. BPA exposure significantly increased cleaved caspase-3 levels as compared with the control group (Fig. S9, *A*–*C*). The activation of KIF5A with kinesore attenuated BPA-induced caspase-3 cleavage (Fig. S9, *A*–*C*). Kinesore treatment alone, decreased cleaved caspase-3 levels as compared with controls, indicating the role of KIF5A-mediated mitochondrial transport in regulating neuronal survival under BPA-induced stress. Collectively, these findings indicate the involvement of Kinesin-1(KIF5A)-dependent processes in maintaining mitochondrial bioenergetics, which is supported by increased mitochondrial functions and neuronal survival, as observed by increased MMP & ATP levels and decreased cleaved caspase-3 levels by Kinesin-1 modulation by kinesore during BPA mediated stress conditions.

### Kinesin-1 (KIF5A) activation/modulation by kinesore restores BPA-mediated impaired mitochondrial transport, distribution, and synaptic functions in the rat brain hippocampus

**Figure 9:**
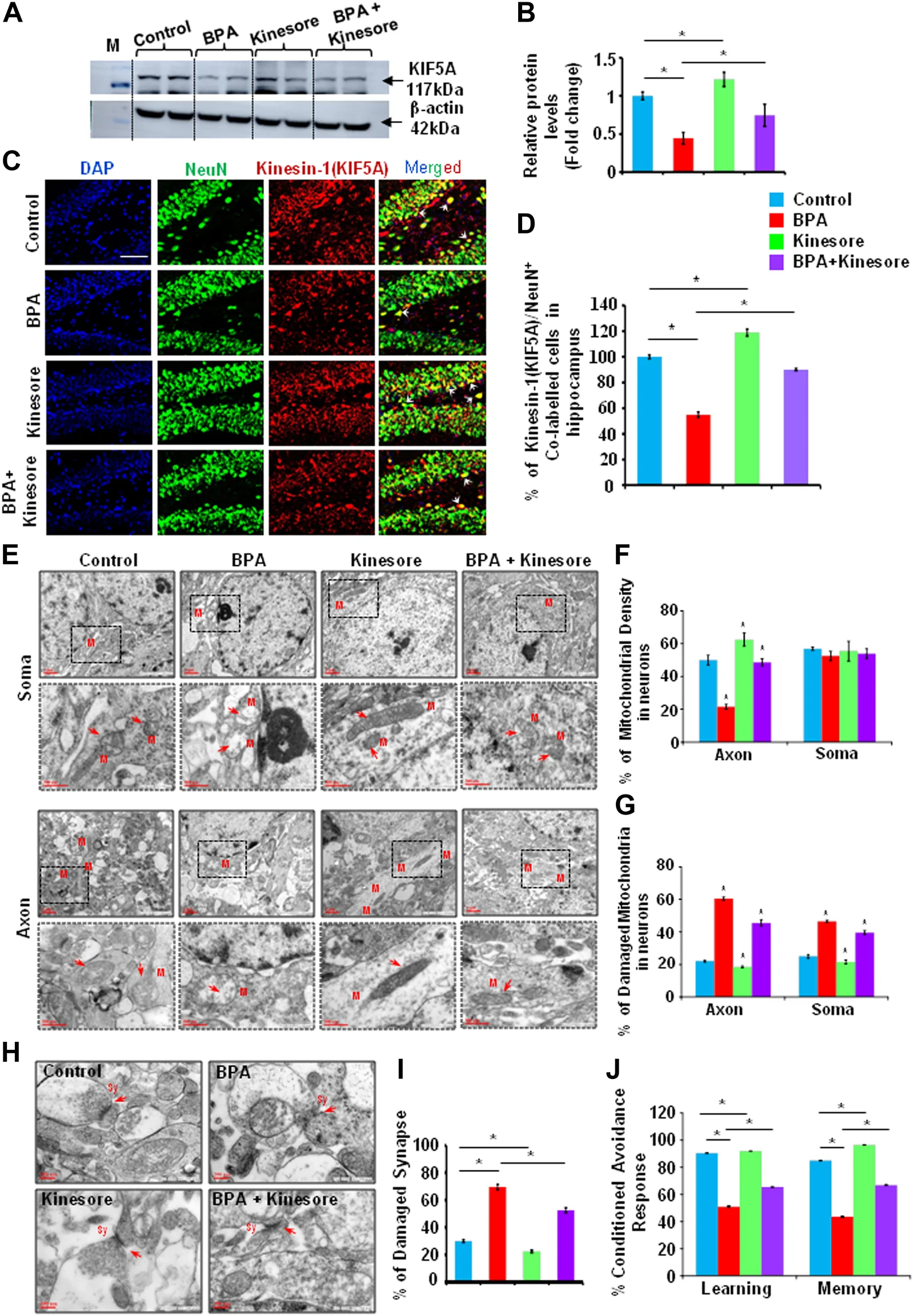
**Activation of kinesin-1 (KIF5A) by kinesore reverses BPA-induced impairments in mitochondrial transport, density, and synaptic integrity, leading to cognitive impairments in the hippocampus of the rat brain.** Effects of kinesore (50 mg/kg, i.p.; PND80–PND90) following BPA exposure (40 μg/kg, oral; PND21–PND90) on KIF5A expression, mitochondrial transport, density, and synaptic integrity in the rat hippocampus. *A* and *B*, immunoblot analysis showing reduced KIF5A protein levels following BPA exposure, increased KIF5A expression with kinesore treatment alone, and restoration of KIF5A levels in BPA-exposed rats following kinesore cotreatment. The data are represented as mean ± SEM (n = 6 rats/group). ∗*p* < 0.05. Marker is represented as M. *C* and *D*, double immunofluorescence colabeling of KIF5A with NeuN (neuronal marker) resulted in a significant increase in KIF5A expression in NeuN-positive hippocampal neurons after kinesore treatment in BPA-exposed rats. The data are represented as mean ± SEM (n = 5 rats/group, five sections/rat). ∗*p* < 0.05. The scale bar represents 50 μm. *E*–*I*, TEM analysis revealed reduced axonal mitochondrial density, increased damaged mitochondria with abnormal cristae, and synaptic ultrastructural abnormalities following BPA exposure, all of which were significantly attenuated by kinesore treatment, preserving mitochondrial organization and synaptic integrity. The data are represented as mean ± SEM (n = 3 rats/group). ∗*p* < 0.05. M , mitochondria (the scale bar represents 1 μm, mitochondrial density) (the scale bar represents 500 nm, inset showing magnified images of the selected area also revealed increased damaged mitochondria), and Sy, synapse (the scale bar represents 200 nm, damaged synapse). *J*, the two-way conditioned neurobehavioral active conditioned avoidance response (CAR) was measured to study the cognitive abilities in the BPA- and kinesore-treated rats. BPA exposure resulted in significant impairments in cognitive functions as compared with controls, related to learning and memory. Kinesore treatment significantly ameliorated BPA-induced cognitive deficits, reflected by an increased CAR percentage. The data are represented as mean ± SEM (n = 10 rats/group). ∗*p* < 0.05. BPA, bisphenol-A; NeuN, neuronal nuclei; PND, postnatal day; TEM, transmission electron microscopy.

Our previous *in vitro* findings suggested that activation of KIF5A by kinesore counteracts BPA-induced downregulation of KIF5A in hippocampal NSC–derived neurons (Fig. 7) To validate these observations *in vivo*, *Wistar* rats were orally administered BPA (40 μg/kg body weight/d) from PND 21 to 90 and received i.p. kinesin-1 activator kinesore (50 mg/kg body weight/d) during PND 80 to 90. Immunoblot analysis of the rat hippocampus revealed a significant reduction in KIF5A protein levels following BPA exposure, whereas kinesore treatment alone markedly increased KIF5A levels as compared with control (∗*p* < 0.05; Fig. 9 *A*, *B* and S10). Importantly, kinesore administration in BPA-exposed rats effectively restored KIF5A protein levels as compared with BPA-only treatment. Consistent with these findings, double immunofluorescence colabeling of KIF5A with the neuronal marker NeuN showed enhanced KIF5A expression in mature hippocampal neurons following kinesore treatment in BPA-exposed rats (∗*p* < 0.05; Fig. 9 *C* and *D*).

Furthermore, TEM analysis revealed that activation of KIF5A by kinesore restored BPA-induced reduced mitochondrial density in axons (∗*p* < 0.05; Fig. 9*E* and *F*). KIF5A activation also reduced the BPA-mediated increase in the number of damaged mitochondria with abnormal cristae (∗*p* < 0.05; Fig. 9*E* and *G*). Furthermore, kinesore treatment attenuated BPA-mediated synaptic damage and reduced synaptic integrity (∗*p* < 0.05; Fig. 9*H* and *I*). Therefore, KIF5A activation attenuated these BPA-induced ultrastructural abnormalities, preserving mitochondrial organization and synaptic architecture, thereby highlighting its neuroprotective role in maintaining mitochondrial trafficking and synaptic integrity *in vivo* (∗*p* < 0.05; Fig. 9*E*–*I*).

Collectively, these results corroborate our *in vitro* data and demonstrate that kinesore-mediated activation of KIF5A restores mitochondrial transport machinery, thereby mitigating BPA-induced disruptions in mitochondrial dynamics and supporting its role in hippocampal neurogenesis.

### Kinesin-1 (KIF5A) activation/modulation by kinesore mitigated BPA-mediated impairments in learning and memory in rats

The hippocampus region of the brain regulates and maintains cognitive functions, such as learning and memory, which are primarily governed by hippocampal neurogenesis (65). Therefore, we examined the effects of BPA and kinesore on cognitive functions in the rats by measuring conditioned avoidance response (CAR) using a shuttle box apparatus (Fig. 9*J*). We observed a neuroprotective effect of kinesore against BPA-induced cognitive impairments in rats. BPA exposure significantly (∗*p* < 0.05) reduced learning and memory performance, whereas kinesore treatment significantly (∗*p* < 0.05) improved cognitive function in BPA-treated rats, as indicated by the increased percentage of CAR (Fig. 9*J*). Overall, these findings suggest that kinesore mitigates BPA-mediated deficits in learning and memory, which may arise from disrupted mitochondrial trafficking leading to altered neurogenesis in the hippocampus, thereby highlighting its protective role in restoring BPA-induced cognitive deficits.

**Figure 10:**
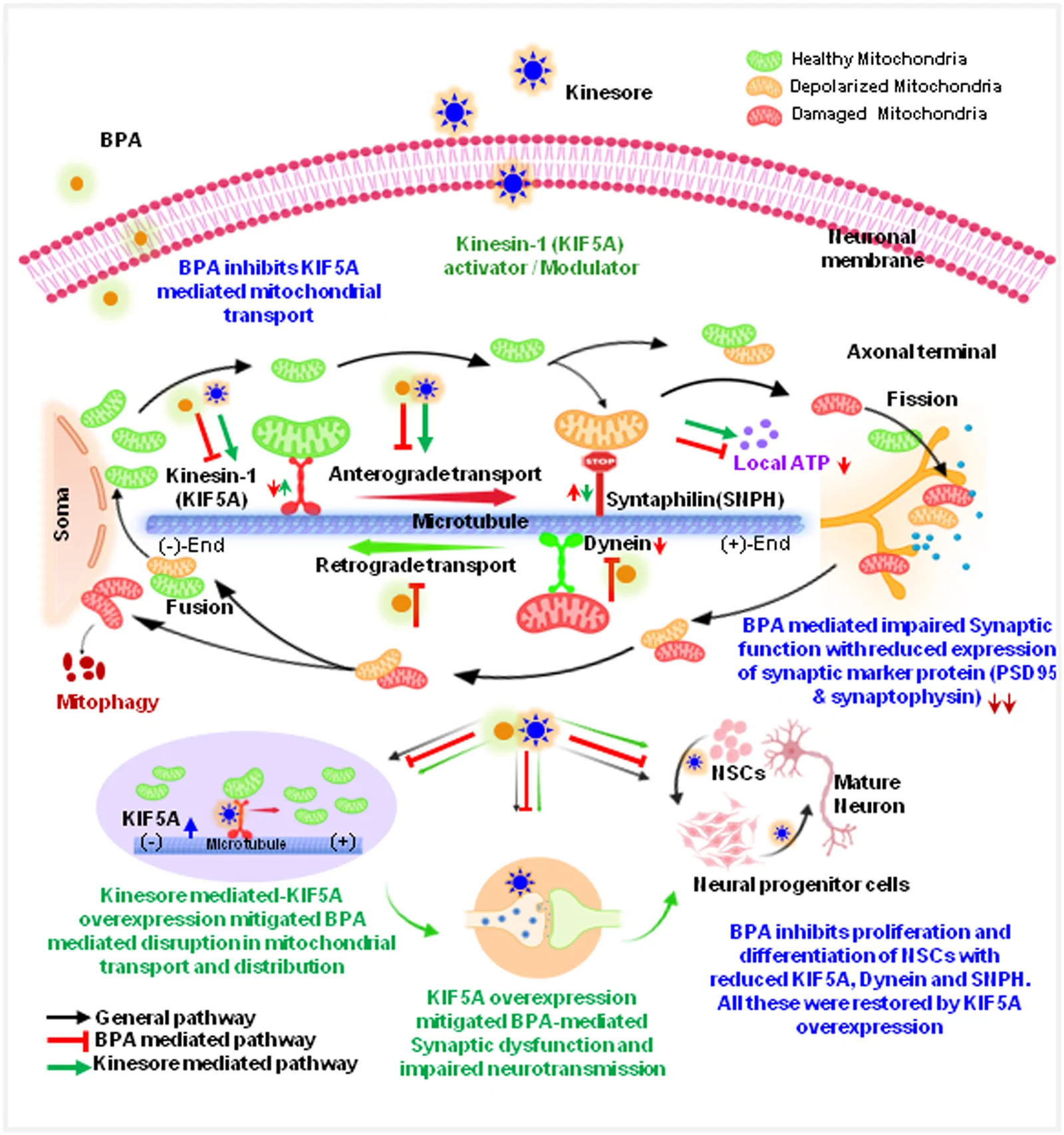
**Schematic representation of effects of BPA on mitochondrial transport and synaptic function.** BPA exposure impairs KIF5A-dependent mitochondrial transport, leading to synaptic dysfunction in the hippocampus region of the rat brain. Diagram depicting the general pathway (*black*), the BPA-mediated, KIF5A inhibition pathway (*red arrows*), and KIF5A activation pathway (*green arrows*). BPA reduces KIF5A and dynein levels, which further increases SNPH levels, resulting in impaired mitochondrial distribution and density, affecting mitochondrial transport and synaptic function during NSC proliferation and differentiation. KIF5A restoration *via* kinesore restores mitochondrial transport, ensures proper mitochondrial distribution/density, enhances synaptic function, and reduces apoptosis, thereby supporting neuronal survival and neurogenesis. BPA, bisphenol-A; NSC, neural stem cell.

Based on our experimental findings, we propose a schematic diagram illustrating the potential mechanisms by which BPA disrupts KIF5A, dynein, and SNPH-dependent mitochondrial trafficking, leading to altered mitochondrial distribution, abnormal morphology, and synaptic dysfunction (Fig. 10). BPA exposure downregulated/reduced motor proteins KIF5A and dynein levels, which increased the mitochondrial anchoring factor SNPH protein levels. The decreased levels of KIF5A and dynein resulted in impaired anterograde and retrograde mitochondrial trafficking in axons, thereby increasing SNPH levels, which arrest mitochondria inside neurons and halt trafficking. Impaired mitochondrial transport may be responsible for the failure of synaptic transmission and synaptic loss. Treatment with kinesore, a modulator of Kinesin-1 activity, mitigated BPA-induced impairments in mitochondrial transport, synaptic function, and bioenergetic deficits *etc,* during NSC proliferation and neuronal differentiation. Together, these findings indicate that mitochondrial trafficking is highly sensitive to alterations in motor system dynamics, and support the involvement of Kinesin-1(KIF5A)-dependent processes in maintaining mitochondrial distribution and neuronal function. These observations highlight the potential of targeting motor activity to mitigate BPA-induced neurotoxicity.

## Discussion

Proper regulation of mitochondrial trafficking ensures precise delivery to axons and synapses, meeting localized energy and calcium-buffering demands (8, 60). As most mitochondria originate in the soma, specialized trafficking systems transport them to distal sites, such as synaptic terminals (66, 67, 68). Disruption in mitochondrial dynamics, including fusion and fission, causes impaired axonal mitochondrial trafficking, mitochondrial deformation, and stalling, leading to the pathogenesis of neurological disorders, such as Alzheimer’s disease, Parkinson’s disease, and Huntington's disease (7, 26, 43, 69). During the normal neurodevelopmental process, BPA interferes with the brain and adversely affects various physiological processes (70, 71). BPA exposure causes impaired hippocampal neurogenesis/oligodendrogenesis and myelination, leading to enhanced neurodegeneration and cognitive impairments (29, 30, 31). BPA also inhibits GFER and Drp-1-associated mitochondrial biogenesis and dynamics (fission and fusion), which ultimately leads to impaired neurogenesis (40, 41, 42). Our current study suggested that BPA-induced neurotoxicity may be linked to disrupted mitochondrial trafficking, resulting in impaired neurogenesis in the rat brain. We observed that KIF5A is significantly involved in BPA-mediated alterations in mitochondrial transport/trafficking. Kinesore-mediated KIF5A activation/modulation mitigates BPA-mediated impaired mitochondrial transport and distribution during NSC proliferation and neuronal differentiation, leading to improved learning and memory in rats.

Mitochondrial trafficking ensures the anterograde movement of healthy mitochondria from the soma to nerve terminals, whereas damaged mitochondria are transported retrogradely for repair or degradation (72, 73). This bidirectional transport is mediated by motor proteins, such as kinesin-1 and dynein (57, 74). KIF5A is neuron specific and crucial for maintaining synaptic function by transporting mitochondria and synaptic vesicle precursors (57, 75, 76). Static anchor protein SNPH immobilizes mitochondria by anchoring them to microtubules (20, 57). BPA exposure caused a significant reduction in mitochondrial transport in both anterograde and retrograde directions in neurons differentiated from hippocampal NSCs, evident at ≥50 μM BPA. Collectively, these observations indicate that BPA exposure is associated with early alterations in mitochondrial trafficking that occur prior to detectable changes in MMP and morphology (fragmentation); however, in the broader context of BPA-induced cellular dysfunction, these trafficking changes are best interpreted as one of several contributing factors to subsequent mitochondrial and neuronal impairments rather than as an isolated or primary defect.

This impairment was further supported by a marked increase in the number of stationary mitochondria observed in BPA-treated neurons. Furthermore, within the experimental conditions examined, BPA exposure did not produce detectable alterations in lysosomal or endosomal trafficking, suggesting that the observed changes in mitochondrial transport are not accompanied by a uniform impairment of intracellular organelle dynamics and should therefore be interpreted cautiously within the broader context of BPA-induced cellular stress. Together, these findings suggest that inhibition of mitochondrial trafficking is an early and organelle-specific event that may subsequently lead to mitochondrial dysfunction, thereby contributing to BPA-induced neurotoxicity. Impaired mitochondrial trafficking causes alterations in mitochondrial distribution and mitochondrial dysfunction in neurons (43, 74). Next, we found that BPA significantly reduced mitochondrial density in neurons in cultures. TEM analysis revealed that BPA significantly reduces mitochondrial density and disrupts mitochondrial morphology within the axons of hippocampal neurons in the rat brain. This suggests that impaired mitochondrial distribution and altered transport are closely associated with BPA-induced neurotoxicity.

*In silico* studies predicted binding of BPA to key mitochondrial trafficking motor proteins, particularly KIF5A and dynein, while showing negligible interaction with KIF5B, KIF5C, and SNPH, suggesting its specific binding with KIF5A and dynein. The residue-dependent loss of binding affinity observed in alanine-scanning mutational analyses supports a specific interaction mechanism of BPA with these proteins. This suggests that BPA recognition involves defined binding hotspots on these motor proteins. This predicted specificity is further supported by an *in vitro* study, which demonstrated BPA-derived quantum carbon dot colocalization with cellular KIF5A at the protein level. These complementary computational and experimental approaches provide convergent evidence that BPA may directly target mitochondrial motor proteins, supporting a mechanistic framework in which BPA-mediated neurotoxicity involves disruption of mitochondrial trafficking through modulation of kinesin- and dynein-dependent transport pathways.

Based on preliminary evidence from *in silico* studies indicating a plausible interaction between BPA and mitochondrial motor proteins, we further validated these findings at the molecular level and investigated BPA-induced alterations in the gene expression and protein levels of mitochondrial trafficking regulatory machinery in the rat hippocampus. Interestingly, BPA also downregulated key motor proteins involved in axonal mitochondrial trafficking in rat hippocampal neurons. BPA significantly decreased relative gene expression and protein levels of motor proteins, KIF5A and dynein, and increased gene and protein levels of SNPH. Our *in silico* studies thus substantiate these findings on gene expression and protein levels, which suggest that BPA-mediated enhanced SNPH levels are independent of the binding of BPA with SNPH but may associated through binding with KIF5A. These findings further suggest the involvement of KIF5A in BPA-induced impairment of mitochondrial transport in hippocampal neurons. BPA-mediated downregulation of KIF5A resulted in pronounced defects in mitochondrial trafficking and distribution, accompanied by synaptic dysfunction.

NSC proliferation, neuronal differentiation (neurogenesis), and synaptogenesis are energy-dependent processes requiring ATP (6, 77). Any disruption in ATP production or mitochondrial function can impair neuronal generation and contribute to neurological disorders (6, 78). We observed that BPA markedly impaired mitochondrial transport during NSC proliferation and neuronal fate determination both *in vitro* and *in vivo*. Given the close interconnection between mitochondrial transport and synaptic function, we subsequently investigated the impact of BPA on synaptic activity. Any defects in mitochondrial trafficking in neurons are implicated in the pathogenesis of synaptic loss, which leads to several major neurodegenerative diseases (23, 69). Our study provided insights about BPA exposure–mediated mitochondrial trafficking disruption, highlighting the compromised function of KIF5A and its downstream impact on synaptic function. Furthermore, BPA significantly increased synaptic loss in the BPA-treated hippocampus and NSC-derived neurons in culture, whereas it decreased the dendritic and neurite length in cultures. TEM study revealed that BPA exposure significantly decreased synaptic vesicles with damaged membrane morphology, while increasing the number of damaged synapses in the BPA-treated hippocampus of the rat brain. This indicates that BPA-induced neurotoxicity is linked with the loss of mitochondria from synaptic regions, leading to increased synaptic loss and dysfunction in the hippocampus. These results are corroborated by earlier studies where disruption of mitochondrial transport impairs energy supply at synapses, leading to altered synaptic plasticity and neurotransmission (51). Furthermore, these synaptic deficits can compromise hippocampal function, thereby negatively affecting learning and memory performance in rats (79, 80).

Next, we studied the specific role of KIF5A in BPA-mediated impaired mitochondrial transport, density, and synaptic functions in coordination with impaired neurogenesis and neurite outgrowth. The activity of KIF5A is crucial for mitochondrial trafficking, and its inhibition leads to impaired mitochondrial distribution, reduced synaptic transmission, and neurodegeneration (55, 62). Disruption of mitochondrial transport adversely affects the metabolic and bioenergetic requirements of NSCs, thereby reducing their proliferation and self-renewal capacity (6, 81). KIF5A knockdown or mutation impairs mitochondrial transport, disrupts synaptic protein localization, and compromises neurotrophic signaling, contributing to axonal degeneration and motor neuron pathology (55, 64, 82). Similarly, the neurotoxicant paraquat impaired axonal mitochondrial transport through reduced KIF5A expression (83).

Importantly, kinesore has been described as an activator of Kinesin-1 at the molecular level by relieving autoinhibition and enhancing motor-microtubule interaction. However, at the cellular level its net effects on intracellular cargo transport are context-dependent (58). This study demonstrates that, in addition to activating motor activity, kinesore induces microtubule sliding and cytoskeletal reorganization, thereby altering motor behavior and intracellular transport dynamics. Consistent with this, kinesore shifts Kinesin-1 activity toward enhanced microtubule engagement. Kinesore modulates Kinesin-1 activity by promoting motor engagement with microtubules while altering cargo interactions. Consequently, increased microtubule engagement may either enhance cargo movement or lead to non-productive motor interactions, while microtubule sliding can compete with cargo transport processes. Thus, the net effect of kinesore in term of increased mitochondrial trafficking in our study may be context-dependent. In our experimental system, characterized by high intrinsic mitochondrial transport demand in neurons and BPA-induced impairment of mitochondrial trafficking during cellular stress conditions, kinesore treatment resulted in increased mitochondrial motility, improved distribution, and functional rescue. These findings suggest that, under our experimental conditions, enhanced motor engagement and activity may outweigh potential reductions in cargo-binding efficiency. These findings also suggest that the motor activation effect of kinesore may predominate over potential transport-disruptive effects, leading to an overall enhancement of mitochondrial trafficking. Thus, kinesore can alter Kinesin-1 motor behavior, its net effect on mitochondrial trafficking may vary depending on cellular context, and in our system was associated with improved mitochondrial transport. SNPH directly interacts with kinesin-1 to arrest mitochondrial movement in axons (84), suggesting a potential feedback mechanism. KIF5A activation recovered BPA-mediated KIF5A downregulation, triggering compensatory SNPH expression during mitochondrial transport in the hippocampal neurons. This result suggests that disruption of mitochondrial transport may induce SNPH upregulation to retain mitochondria locally, possibly as a compensatory response to trafficking deficits. These results were substantiated by a study where mitochondrial transport stress, such as rotenone exposure, modulated SNPH expression levels (56), indicating dynamic regulation of anchoring machinery under pathological conditions. Our results indicated that KIF5A is involved in BPA-mediated altered mitochondrial trafficking during NSC proliferation and differentiation. Furthermore, KIF5A maintains synaptic precursor transport and its density in neurons (54, 55). Moreover, KIF5A loss disrupts mitochondrial transport and distribution, thereby contributing to synaptic failure (55, 83). Our findings suggest that BPA-induced synaptic dysfunction, decreased MMP, ATP production, and associated cognitive impairments are linked to compromised KIF5A-dependent transport mechanisms. KIF5A inhibition exacerbated BPA-mediated disruption of synaptic density and mitochondrial function in hippocampal neurons, whereas activation of KIF5A by kinesore restored synaptic integrity and associated bioenergetic deficits. This preservation of synaptic organization is consistent with the observed improvement in learning and memory performance in BPA-exposed rats, emphasizing the functional importance of KIF5A-mediated mitochondrial trafficking in maintaining hippocampal-dependent cognitive functions. Our interpretation of KIF5A-dependent processes in BPA mediated impaired neurogenesis is supported by multiple independent lines of evidence, including KIF5A gene expression and protein levels analyses, decreased co-localization of KIF5A with mitochondrial and neuronal markers, and direct visualization of impaired mitochondrial trafficking using live-cell imaging approaches. In addition, ultrastructural analyses revealed reduced mitochondrial density and altered morphology in axonal compartments, consistent with disrupted mitochondrial transport. Functional rescue experiments using kinesore further support the role of Kinesin-1-dependent processes in regulating mitochondrial dynamics under BPA-induced stress conditions. Collectively, these complementary molecular, imaging, and functional neurobehavioral findings provide convergent evidence for the involvement of KIF5A in mitochondrial trafficking.

Taken together, our findings indicate that BPA exposure induces broad cellular dysfunction in the hippocampus, while also suggesting that disruption of KIF5A-dependent mitochondrial trafficking may contribute, in part, to impaired hippocampal neurogenesis.

## Conclusions

In conclusion, our findings suggest that BPA exposure is associated with neurotoxic effects accompanied by alterations in mitochondrial trafficking, which may contribute to a reduction in NSC proliferation and differentiation, as well as deficits in hippocampus-dependent cognitive functions in rats. The observed disruptions in mitochondrial movement, distribution, and synaptic functions appear to assocaited with Kinesin-1 (KIF5A)-dependent processes. Notably, modulation of KIF5A activity using kinesore partially attenuated BPA-associated alterations in mitochondrial trafficking and synaptic parameters during neuronal differentiation. Collectively, these results indicate that while BPA induces broader cellular dysfunction, changes in KIF5A-dependent mitochondrial trafficking may represent one contributing factor underlying impaired hippocampal neurogenesis, supported by multiple independent lines of evidence.

## Experimental procedures

### Chemicals/reagents

BPA (2, 2′-*bis* (4-hydroxyphenyl) propane or 4,4-(propane-2,2-diyl) diphenol (catalog no.: 239658), bovine serum albumin (catalog no.: A2153), osmium tetraoxide (OsO4) (catalog no.: O5500-1G), glutaraldehyde (catalog no.: G5882), paraformaldehyde (PFA) (catalog no.: 158127), and CelLytic MT Cell Lysis Reagent (catalog no.: C3228) were procured from Sigma–Aldrich. Primary antibodies such as rabbit anti-KIF5A (catalog no.: 21186-1-AP) from Abcam, rabbit anti-KIF5B (catalog no.: 21632-1-AP), rabbit anti-KIF5C (catalog no.: 25897-1-AP), rabbit anti-SNPH (catalog no.: 13646-1-AP), and rabbit anti-dynein (catalog no.: 12219-1-AP) were obtained from Proteintech. Dimethyl sulfoxide (DMSO) (catalog no.: D2650), poly-D-lysine (PDL) (catalog no.: D3939), epidermal growth factor (catalog no.: TC 228), and human recombinant basic fibroblast growth factor (catalog no.: TC290) were obtained from HiMedia. N-2 supplement (catalog no.: 17502048), B-27 supplement (catalog no.: A3582801), antibiotic–antimycotic, Hank’s balanced salt solution (catalog no.: 14170112), antibiotic–antimycotic solution (catalog no.: 15240062), and neurobasal serum-free medium (catalog no.: 21103049) were procured from Gibco BRL. Cell culture flasks (Nunc) and antifade Vectashield mounting medium with DAPI (4,6-diamidino-2-phenylindole dilactate) (catalog no.: H-1200) were purchased from Vector Laboratories (Vectashield). Mouse anti–5-bromo-2′-deoxyuridine (catalog no.: sc-32323) and mouse monoclonal anti–beta-actin antibody (C4) (catalog no.: sc-47778) were procured from Santa Cruz Biotechnology. Rabbit anti-NeuN (catalog no.: ABN78) was obtained from Merck Millipore. Rabbit anti–active cleaved caspase-3 (catalog no.: D175), anti-rabbit PSD95 (catalog no.: 36233S), and anti-rabbit synaptophysin (catalog no.: 5461S) primary antibodies from Cell Signaling Technology. Kinesore (catalog no.: 6664) was procured from Tocris Bioscience. Cell Titer-Glo Luminescent Cell Viability Assay Kit was used for ATP measurement (Promega). Western blotting immobilon Western chemiluminescent substrate (catalog no.: WBKLS0500) and polyvinylidene fluoride membrane were obtained from Chemicon, Millipore. TMRM (catalog no.: I34361, T668), TRI reagent or TRIzol, SuperScript first-strand complementary DNA (cDNA), cDNA synthesis kit, and Alexa Fluor 594 and 488–linked secondary antibodies (Molecular Probes; Invitrogen). MitoTracker Green (catalog no.: M7514) was purchased from Thermo Fisher Scientific.

### Animal treatment and experimental schedule

Adult male *Wistar* rats weighing between 160 and 180 g were procured from the institutional animal breeding facility. All experimental procedures were approved by the CSIR-Indian Institute of Toxicology Research, Institutional Animal Ethics Committee (IITR/IAEC/76/23). Animals were maintained at a temperature of 25 ± 2 °C in a humidity-controlled environment, with a standard pellet diet and water, under a 12-h light–dark cycle. The rats were randomly assigned to the different experimental groups:

#### Vehicle-treated control group

Rats treated with corn oil (vehicle) orally daily from PND21 to PND90.

#### BPA-treated group

Rats treated with BPA; 40 μg/kg body weight, in corn oil orally daily from PND21 to PND90.

#### Kinesore-treated group

Rats treated with i.p. injection of kinesin activator/modulator kinesore (50 mg/kg body weight/daily) for 10 days from PND80 to PND90.

#### BPA + kinesore-treated group

Rats treated with BPA; 40 μg/kg body weight, in corn oil orally daily from PND21 to PND90 and i.p. injection of kinesin activator kinesore (50 mg/kg body weight/daily) for 10 days from PND80 to PND90.

The 40 μg/kg body BPA dose in rats was chosen based on our previous studies, which showed that low-dose BPA increased DRP1 levels and decreased peroxisome proliferator–activated receptor gamma coactivator-1 alpha and GFER. This triggered excessive mitochondrial fission and fragmentation as well as aberrant and disturbed mitochondrial biogenesis and morphology (40, 41). Following completion of treatments, rats were deeply anesthetized and subsequently sacrificed for brain dissection. Hippocampus tissue was isolated for qRT–PCR and Western blot analysis. Rats were transcardially perfused for the immunohistochemical and TEM analysis.

### Primary hippocampal NSC culture and treatment

Hippocampal NSC–derived neuronal cultures were established based on our previously published studies (31). In brief, pregnant female *Wistar* rats during gestation days 12 to 14 were deeply anesthetized with a (3:1) ketamine and xylazine mixture. Hippocampal tissues were cut and chopped with a scalpel blade and then run through a syringe three times in sterile Hank’s balanced salt solution buffer. These tiny chopped tissue fractions were incubated in trypsin and later neutralized with trypsin inhibitor, followed by gentle trituration. Following centrifugation, the cells were placed in the neurobasal medium with 2 mM l-glutamine, 20 ng/ml epidermal growth factor, basic fibroblast growth factor, 1% antibiotic–antimycotic, 1% N-2 supplement, and 2% B-27 (30, 40, 41). Last, cells were plated in a 25 cm^2^ culture flask and placed in a CO_2_ incubator at 37 °C. Primary neurospheres were formed in 6 to 7 days in NSC culture. For the quantification of NSC proliferation and differentiation, neurospheres were trypsinized, and cell suspensions were seeded on PDL-coated chamber slides in neuronal differentiation medium, excluding mitogens and containing 100 ng/ml brain-derived neurotrophic factor. After a 14-day incubation period, the neuronal cultures were subjected to immunocytochemistry for the neuronal marker β-III tubulin.

#### Experimental groups

NSC-derived neuronal cultures were treated as follows:

#### Vehicle control group

Cultures treated with vehicle, DMSO for 24 h.

#### BPA-treated group

Treated with BPA (100 μM) in DMSO for 24 h.

#### Kinesore-treated group

Treated with kinesin-1 pharmacological activator/modulator kinesore (50 μM) in DMSO for 24 h (58).

#### BPA + kinesore cotreated group

Cotreated with BPA (100 μM) + kinesore (50 μM) in DMSO for 24 h.

Following different treatments, cultures were used for neurosphere growth kinetics assay, Western blotting, double coimmunolocalization immunocytochemistry studies for β-tubulin-III–KIF5A, β-tubulin-III–SNPH, synaptophysin–MAP2, PSD95–MAP2, fluorescence mitochondrial density analysis, time-lapse live-cell imaging analysis, ATP analysis, MMP study by flow cytometry analysis, and oxidative stress analysis.

### Time-lapse live-cell imaging of mitochondria and quantification

Time-lapse live-cell imaging of mitochondria was carried out as per earlier methods (55, 82, 85). In brief, a single-cell suspension of cultured neurospheres was formed using trypsin and gentle trituration. The resulting cells were plated in the neurobasal media, B27, and Glutamax, and placed at 37 °C in a 5% CO_2_ incubator. NSC-derived neuronal cultures were treated with different concentrations of BPA (25–200 μM) in DMSO for 24 h. For mechanistic understanding, cultures were treated with 100 μM BPA (30, 40, 41) in the presence and absence of the KIF5A pharmacological activator/modulator, kinesore (50 μM) in DMSO for 24 h (58). Control and treated neuronal cultures were incubated with mitochondrial fluorescence dye MitoTracker Green (200 nM) for 30 min, and images were taken at 13 to 17 days *in vitro*. Before imaging, the neurobasal media were replaced with fresh neurobasal media. The movement of fluorescently labeled mitochondria in axons was studied using an inverted confocal microscope (LSM 880; Zeiss). Time-lapse images were captured every 30 s for a total of 2 h under a 63× oil objective using a confocal inverted microscope with an on-stage incubator (37 °C and 5% CO_2_). The acquired image sequence was reconstructed into a 2-min time-lapse movie using the Zeiss imaging software. Representative frames corresponding to the original acquisition intervals were extracted from the time-lapse sequence for qualitative assessment, while the complete image series was used for quantitative analysis of mitochondrial trafficking. Axons were identified as processes extending two to three times longer than other neurites originating from the soma. Mitochondria were defined as axon-confined particles exhibiting strong fluorescence intensity relative to the background and possessing clearly defined edges. A mitochondrion was considered to be immobile if it remained static throughout the imaging duration. Movement was quantified only if the mitochondrial displacement exceeded the mitochondrial size (55). Directionality was defined as anterograde for movement toward the distal axon and retrograde for movement toward the soma. The mitochondrial movement in NSC-derived neurons in anterograde and retrograde directions, and the number of stationary mitochondria, was quantified in 15 microscopic fields using ImageJ (National Institutes of Health [NIH]) software. The results were depicted in percent control. Similarly, the mitochondrial fragmentation was quantified in hippocampal NSC–derived neurons following 24 h exposure to BPA (25–200 μM). Quantification of elongated and round-shaped mitochondria was performed using ImageJ software, as described previously (45). Furthermore, lysosomal trafficking was examined using LysoTracker Green (200 nM), and endosomal trafficking was assessed using the endocytic tracer FM4-64 (10 μM) for 30 min after BPA exposure. The movement of organelles was quantified following the same criteria applied for mitochondrial trafficking analysis.

### MMP analysis using TMRM flow cytometry

Hippocampal NSC–derived neuronal cultures were exposed to BPA (25–200 μM, dissolved in DMSO) for 24 h, after which MMP was assessed by flow cytometry according to the manufacturer’s protocol.

### Mitochondrial density

The effect of BPA on axonal mitochondrial density in hippocampal NSC–derived neuronal culture was studied in cultures at 14 days *in vitro* (86, 87). NSC-derived neuronal cultures in a chamber slide were treated with BPA in DMSO for 24 h. Cultures treated with DMSO only served as vehicle-treated cultures. After respective treatments, cultures were incubated with MitoTracker Green at a concentration of 200 nM for 30 min at 37 °C in 5% CO_2_ incubator. Axons were identified based on morphological characteristics, with long branches of thin and uniform diameter. Strong green fluorescence (MitoTracker) of mitochondria with clear edges in axons was taken for analysis of axonal mitochondria density. Fluorescence images were captured using a 63x oil objective of a confocal microscope (LSM 880; Zeiss). Quantitative analysis was performed by counting MitoTracker Green–positive cells across 15 randomly selected microscopic fields per experimental group. Each experiment was independently repeated three times. Cell counts from all replicates were averaged, and the data were presented as a percentage relative to the control group. Statistical analysis was performed using GraphPad InStat software (GraphPad Software).

### *In silico*/molecular docking analysis

To investigate the molecular interactions between ligands and mitochondrial trafficking proteins, a molecular docking study was carried out by AutoDock 4.2 software (41, 46). The BPA chemical structure (CID_6623) was obtained from the PubChem database (88), and the crystal structure of the target protein molecule was obtained from the Protein Data Bank. AutoDock was utilized to predict the best binding mode and calculate the binding free energy. Biovia Discovery Studio 4.5 Client was used to visualize the interactions between proteins and ligands. To further validate the docking results and strengthen our mechanistic understanding of BPA interactions with motor proteins, we performed *in silico* alanine-scanning mutagenesis, followed by AutoDock-based redocking for KIF5A, DYNC1I1, and DYNC1I2. This analysis allowed us to quantify the energetic contribution of individual binding-site residues and determine whether BPA recognition is driven by specific and structurally important interactions. Key BPA-contacting residues were identified from the initial binding poses and interaction maps. Residues involved in hydrogen bonding, hydrophobic packing, or aromatic interactions were selected for alanine substitution. Residues selected for alanine scanning: KIF5A: Thr330, Glu7, Ala270, Leu269, Lys328, Ile327, and Lys274; DYNC1I1: Val496, Asn615, Pro542, Val543, Trp618, Pro613, and Ala495; and DYNC1I2: Met388, Phe314, Ile331, Val361, Leu372, and Val323. Single-point alanine mutants were generated using SWISS-MODEL (47), followed by PDBQT conversion using AutoDock. All docking parameters (grid center, grid size, and exhaustiveness) were kept identical to the wildtype conditions to ensure rigorous comparison. Binding energy changes were quantified using: (ΔΔG_binding_ = ΔG_mutant_ − ΔG_wildtype_). A ΔΔG ≥+1.0 kcal/mol indicates a residue that is critical for stabilizing BPA within the binding pocket. Further *in vitro* biochemical validation of BPA–motor protein interactions was performed using BPA-derived quantum carbon dots in NSC-derived neuronal cultures (48). Neurons were incubated with BPA quantum dots, fixed, and colocalization with KIF5A and dynein was assessed by fluorescence microscopy.

### Gene expression analysis by qRT–PCR

The qRT–PCR analysis was performed to quantify the expression of genes involved in regulating axonal mitochondrial trafficking after BPA exposure, as described earlier (30, 39, 41). Total hippocampal tissue RNA from control and BPA-treated groups was used for reverse transcription using a SuperScript First-Strand cDNA Synthesis Kit. The gene expression was quantified using the SYBR Green chemistry in the Quant Flex 6 Sequence Detector System (PE Applied Biosystems). The β-actin was used as a normalizing control. The relative expression of target axonal mitochondrial trafficking motor genes was determined using the ΔΔCt method, in terms of control fold change.

### Protein-level analysis by Western immunoblotting

As previously mentioned, immunoblotting was used to measure the amounts of proteins involved in mitochondrial trafficking in both control and BPA-treated rats as well as in various treated culture groups (39, 40, 42). An equal quantity of protein (60 μg per sample) was loaded onto SDS-polyacrylamide gels for electrophoresis. Following protein transfer, membranes were blocked with blocking buffer for 2 h at room temperature and subsequently incubated overnight at 4 °C with primary antibodies, such as KIF5A (1:500 dilution), KIF5B (1:1000 dilution), KIF5C (1:1000 dilution), dynein (DIC1) (1:1000 dilution), SNPH (1:1000 dilution), PSD95 (1:1000 dilution), synaptophysin (1:1000 dilution), and active cleaved caspase-3 and β-actin (1:10,000 dilution). Following washing, the membranes were incubated with horseradish peroxidase–tagged secondary antibodies (1:5000 dilution) for 2 h. Immunoreactive protein bands were visualized using a chemiluminescent substrate (Millipore), and densitometric analysis was carried out using ImageJ software to quantify relative protein expression levels.

### Colocalization of proteins by immunocytochemistry in NSC cultures

To assess the effects of BPA on NSC proliferation and neuronal fate determination, immunocytochemistry was performed using a previously described protocol (39, 40, 42). Briefly, neurospheres were dissociated into a single-cell suspension and plated on PDL-coated 4-well chambered slides. Following fixation with 4% PFA and incubation in blocking solution, the cells were incubated with primary antibodies, including mouse anti-Nestin (1:250 dilution), mouse anti-TOMM20, mouse anti-β-tubulin III (1:250 dilution), rabbit anti-KIF5A (1:200 dilution), rabbit anti-dynein (DIC1) (1:250 dilution), and rabbit anti-SNPH (1:250 dilution). The next day, the corresponding Alexa Fluor (488 and 594)–conjugated secondary antibodies were used for 2 h at room temperature. Last, the chamber slides containing cultures were mounted using DAPI (antifade mounting media). The colocalization of two different proteins in cultures was studied using ImageJ software and the JACoP plugin. Furthermore, the colocalization of two distinct markers, Nestin, TOMM20, and β-tubulin III, with KIF5A and dynein as measured using Mander’s colocalization coefficient (M), where M = 1 indicates perfect colocalization. The results were shown as a percentage of colocalization, calculated using the mean of Mender's coefficient value, M, over three repeated individual experiments.

### Colocalization of proteins by immunohistochemistry in the brain

Immunohistochemical analysis of proliferating and differentiating cells was carried out in the dentate gyrus of the hippocampus. Following transcardial perfusion with 4% PFA under deep ketamine/xylazine (3:1) anesthesia, the brains were postfixed in 4% PFA at 4 °C for 24 h. Next, brains were placed overnight in a 10% to 30% sucrose gradient. The serial 20 μm-thick coronal sections from the hippocampus were cut by freezing microtome (Cryostat; HM525; Microm), and every sixth section, 180 μm apart from each other, was kept in a blocking buffer containing 0.5% bovine serum albumin and 0.1% Triton X-100 for 2 h (40). The sections were then incubated overnight at 4 °C in primary antibodies, such as anti-Sox-2 (1:500 dilution), anti-KIF5A (1:200 dilution), anti-dynein (DIC1) (1:200 dilution), anti-SNPH (1:200 dilution), and anti-NeuN (1:500 dilution). Sections were then incubated with Alexa Fluor 594- or 488-conjugated secondary antibodies (1:200 dilution) for 2 h at room temperature. Subsequently, sections were mounted on gelatin-coated glass slides and coverslipped using antifade mounting medium containing DAPI. For the quantification of KIF5A–Sox-2, dynein–Sox-2, SNPH–Sox-2, KIF5A–NeuN, dynein–NeuN colocalized cells, the fluorescence images were acquired and analyzed using a fluorescence microscope (Eclipse Ti-S, inverted; Nikon) and NIS Elements BR imaging software (Nikon), respectively. The dentate gyrus was detected at low magnification (10×), and an outline was created. The degree of colocalization of two fluorescent markers (red and green) was quantified using Mander’s colocalization coefficient (M), where M = 1 indicates perfect overlap *via* the JACoP plugin in Image. Quantification was performed on five sections per rat (n = 5 rats/group), and results were expressed as the average percentage colocalization based on Mander’s coefficient across all sections analyzed for each group within the dentate gyrus region of the hippocampus, following the methodology established in our earlier work (30, 40, 42).

### Neuronal synaptic functions

After BPA treatment, cultured hippocampal neurons in a chamber slide were fixed in 4% PFA and incubated for 2 h in blocking solution. The cultures were incubated with primary antibodies, such as rabbit anti-synaptophysin (1:200 dilution), rabbit anti-PSD95 (1:200 dilution), and mouse anti-MAP2 (1:200 dilution). The following day, the Alexa Fluor (594 and 488)–tagged secondary antibodies were applied to the culture and incubated for 2 h at room temperature. Last, the neurons were mounted using DAPI (antifade mounting media). Fluorescence images were acquired and analyzed with ImageJ software.

### Neurite measurement of primary hippocampal neurons

Primary hippocampal neurons were treated with 100 μM BPA in 0.1% DMSO for 24 h. After treatment, the cells were fixed and blocked, followed by incubation overnight at 4 °C with rabbit anti-MAP2 primary antibody (1:250 dilution) and later with Alexa Fluor 488 secondary antibody (1:500 dilution) for 2 h at room temperature. Fluorescent images were acquired, and the total neurite length was quantified by Sholl analysis using the NeuronJ plugin in Fiji (Win) software (89, 90).

### Flow cytometry for MMP assessment

After respective treatments, the MMP of hippocampal NSC–derived neurons was examined through flow cytometry using the JC-1 kit in accordance with the manufacturer’s instructions.

### Functionality of mitochondria assessment by ATP measurement assay

The effect of BPA treatment on the functionality of mitochondria in NSC-derived neuronal cultures was studied using an ATP measurement assay. The ATP levels were measured with the CellTiter-Glo Luminescent Cell Viability ATP Assay Kit (Promega). In short, 1 × 10^6^ cells were seeded, and after treatment, a reaction buffer containing luciferin and substrate was added and incubated for 10 min. The luminescence was assessed using a microplate reader. ATP concentrations were determined using a standard curve and expressed as a percentage relative to the control group.

### Ultrastructural morphological changes of mitochondria and synaptic assessment by TEM

We studied the effects of BPA on mitochondrial trafficking, including the mitochondrial number in axons and soma, the number of damaged synapses, and the number of synaptic vesicles, at the ultrastructural level using TEM analysis in the control and BPA-treated rat’s hippocampus. Following the transcardial perfusion with 4% PFA, the brains were fixed in 0.1% glutaraldehyde solution. The small 2-mm pieces of rat hippocampal tissue were postfixed with 1% OsO4 for 2 h at room temperature after fixing with 2.5% glutaraldehyde at 4 °C overnight. After the removal of OsO4, the pieces were first dehydrated in a gradient of acetone (10–100%) and then again dehydrated in propylene oxide. The pieces were then baked at 65 °C for 48 h, followed by embedding in a mixture of araldite and dodecenyl succinic anhydride. The 50 to 70 nm ultrathin sections were cut and stained with uranyl acetate and lead citrate. In the rat hippocampal region, a minimum of 200 mitochondria with changed cristae shape were assessed, with three animals per group, both in axons and soma. Quantification of synaptic vesicles and morphological examination of synapses were carried out using Fiji software. In total, 150 synapses were analyzed from 10 randomly selected microscopic fields per group, with results presented as mean ± SEM, comparing the hippocampus of BPA-treated rats to controls.

### Neurobehavioral analysis by CAR

To evaluate the effects of BPA exposure in the presence and absence of kinesore on neurobehavioral function in PND-90 rats, learning and memory were tested using a shuttle-box apparatus (PACS-30; Columbus Instruments) (29). This apparatus consists of two connected chambers that hold a conditioned stimulus, an electric buzzer, and a light, each lasting 10 s, followed by an unconditioned stimulus, a mild foot shock (0.5 mA for up to 4 s), which was used for CAR analysis. Each group's rats were placed on one side of the shuttle box's chamber, and then they were given repetitive 20 trials/d, until the control group achieved 90% CAR. When the control group's learning ability reached 90% during the trial sessions, a comparison was made with the treatment groups. The control and treatment groups' cognitive abilities were measured using the percentage change in CAR. After 7 days, the memory ability assessment was done by measuring CAR. The learning and memory abilities of the treated rats were quantified and expressed as a percentage relative to the control group.

### Statistical analysis

Data were presented as mean ± SEM. Statistical comparisons between groups were conducted using an unpaired Student’s *t* test and one-way ANOVA, followed by Tukey–Kramer post hoc tests for multiple comparisons among groups. Analyses were carried out using GraphPad InStat software, version 5.0 for Windows, with statistical significance measured at *p* < 0.05.

## Data availability

All relevant data supporting/substantiating the conclusions of this study are included within the article and its supporting information.

## Supporting information

This article contains supporting information.

## Conflict of interest

The authors declare that they have no conflicts of interest with the contents of this article.
