# Supplementary material for: Bisphenol-A impairs hippocampal neurogenesis by disrupting kinesin-1-dependent mitochondrial trafficking
Source: J Biol Chem. 2026 Mar 14;302(9):111375. doi: 10.1016/j.jbc.2026.111375 (PMC13615719; doi:10.1016/j.jbc.2026.111375)
Supplement: Supplementary file 1 [file mmc1.pdf]

Bisphenol-A impairs hippocampal neurogenesis by disrupting Kinesin-1-dependent mitochondrial trafficking

Phoolmala[1,2], Saurabh Tiwari[1,2], Ranjeet Kumar Yadav[1,2], Shweta Singh Chauhan[2,3], Rajnish Kumar Chaturvedi[1,2*]

[1]Molecular Neurotoxicology and Cell Integrity Laboratory, Systems Toxicology and Health Risk Assessment Group, FEST Division, CSIR-Indian Institute of Toxicology Research (CSIR-IITR), Vishvigyan Bhavan, 31, Mahatma Gandhi Marg, Lucknow 226001, Uttar Pradesh (U.P.), India
[2]Academy of Scientific and Innovative Research (AcSIR), Ghaziabad, 201002, India
[3] Computational Toxicology Facility, Toxicoinformatics Research Group, CSIR-Indian Institute of Toxicology Research (CSIR-IITR), Vishvigyan Bhavan, 31, Mahatma Gandhi Marg, Lucknow 226001, Uttar Pradesh (U.P.), India

Keywords Bisphenol-A (BPA); Xenoestrogen; Neurogenesis; Hippocampus; Mitochondrial trafficking machinery; Kinesin-1 (KIF5); Dynein; SNPH; Neurotoxicity

***To whom correspondence should be addressed:**

Dr. Rajnish Kumar Chaturvedi
Molecular Neurotoxicology and Cell Integrity Laboratory
Systems Toxicology and Health Risk Assessment Group
FEST Division
CSIR-Indian Institute of Toxicology Research (CSIR-IITR)
Vishvigyan Bhavan,31, Mahatma Gandhi Marg, Lucknow, Uttar Pradesh 226001, India
Tel: +91-522-6228227
E-mail: rajnish@iitr.res.in, itrcrajnish@gmail.com

Supplementary Fig. S1

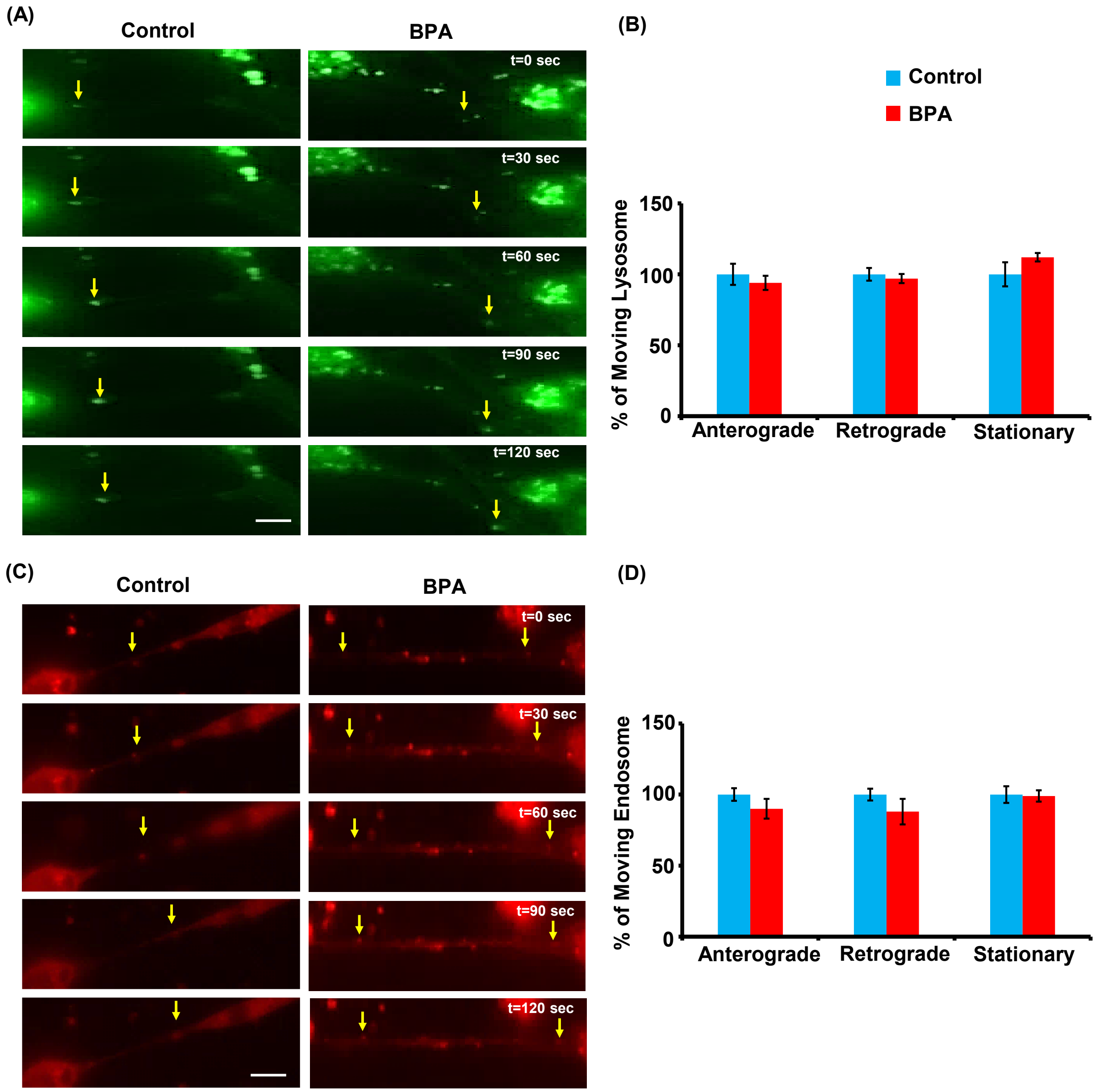

Supplementary Fig. S2 (A)

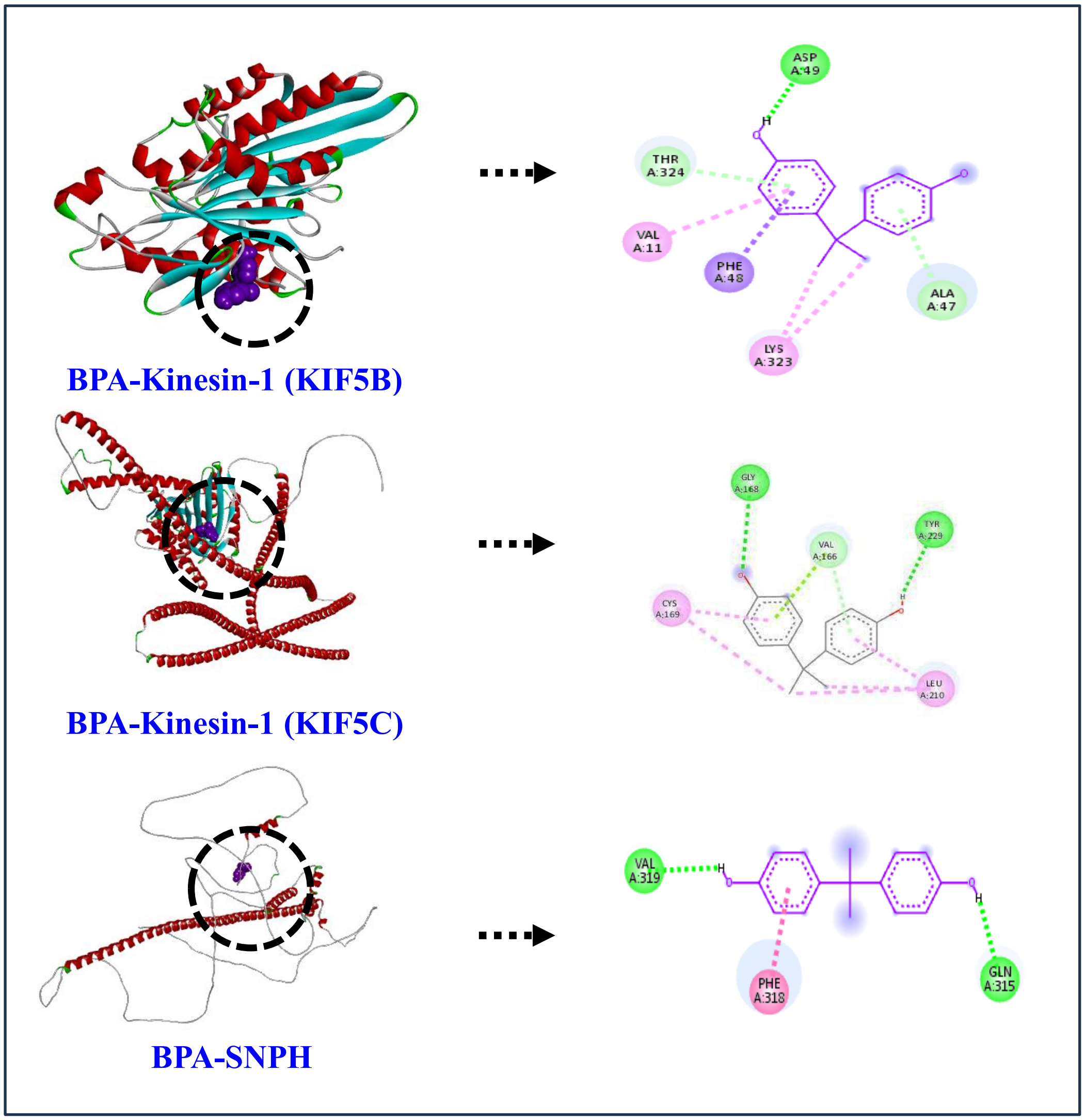

Supplementary Table S1: Interacting Amino Acids of BPA with Kinesin-1 isoforms and SNPH.

Target	Ligand	Binding Energy (kcal/mol)	Interaction Amino acids
Kinesin-1 (KIF5B)	BPA	-5.02	Ala47, Thr324, Phe48, Lys323, Val11
Kinesin-1 (KIF5C)	BPA	-4.42	Gly168,, Val166, Tyr229
Syntaphilin (SNPH)	BPA	-4.78	Gln315,Val319,Phe318

Supplementary Table S2:

S.No.	Primer Name (Oligo Name)	5'<-----Sequence----->3'	Length
1.	KIF5A (R) (Forward)	5’CACGACTCAGGAGCAGGTTT3'	20
	KIF5A (R) (Reverse)	5'CACGACTCAGGAGCAGGTTT3'	20
2.	kif5B (R) (Forward)	5’GAACACCGACACACATTGCAG3'	21
	kif5B (R) (Reverse)	5’GGCACCACTGTCCCTATGTT3'	20
3.	kif5C (R) (Forward)	5'TCTGGGCGAGATAGGTGGAA3'	20
	kif5C (R) (Reverse)	5'GATCTTGGCTTCATGCTGCG3'	20
4.	Dynein (R) (Forward)	5’ACGCAGAGTTCCAGCACTTC3’	20
	Dynein (R) (Reverse)	5’AGCCAAATGCCAAACTGCTCATC3’	23
5.	Syntaphilin (R) (Forward)	5’GAGCAATCCTGCTACCCTCC3’	20
	Syntaphilin (R) (Reverse)	5’CAGGTGCCGGATACAAACCT3’	20
6.	Syntabulin (R) (Forward)	5’CTGCTCAGACGACACAGGTT3’	20
	Syntabulin (R) (Reverse)	5’TGTTGCGTGAGAAAGAGGCT3’	20
7.	RanBP2 (R) (Forward)	5'CCGAGGTGGAGCGGTACAT3'	19
	RanBP2 (R) (Reverse)	5'TAGGGTCCCTCTCTTGCACA3'	20
8.	OGT (R) (Forward)	5’CCGCCATTTCAAGACAGTGC3’	20
	OGT (R) (Reverse)	5’TGTCACCTGCTGCTACCAAG3’	20
9.	Myosin 5a (R) (Forward)	5’GTGCGGTTACGAGACAGGAA3’	20
	Myosin 5a (R) (Reverse)	5’TGCTTGGGTAGCCCTTCAGA3’	20
10.	Myosin 5b (R)(forward)	5’GGGTCATGTCGTACAGCGAG3’	20
	Myosin 5b (R)(Reverse)	5’GCTGCTATTGTCGTTGCGAG3’	20
11.	MAP1B (R) (Forward)	5'AGAGCCAACCACAGATGACG3'	20
	MAP1B (R) (Reverse)	5'ATCCTGGGTGTAGGATGGCT3'	20

Supplementary Fig. S2 (B)

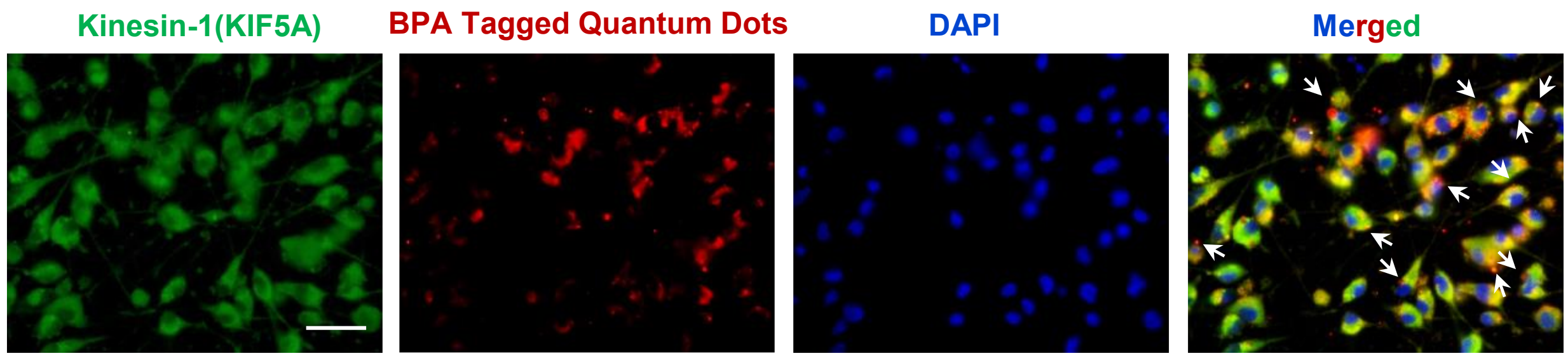

Supplementary Fig. S3

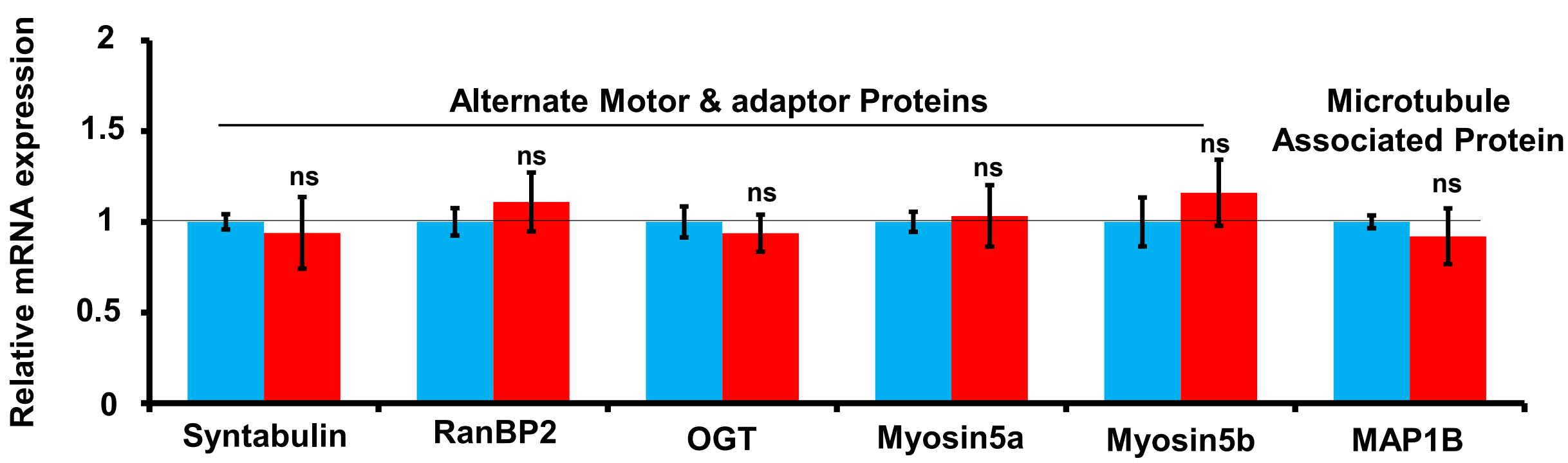

Supplementary Fig S4 (A)

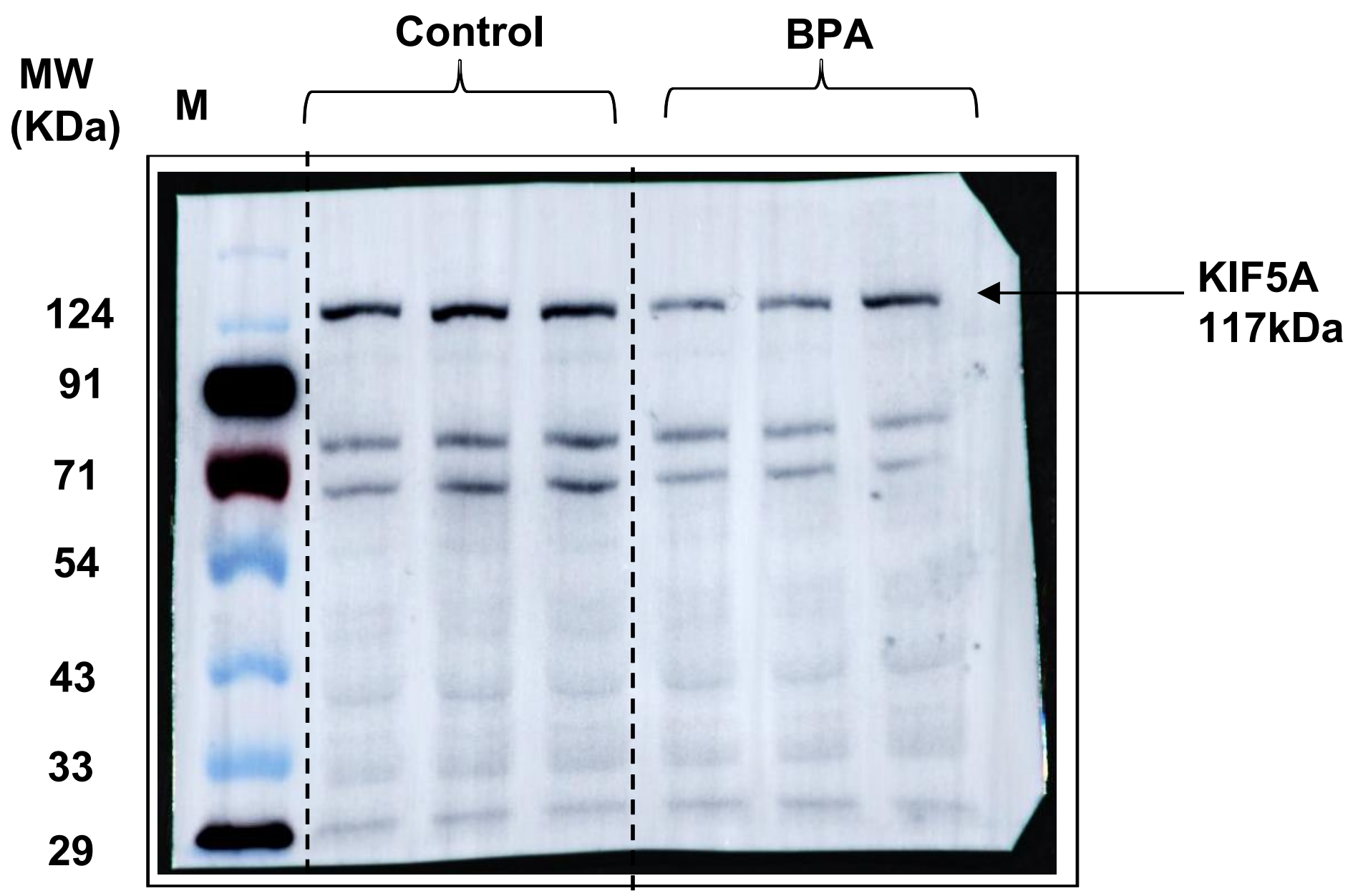

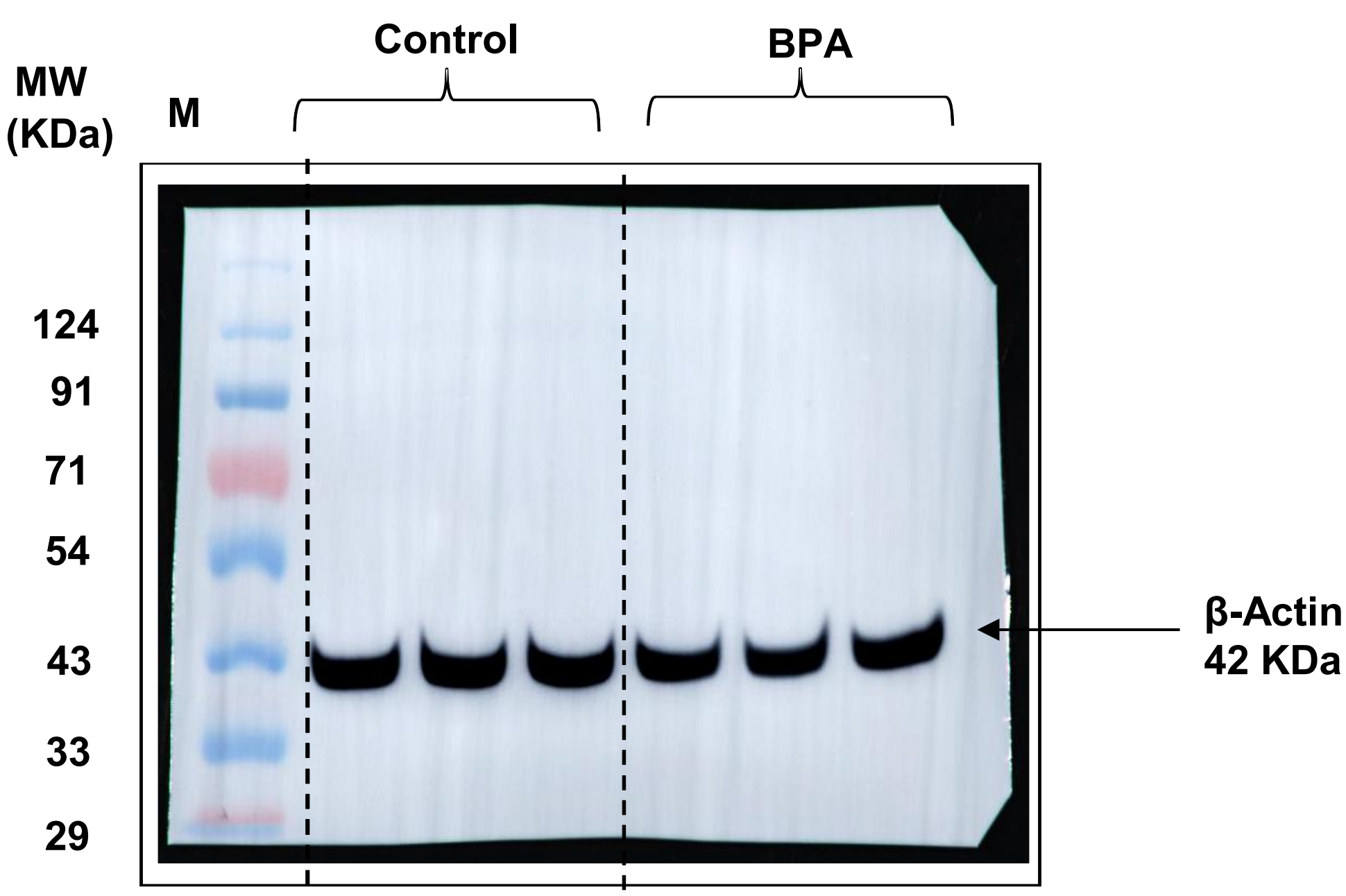

Supplementary Fig S4 (B)

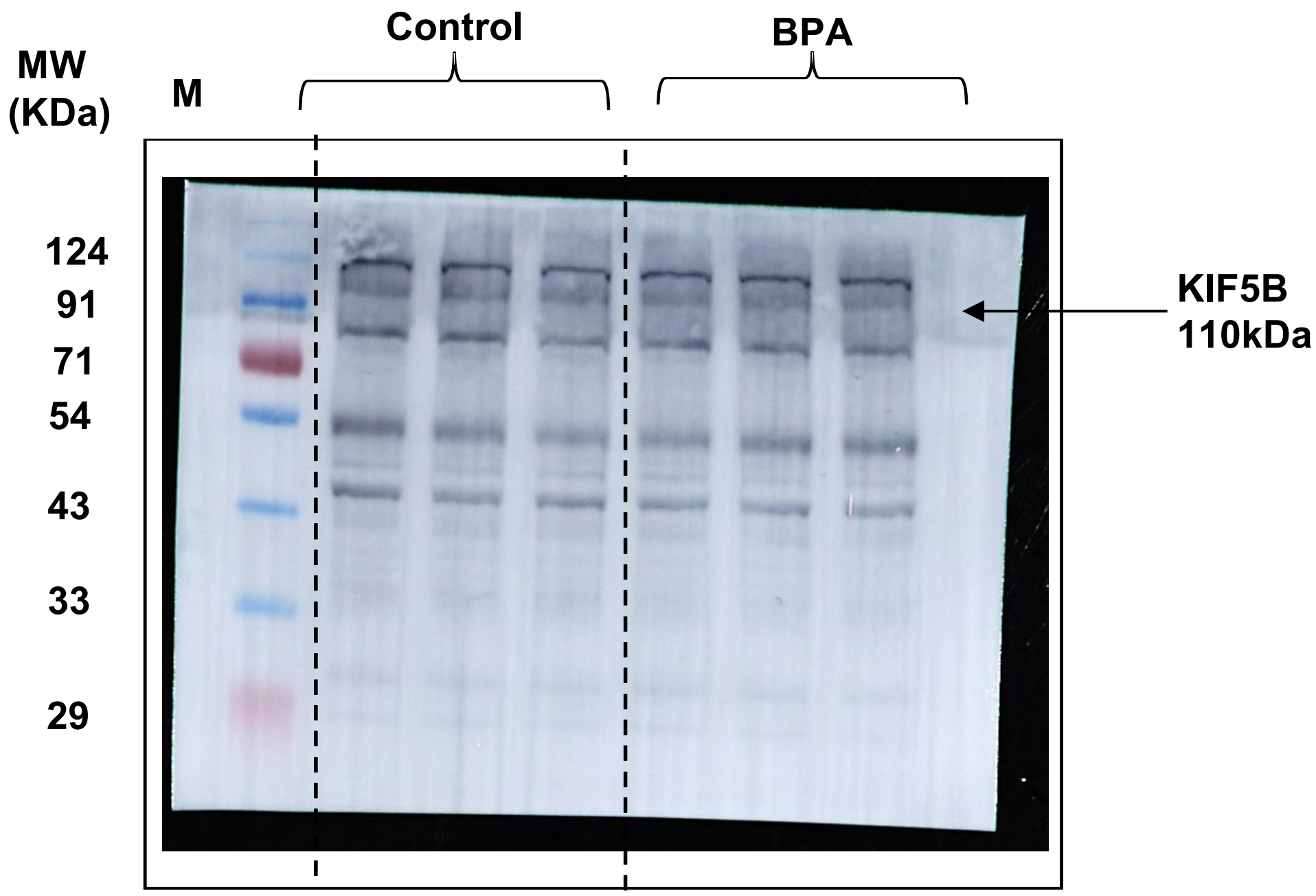

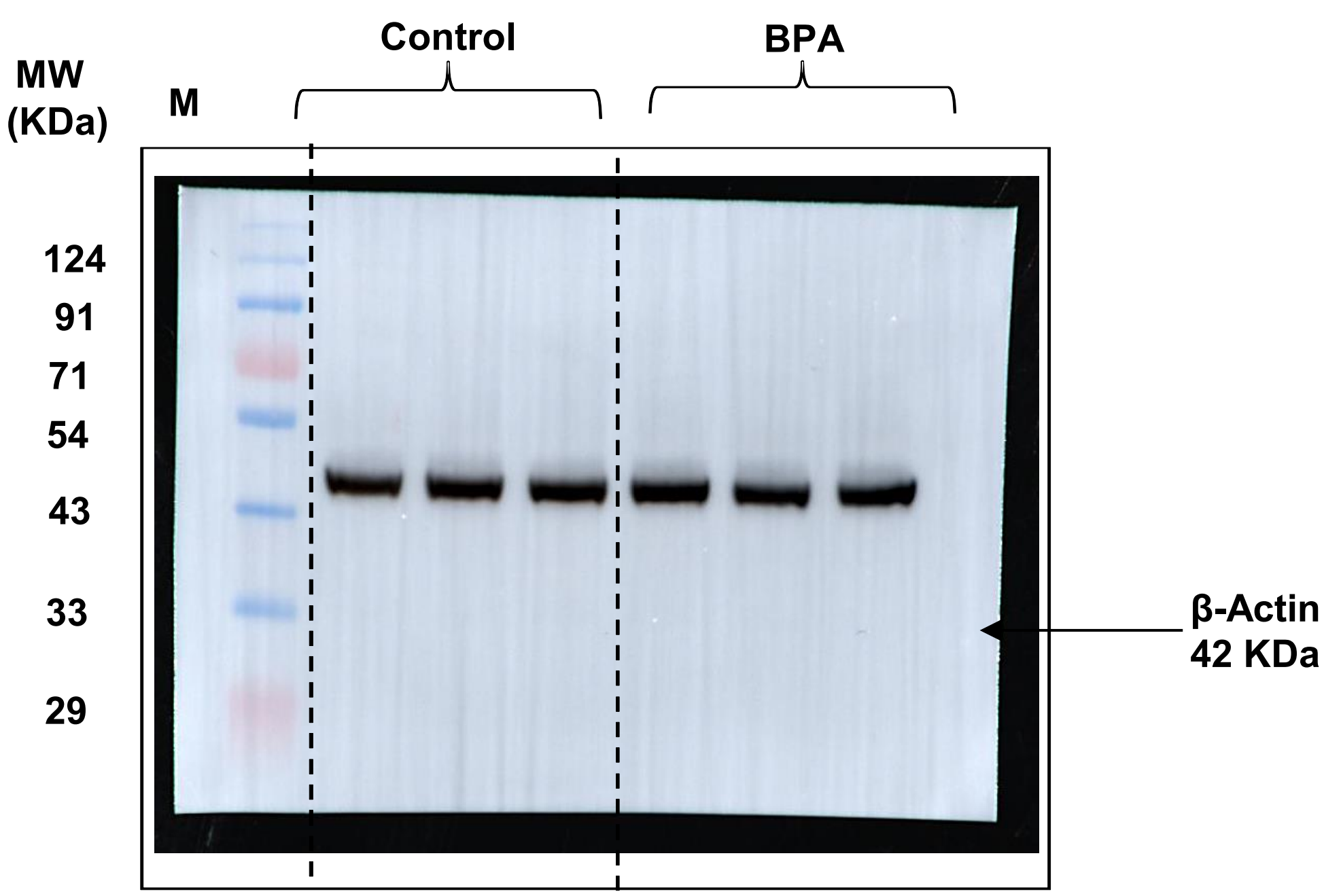

Supplementary Fig S4 (C)

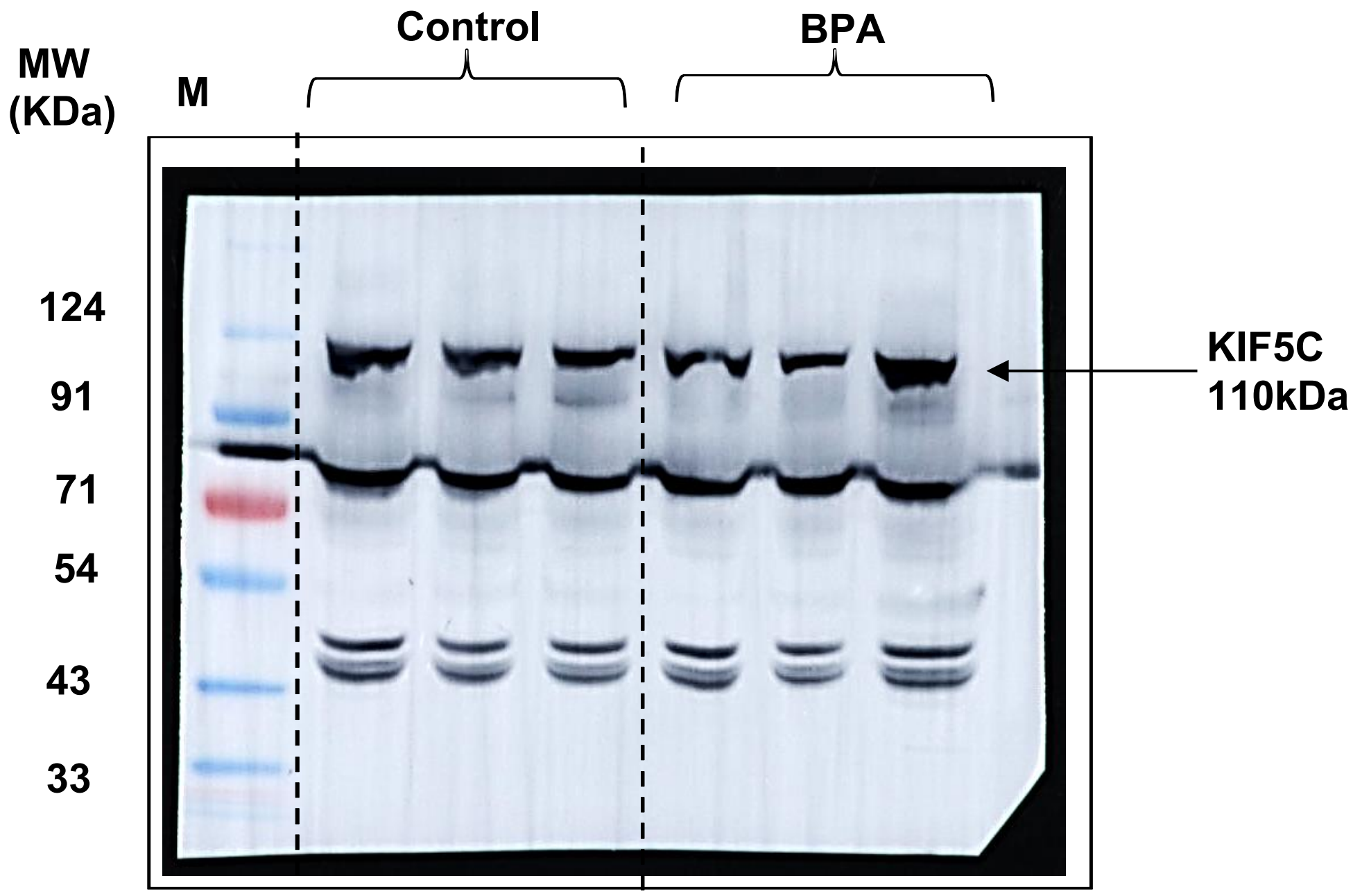

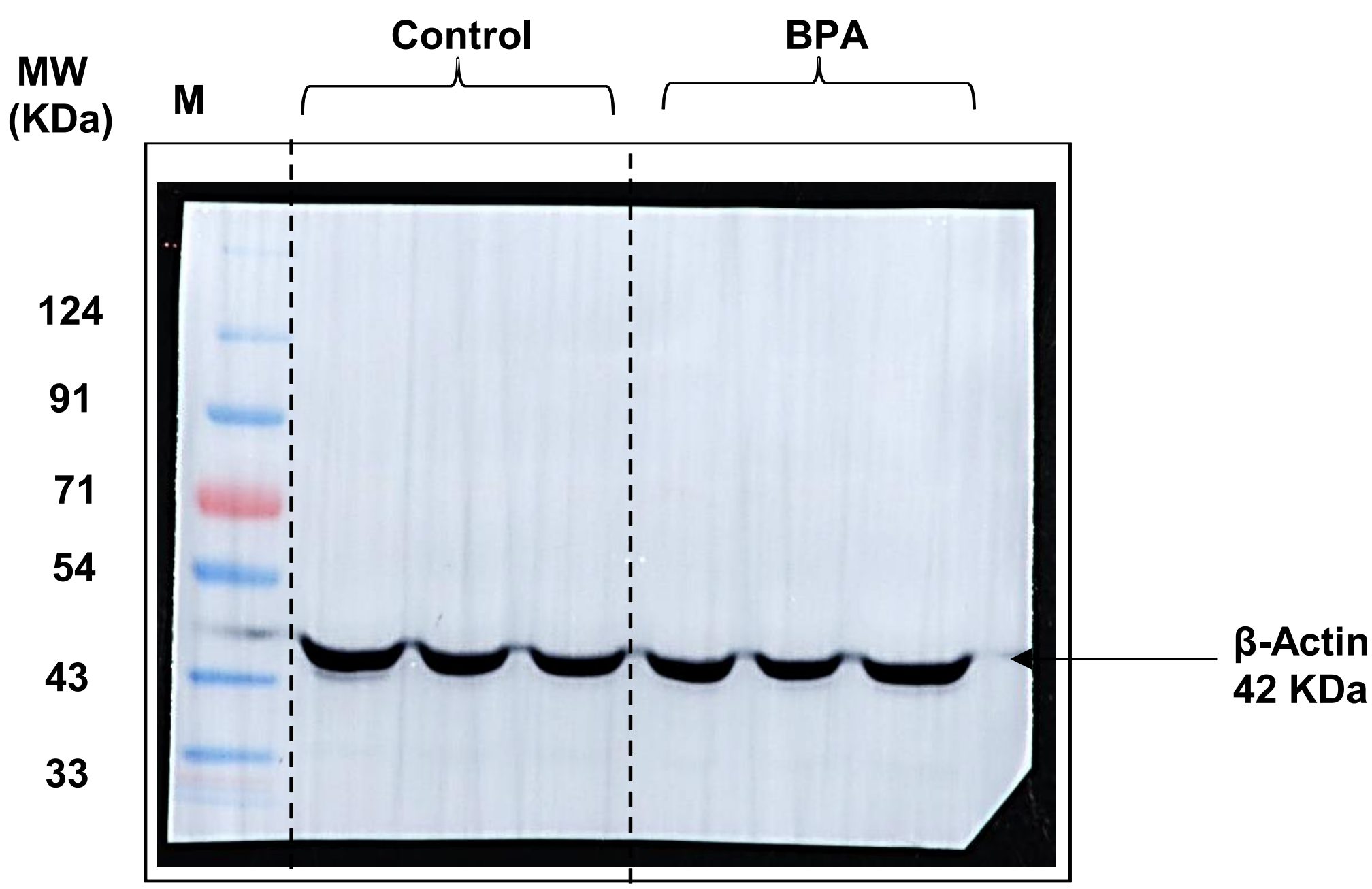

Supplementary Fig S4 (D)

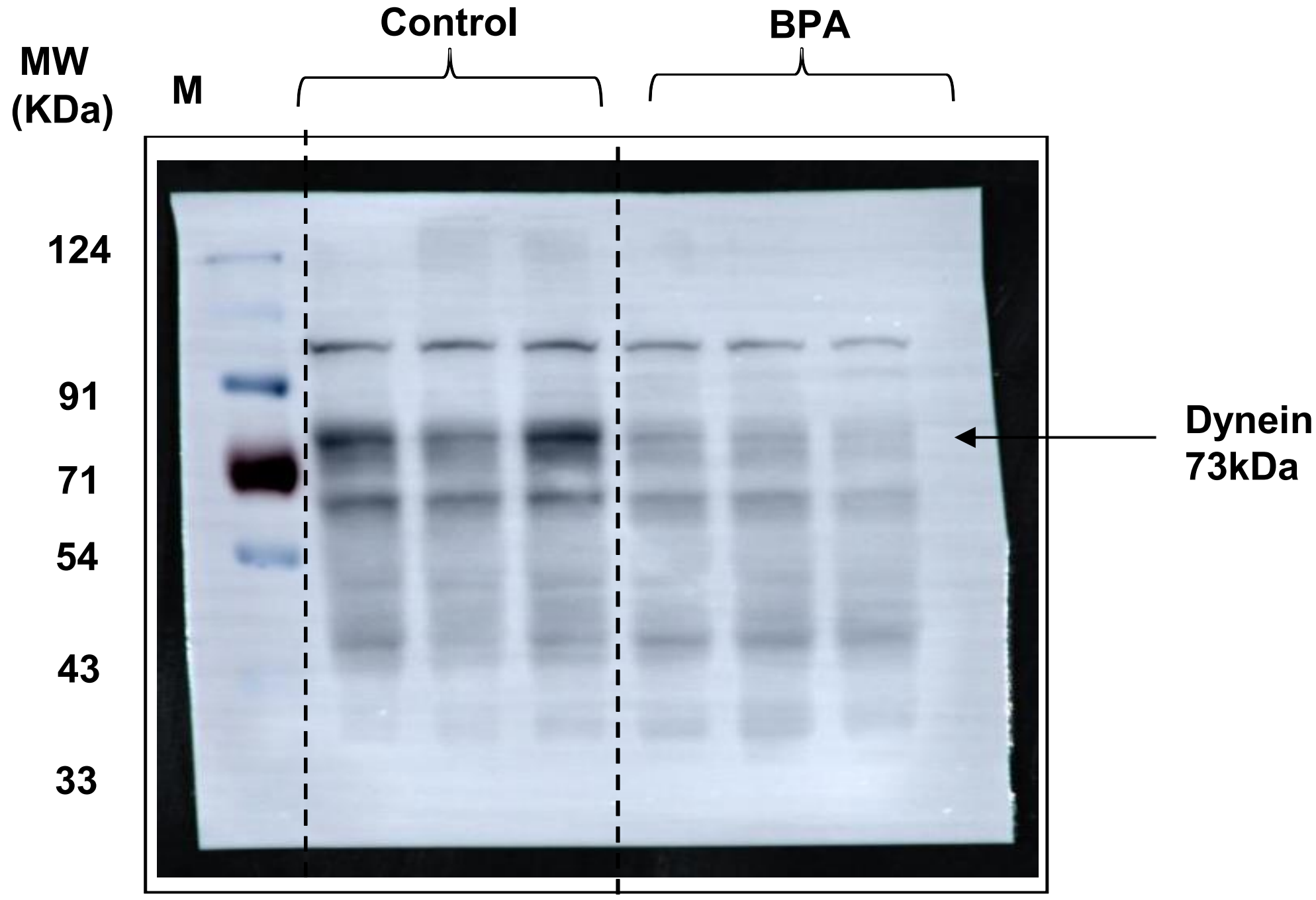

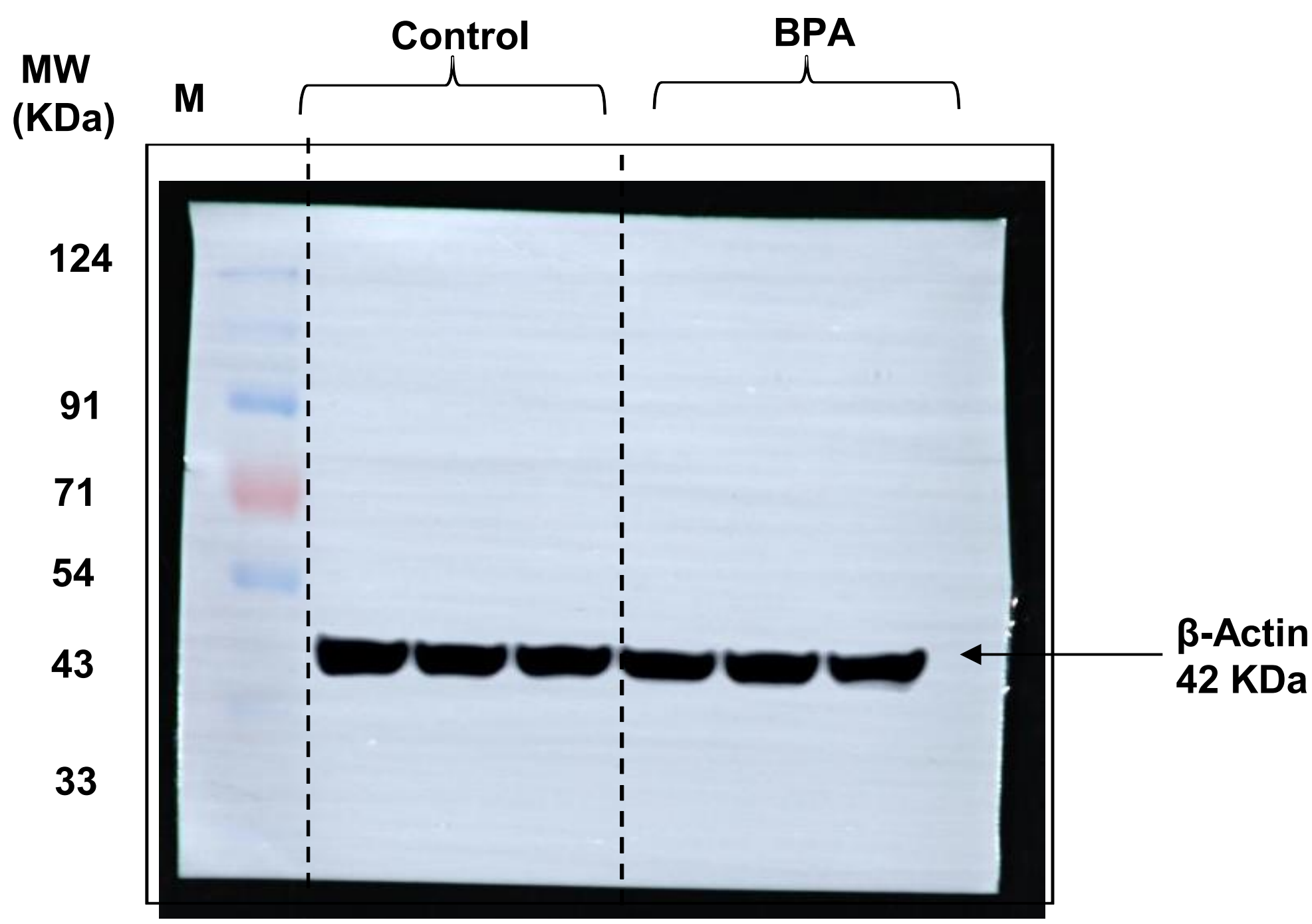

Supplementary Fig S4 (E)

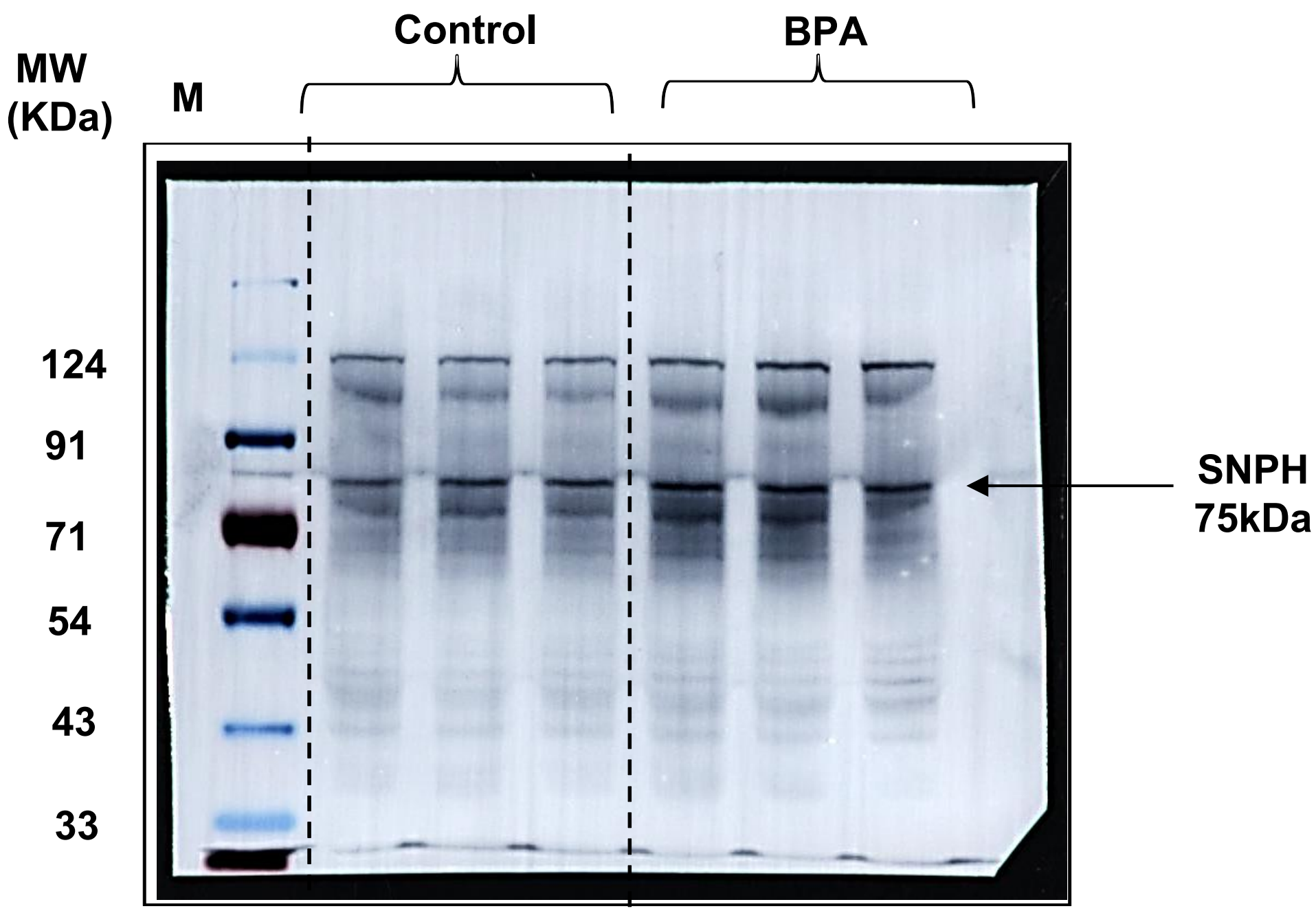

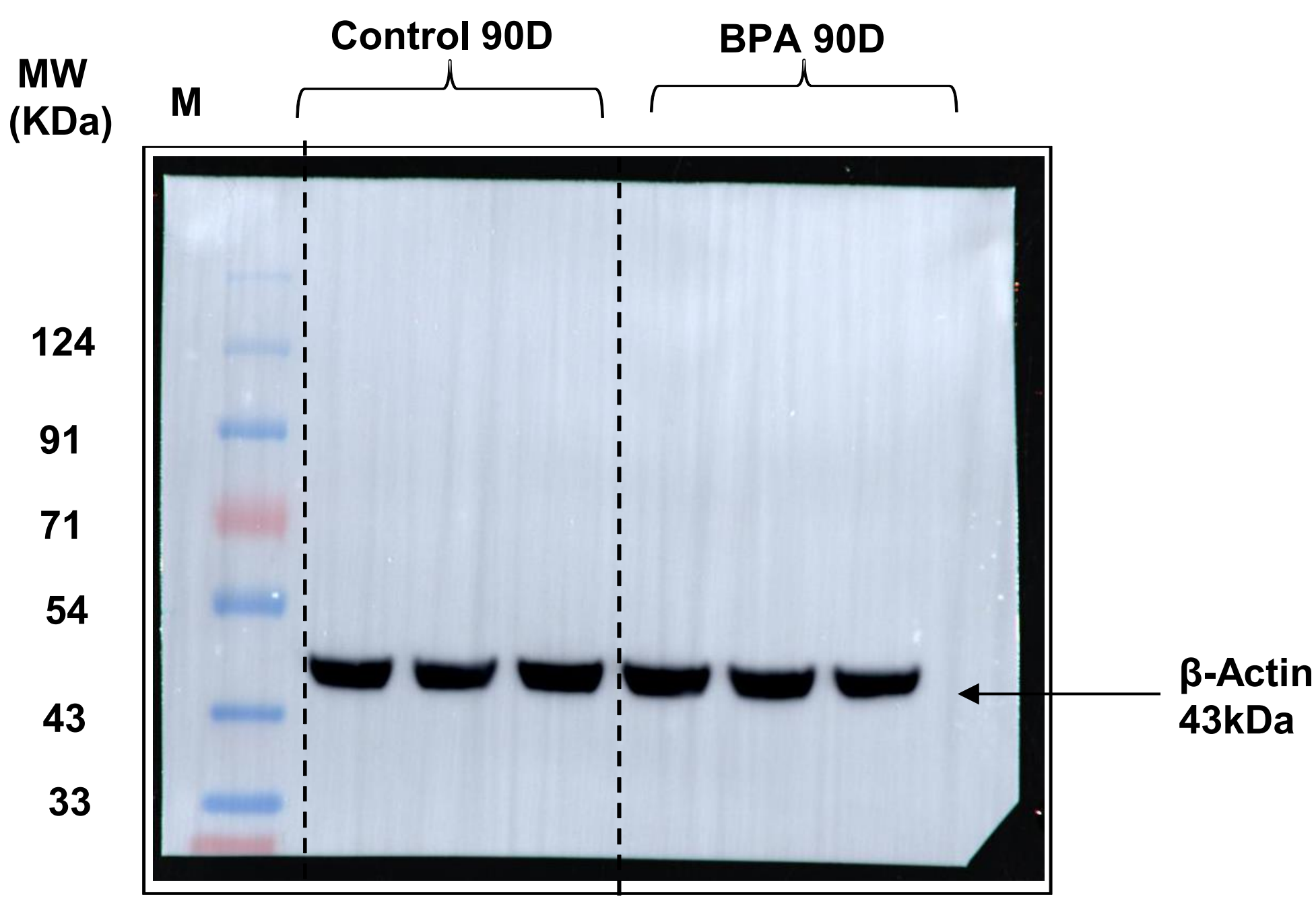

Supplementary Fig. S5

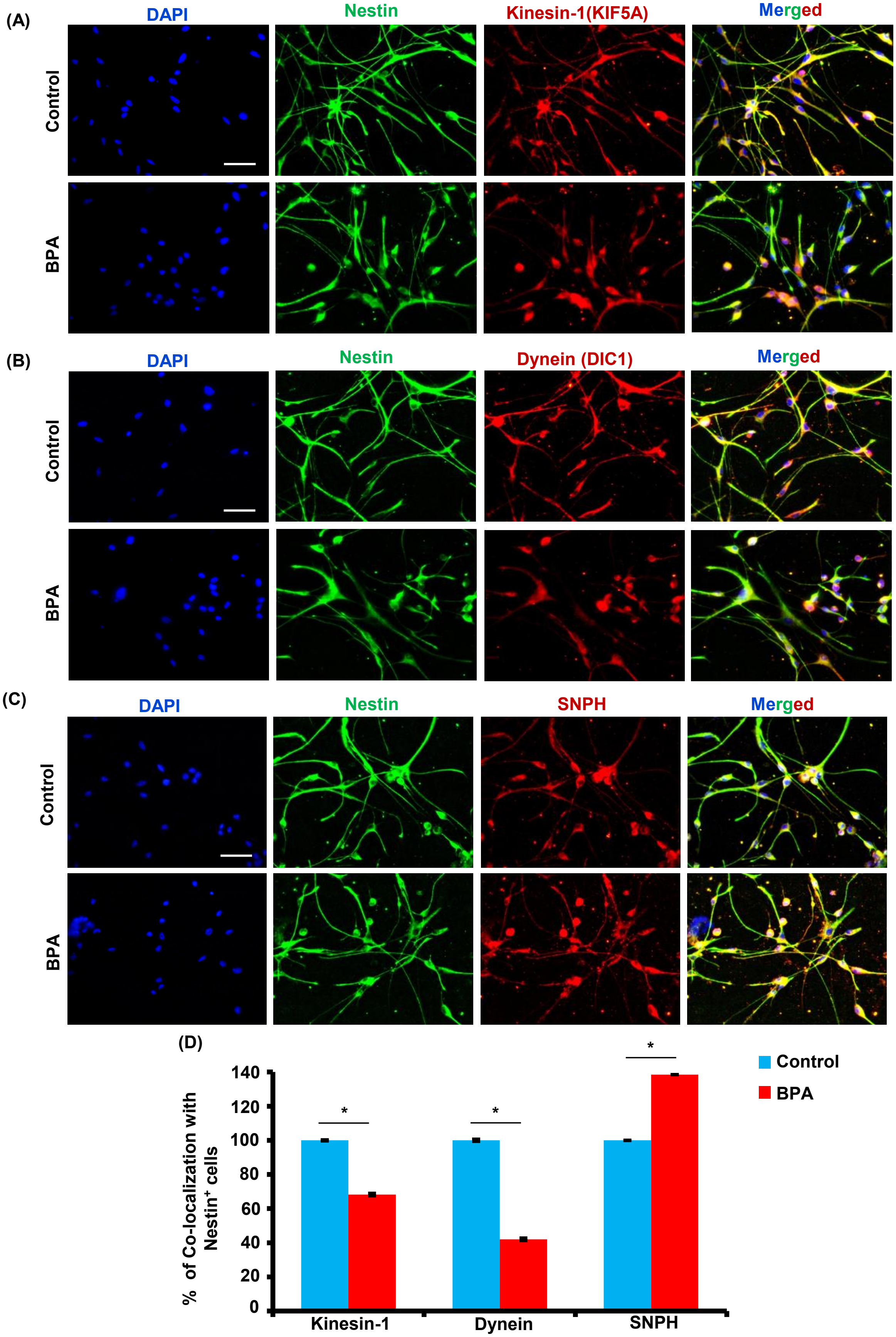

Supplementary Fig S6 (A)

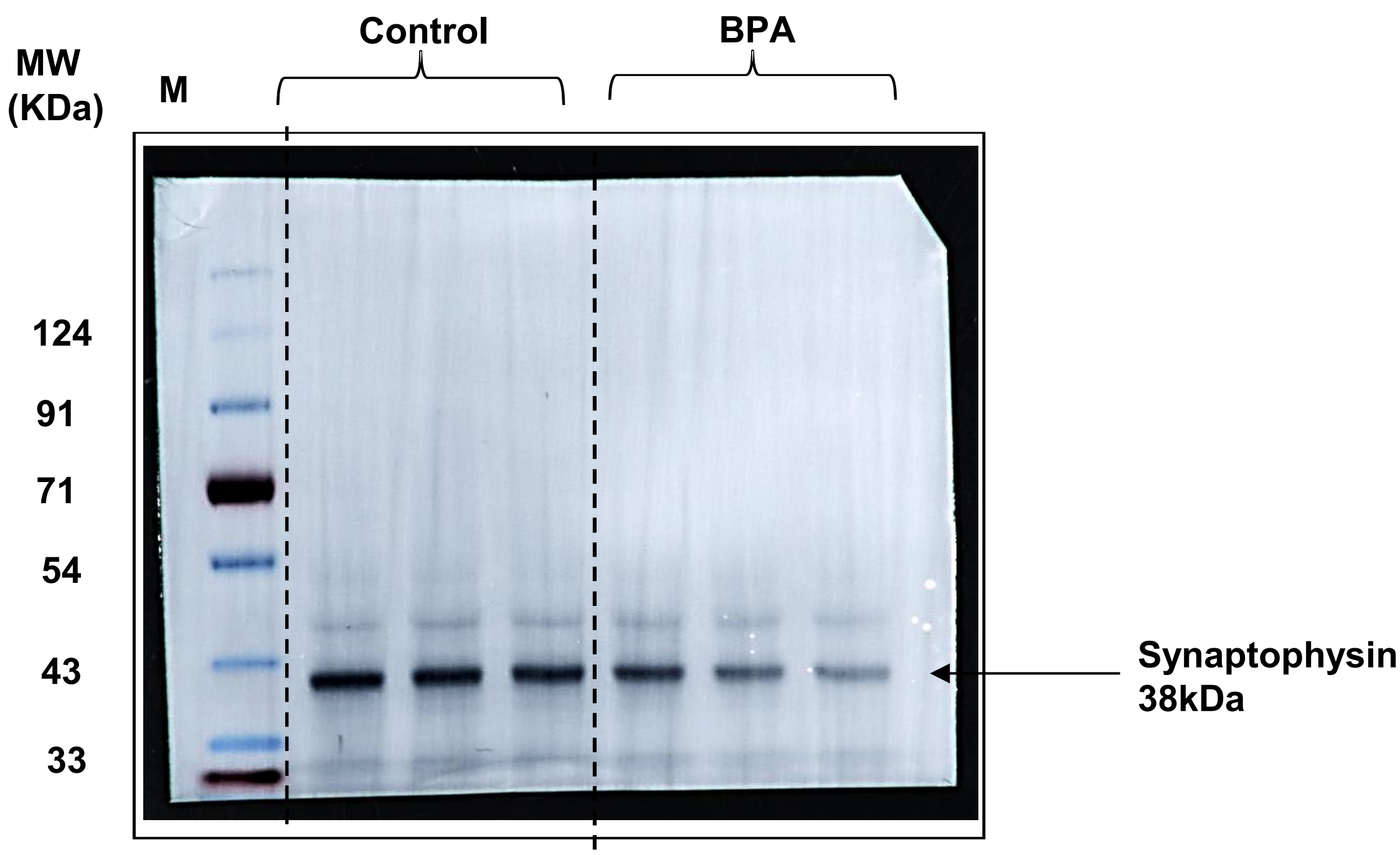

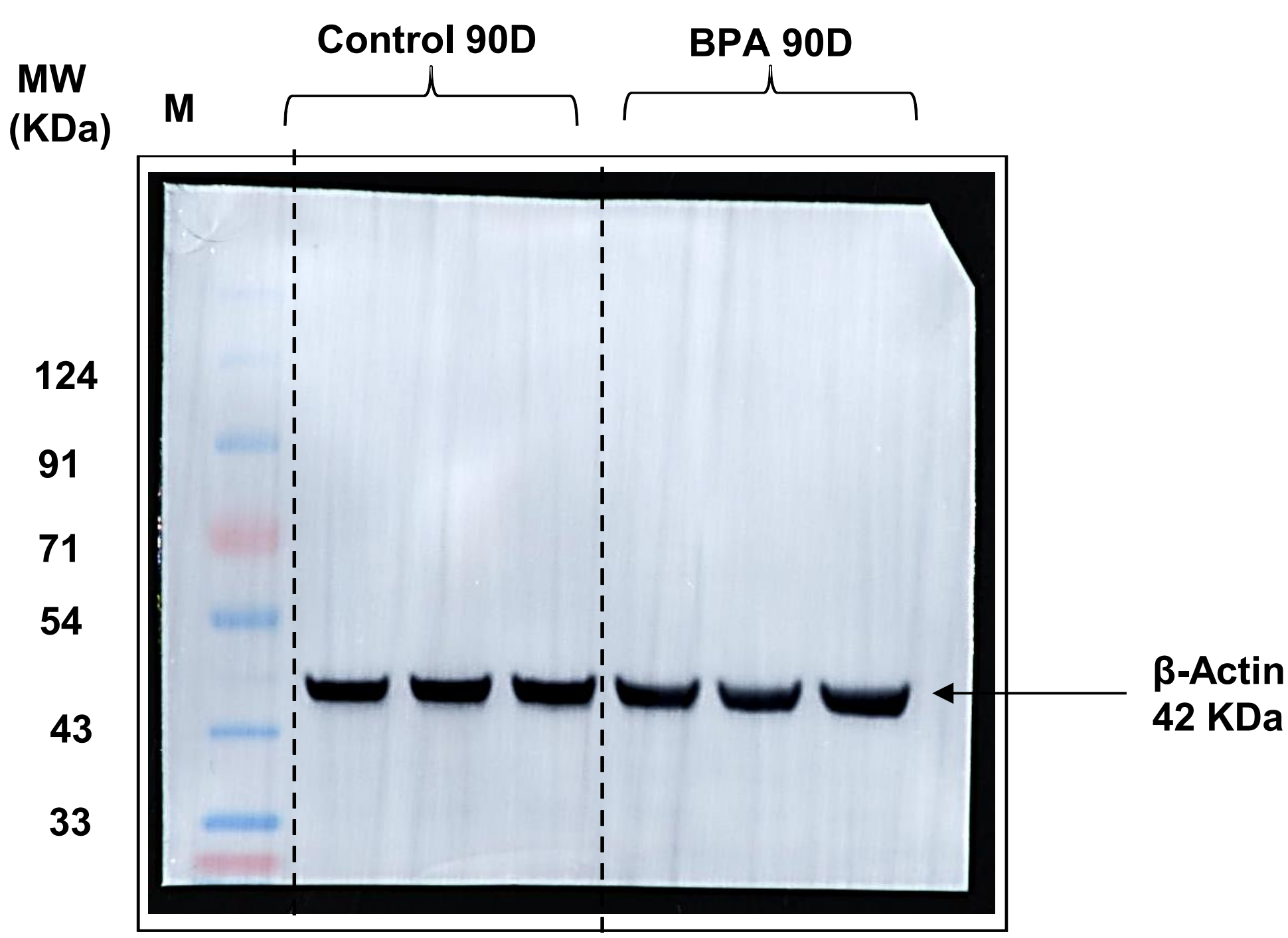

Supplementary Fig S6 (B)

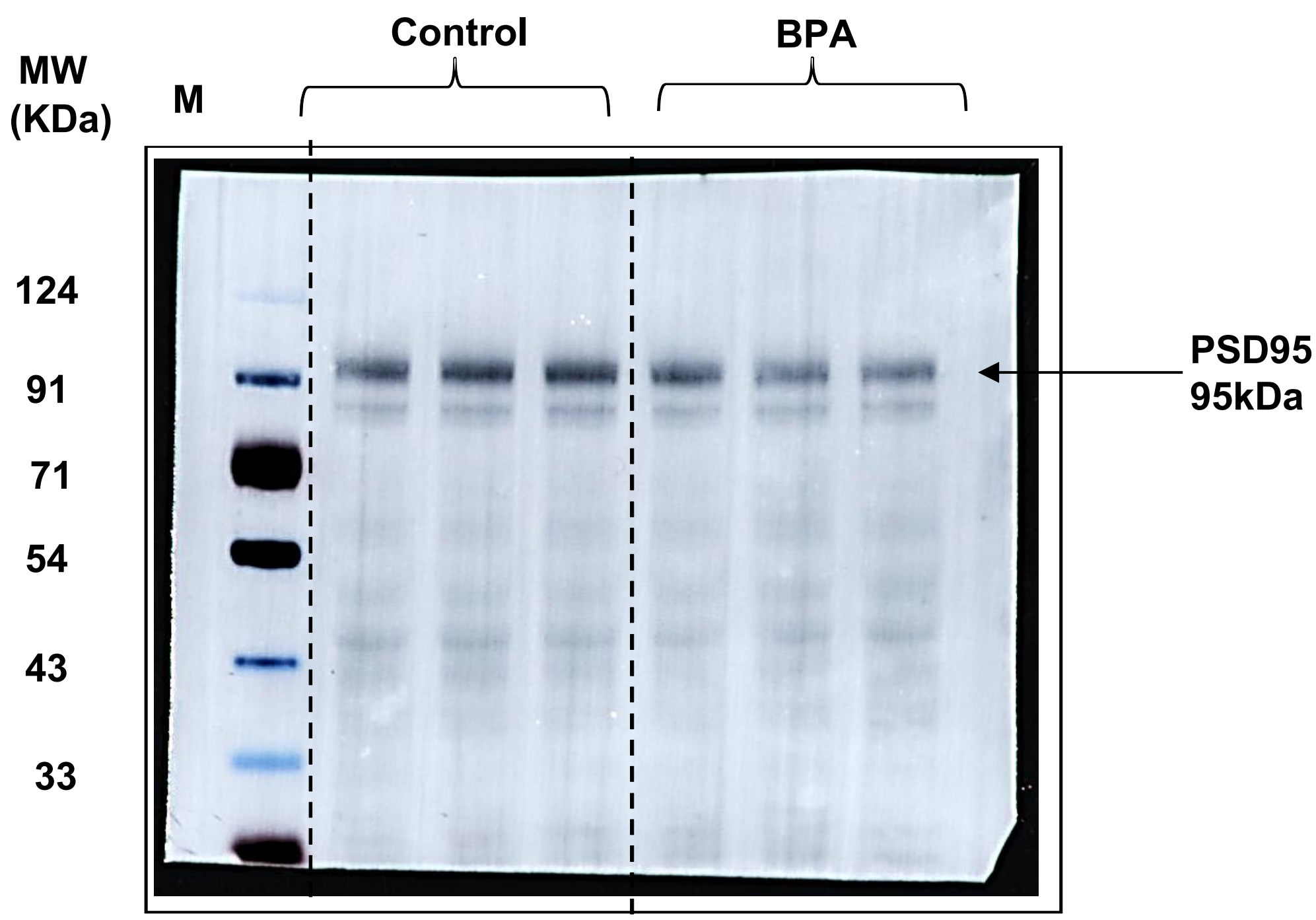

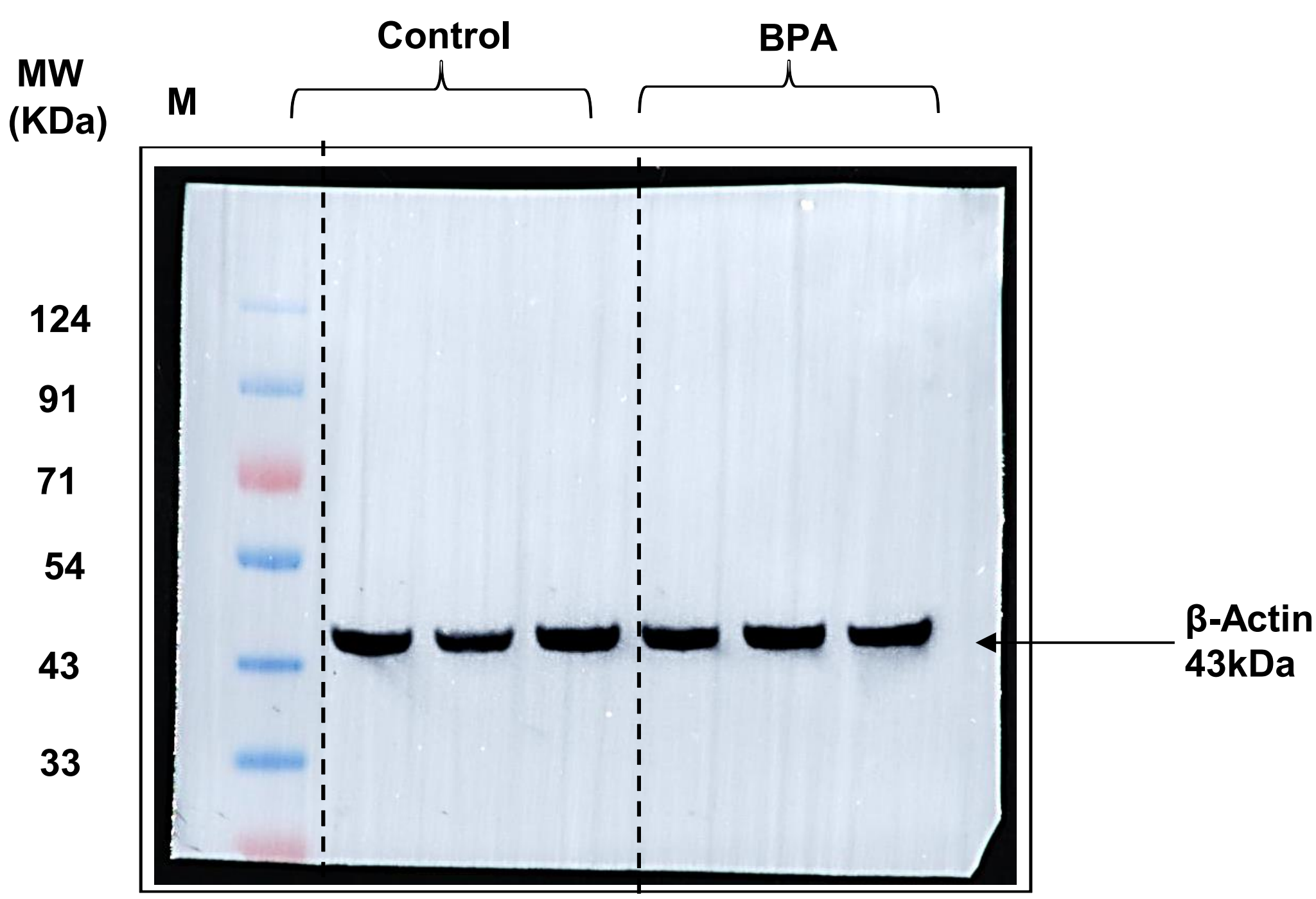

Supplementary Fig S7 (A)

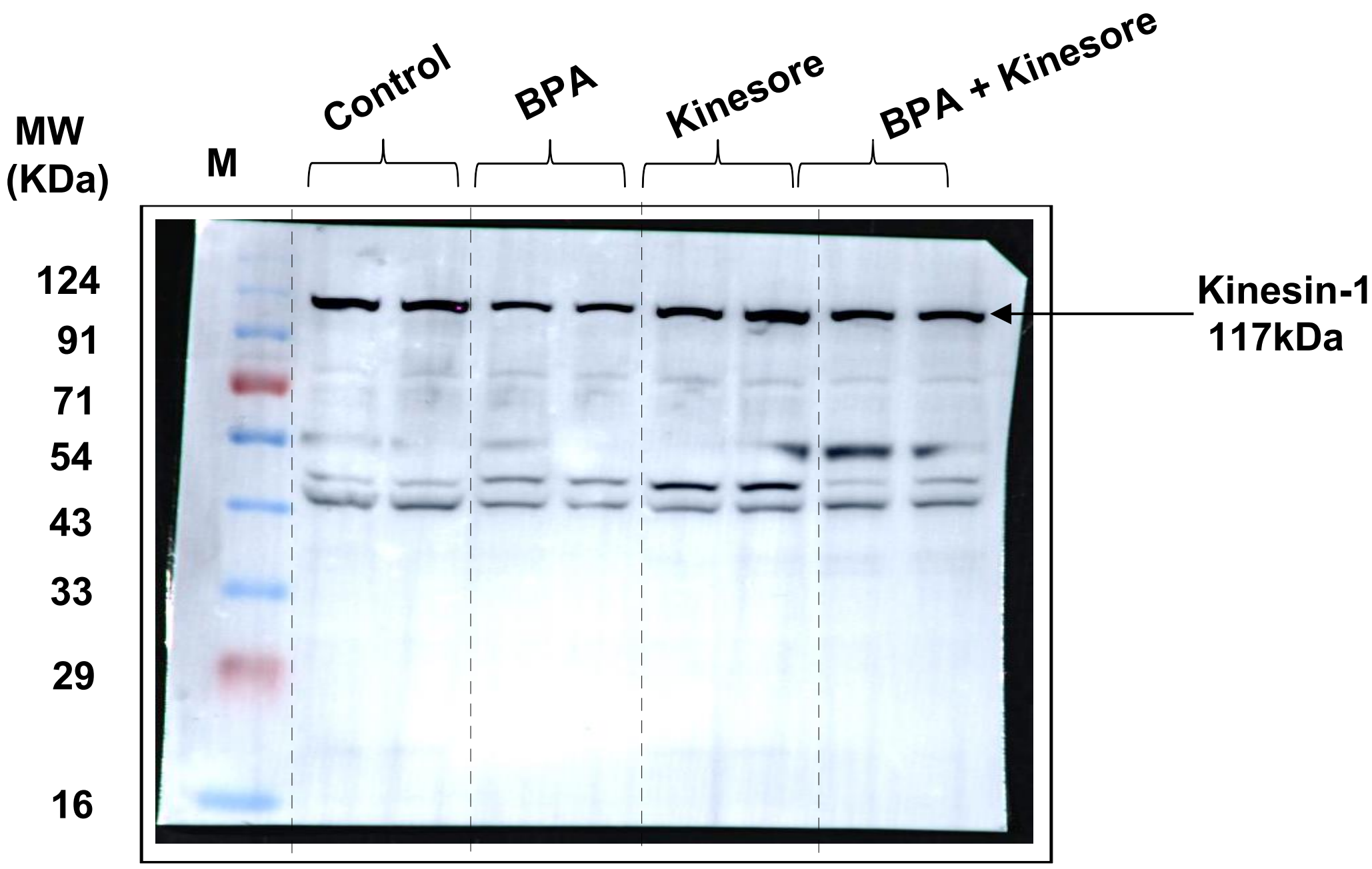

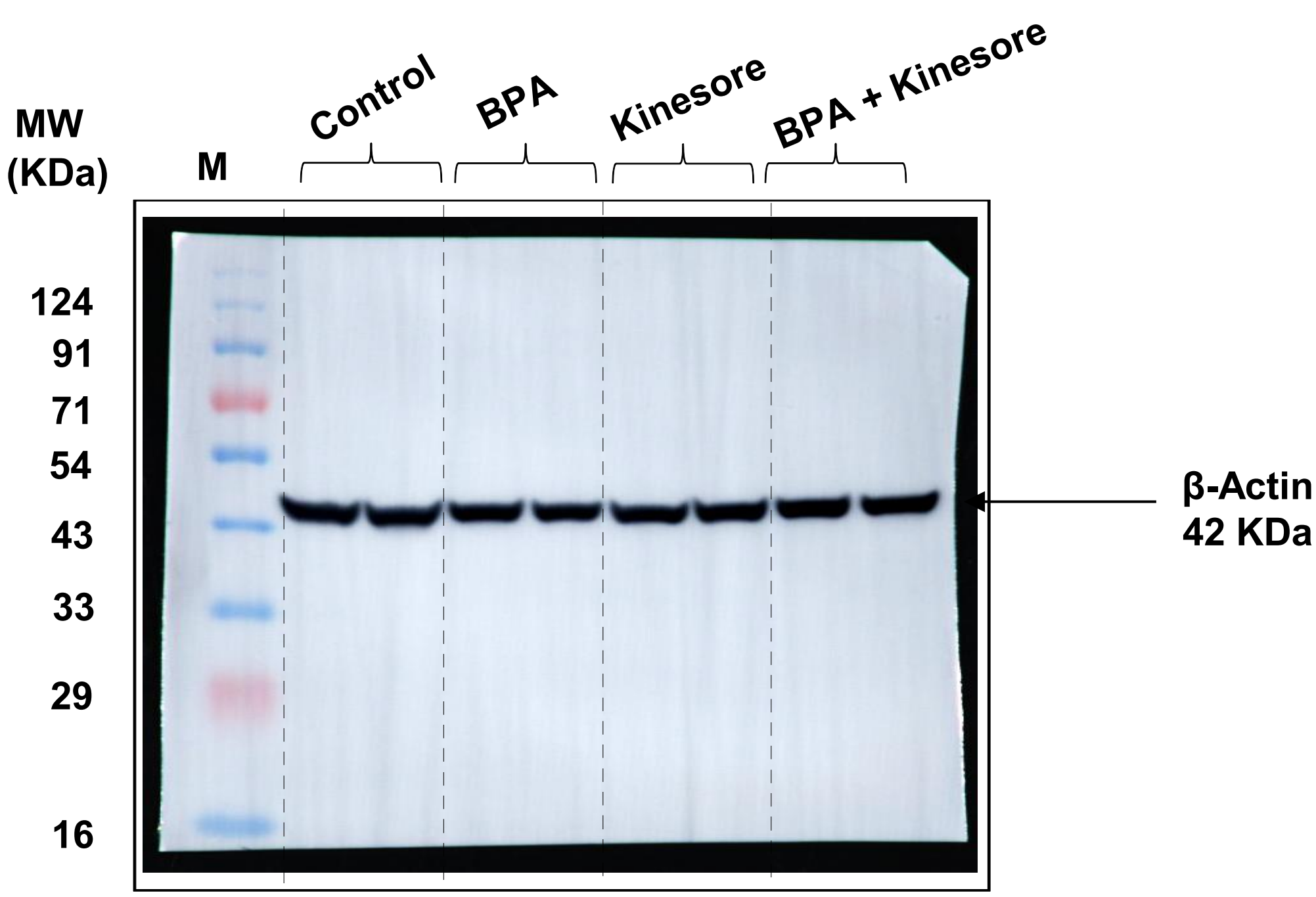

Supplementary Fig S7 (B)

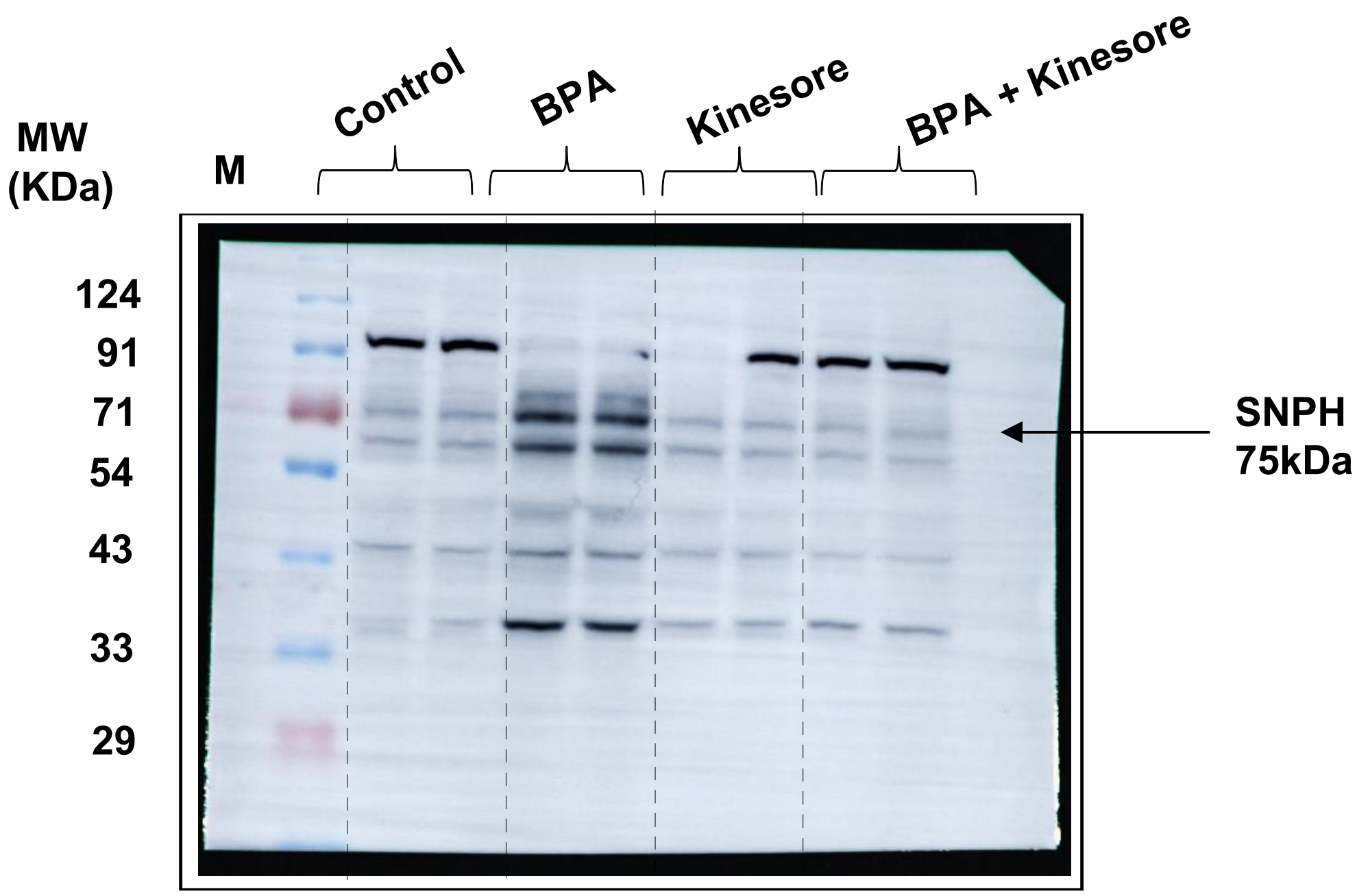

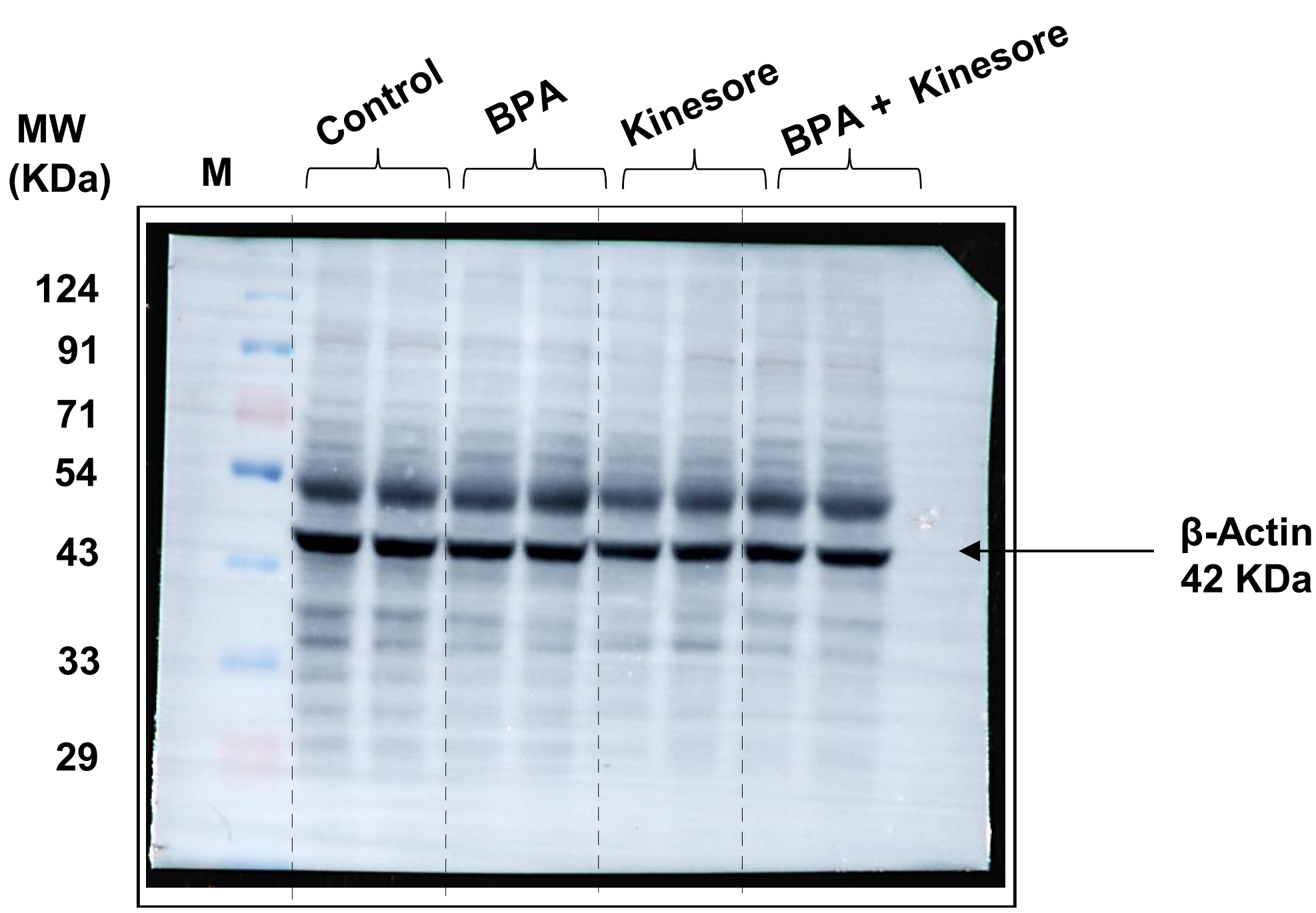

Supplementary Fig S8

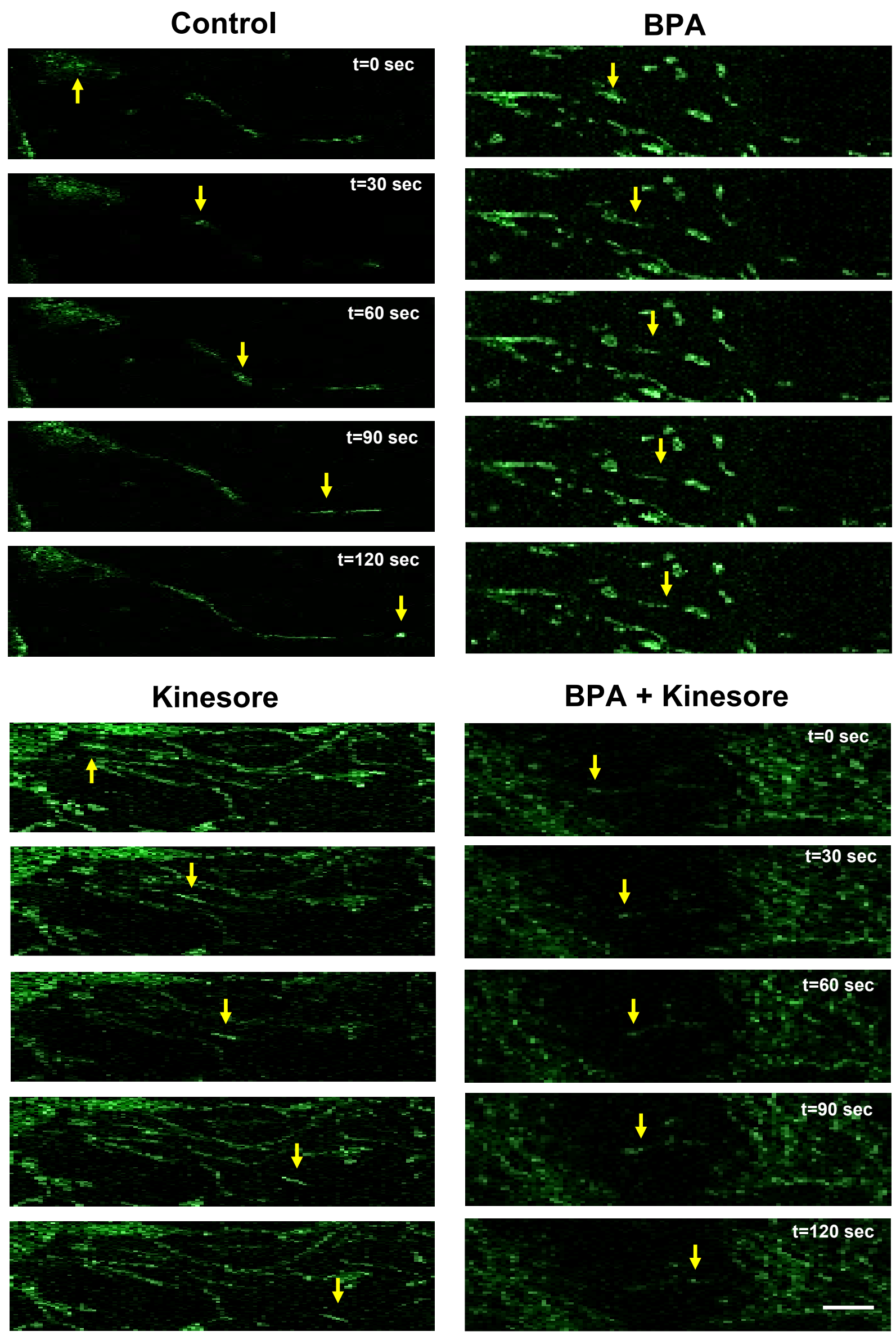

Supplementary Fig S9

(A)

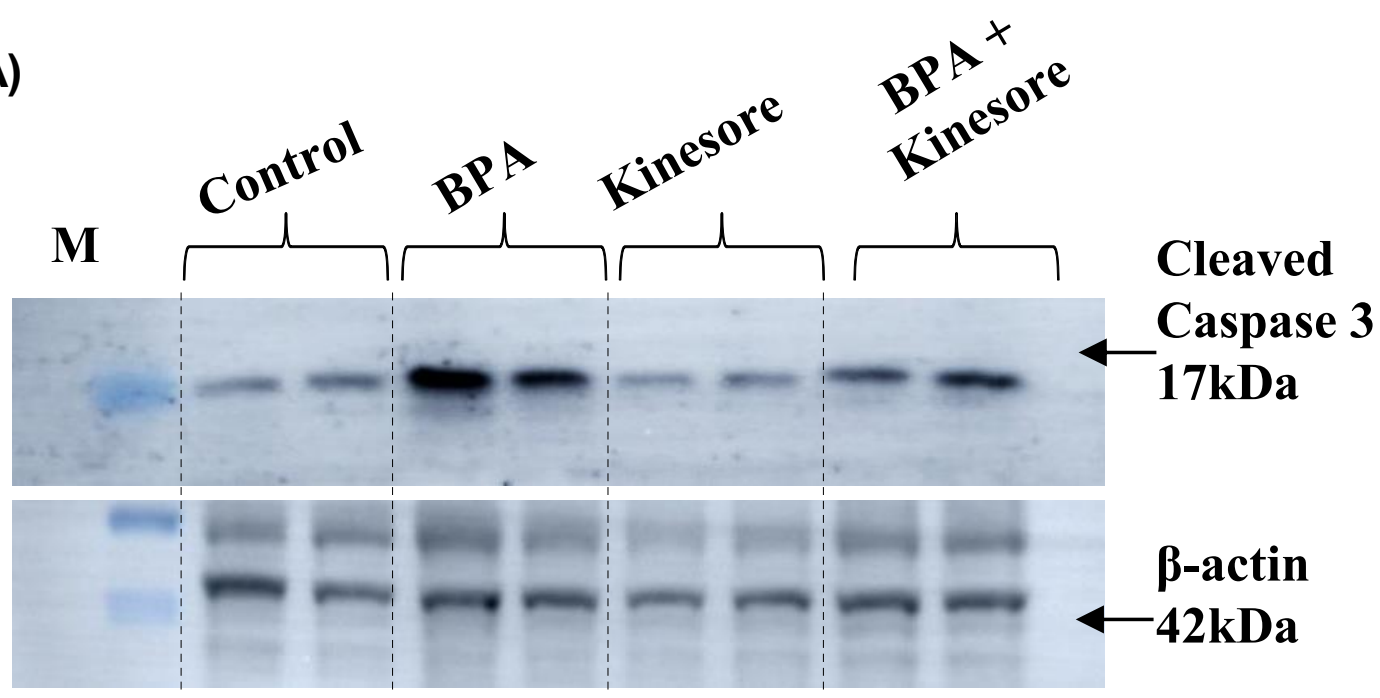

(B)

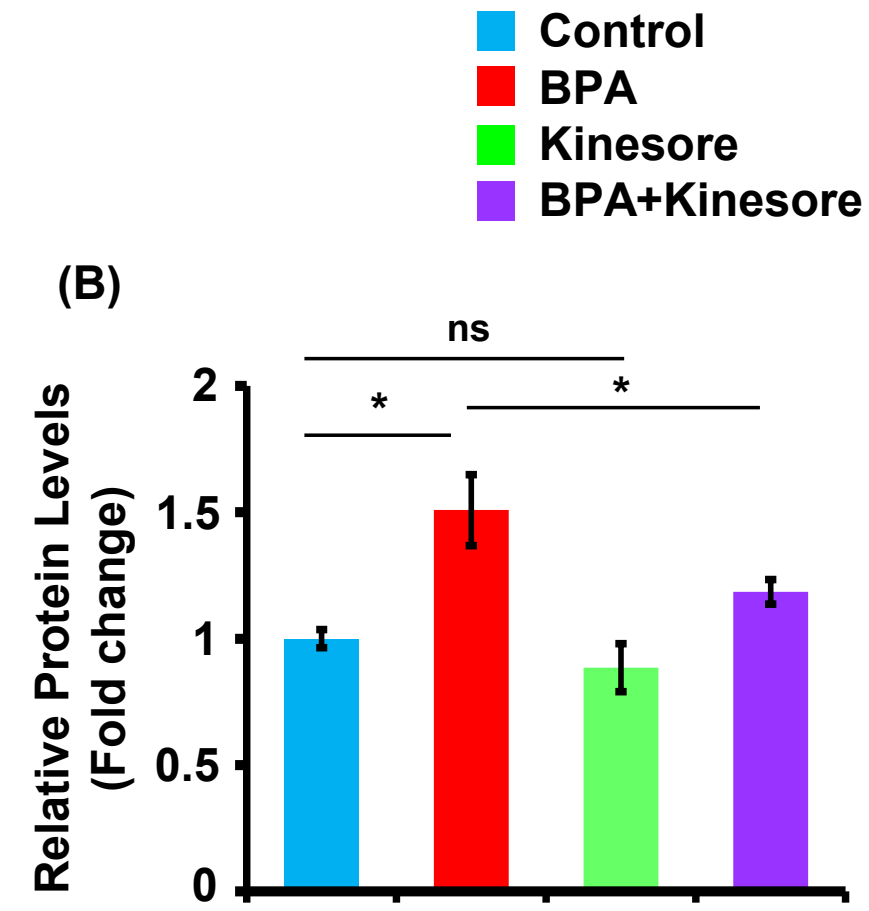

(C)

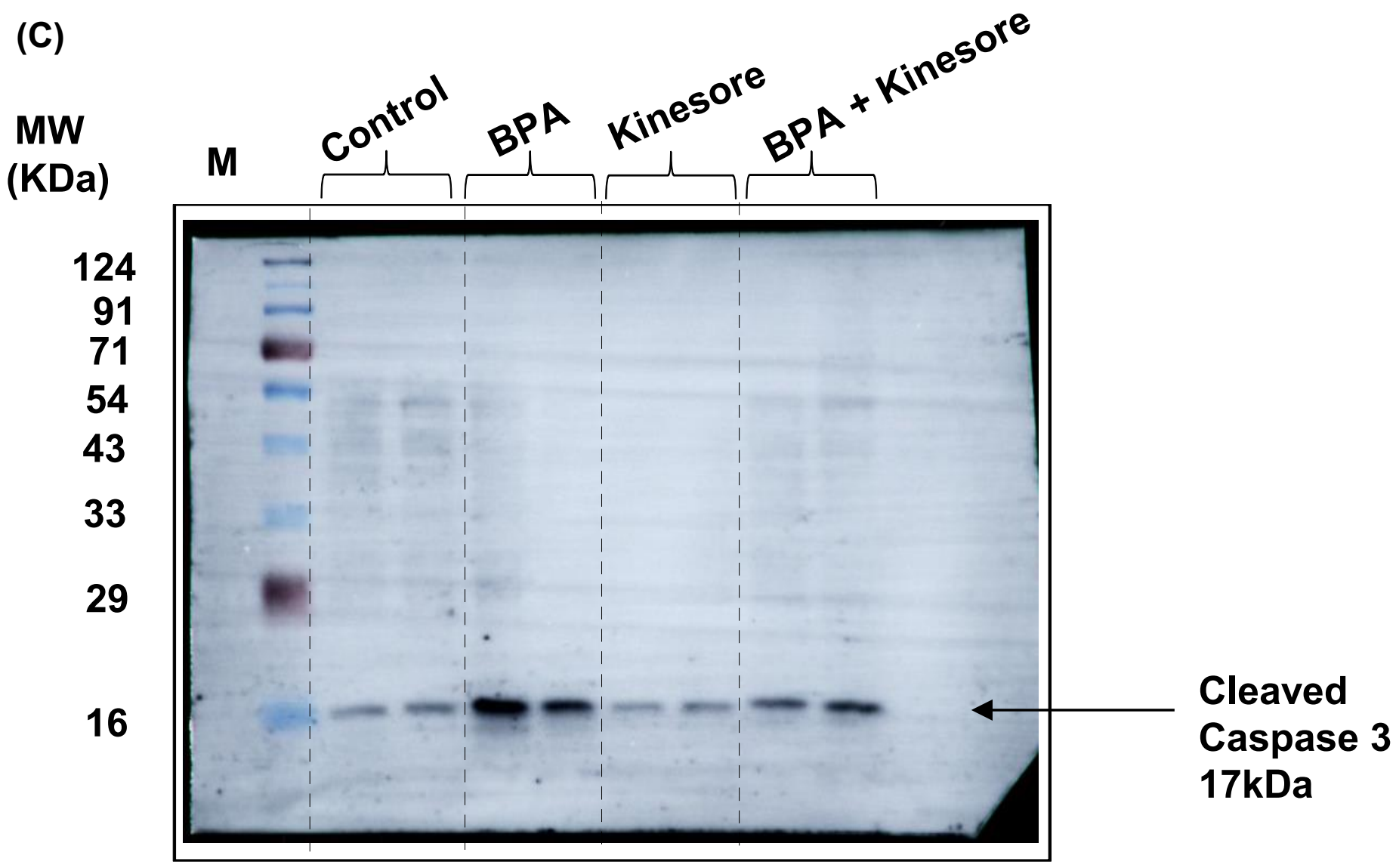

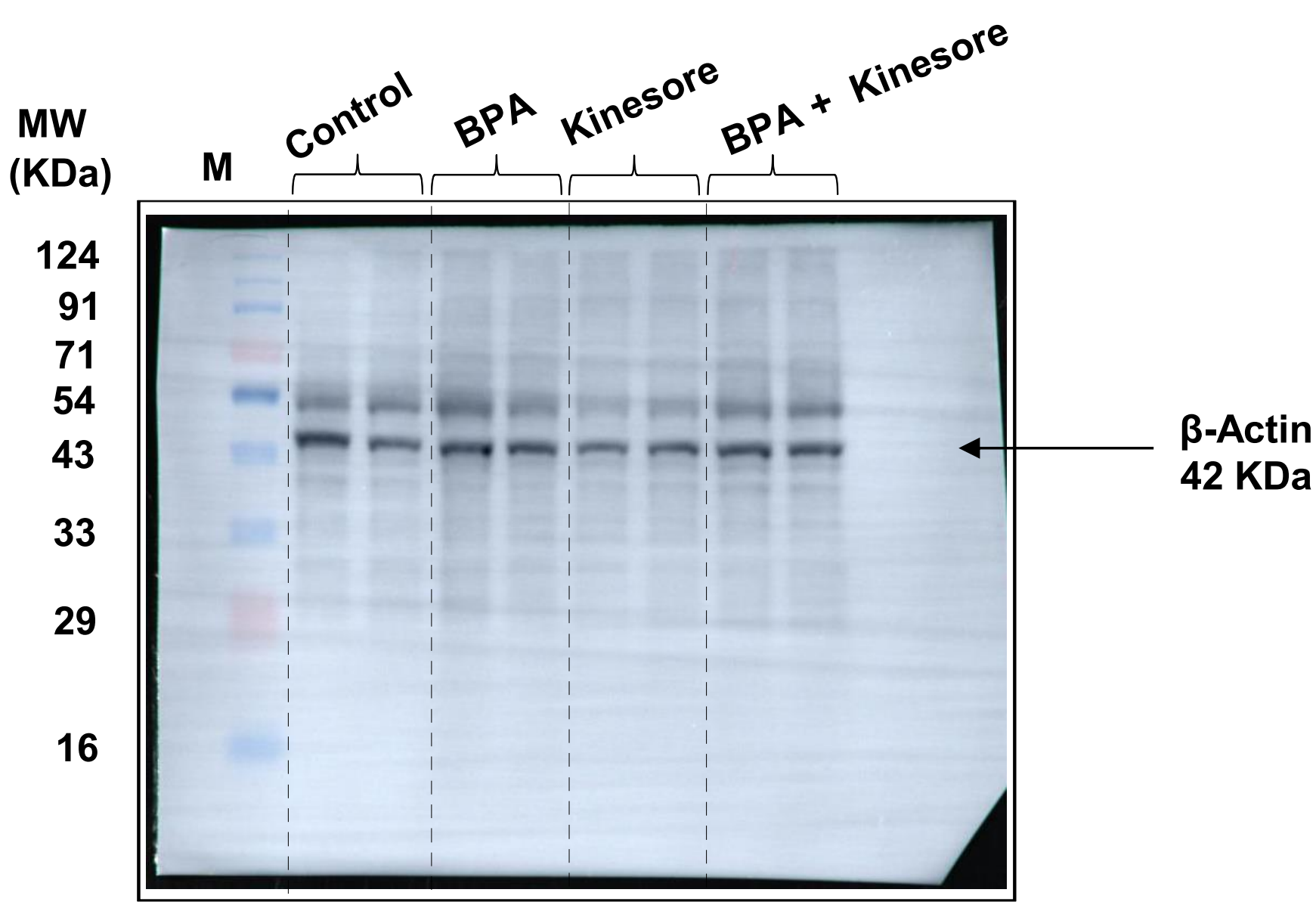

Supplementary Fig S10

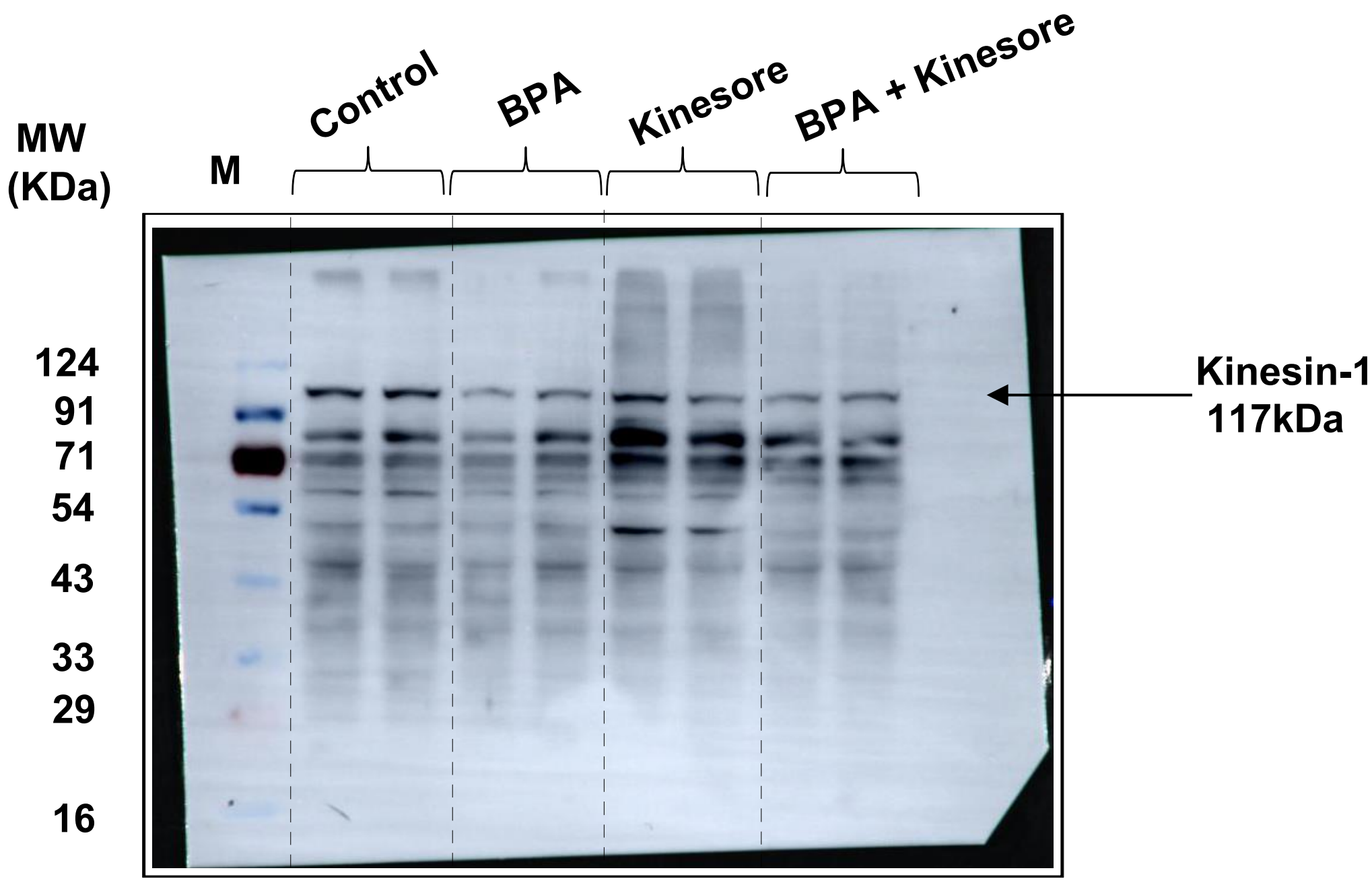

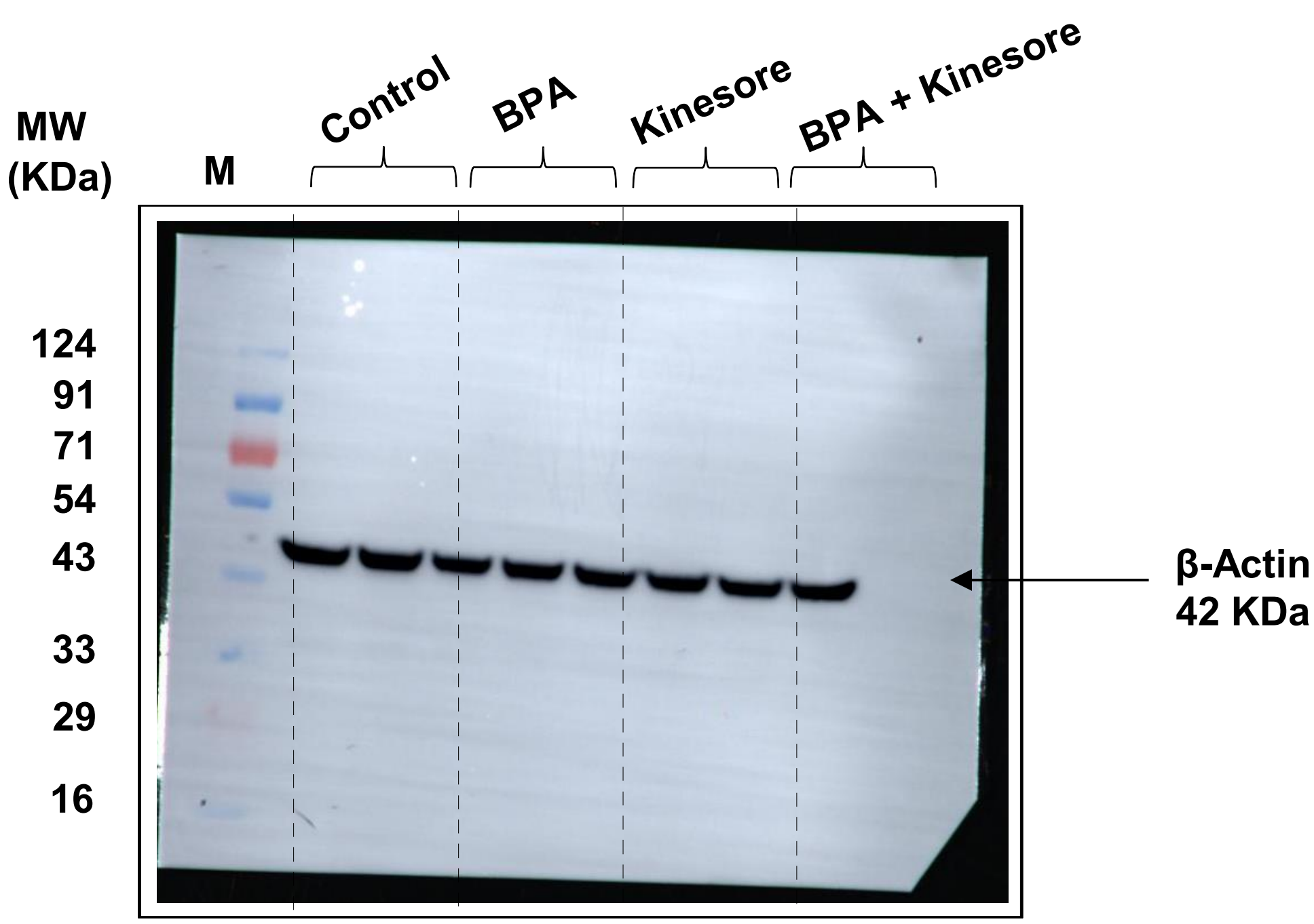

Figure Legends

Fig. S1 (A-D): Effects of BPA (100 µM) exposure on lysosomal (Lyso-Tracker Green, 200 nM) and endosomal (FM4-64, 10 µM) trafficking in hippocampal NSCs-derived neurons assessed by fluorescence time-lapse live-cell imaging. At a concentration that significantly impairs mitochondrial mobility, BPA exposure did not induce significant alterations in lysosomal or endosomal movement as compared to control cultures. The mean±SEM was used to express the values (n=3 individual experiments/group). ns=non-significant, Scale bar=10 µm.

Fig. S2A: *In-silico* prediction of the interaction of BPA with mitochondrial trafficking machinery proteins *via* AutoDock 4.2 software. Molecular docking studies of BPA were done within the catalytic site of Kinesin-1, (KIF5B and KIF5C), and SNPH. The ribbon diagram shows the secondary structure of the proteins consisting of coiled-coil α helices and β sheets strands. Another view is the 2D surface representation of the binding of ligands to the active site residues of the target.

Table S1. The binding energies of docked molecules are also predicted by Discovery Studio 4.0.

Fig. S2B: *In vitro* binding validation of BPA interactions with motor proteins using BPA-derived quantum carbon dots. Quantum carbon dot-based assays suggesting interaction/association with mitochondrial motor proteins.

Fig. S3: Effects of BPA treatment (40 µg/kg body weight, oral) during postnatal day (PND) 21-PND90 on the expression/levels of mitochondrial transport, trafficking alternate motor genes/proteins were studied by quantitative RT-PCR in the hippocampus region of the brain. β-Actin served as a housekeeping gene for normalization. BPA exposure did not cause significant (*p<0.05) changes in the mRNA expression in alternate motor proteins, and microtubule-associated protein 1B (MAP1B) compared to controls. *p<0.05, ns=non-significant

Fig. S4 (A-E): Panels in Figure S4 are uncropped, full-length, and raw versions of blots shown in the main Figure 3. Western blot analysis revealed corresponding changes in protein levels following BPA exposure. BPA significantly reduced the levels of KIF5A and Dynein, and increased SNPH levels. In contrast, no significant changes were observed in the expression of KIF5B and KIF5C. Protein quantification was performed by normalizing band intensities to β-actin. M denotes molecular marker, n = 6 rats/group.

Fig. S5 (A-D): BPA reduced mitochondrial transport during NSCs proliferation in the rat hippocampal-derived NSCs cultures. The representative images of immunofluorescent with the graphs related to quantitative analysis suggested BPA significantly decreased Kinesin-1(KIF5A) and Dynein co-localization with Nestin, while significantly (*p<0.05) increased SNPH/Nestin co-localized cells in the NSCs culture from the rat brain hippocampus. DAPI was used as a nuclear stain. The mean±SEM was used to express the values (n=3 individual experiments/group). *p<0.05. Scale bar=50 µm**.**

Fig. S6 (A-B): Panels in Figure S6 are uncropped, full-length, and raw versions of blots shown in the main Figure 6. Western blot analysis was performed to assess the levels of synaptic function-related proteins (Synaptophysin and PSD95) in the hippocampus of BPA-treated and control rats at postnatal day (PND) 90. BPA exposure significantly reduced the protein levels of

Synaptophysin and PSD95. Protein quantification was conducted by normalizing band intensities to β-actin. M denotes molecular marker, n = 6 rats/group.

Fig. S7 (A-B): Panels in Figure S7 are uncropped, full-length, and raw versions of blots shown in the main Figure 7. The effects of Kinesin-1 (KIF5A) activation by Kinesore on KIF5A and SNPH protein levels were evaluated by Western blot analysis using hippocampal neuronal lysates. Kinesore-mediated activation of KIF5A in BPA-exposed cultures effectively restored the protein expression levels of both KIF5A and SNPH compared to the BPA-only treatment group. β-actin was used as a normalization control for assessing relative protein density. M denotes molecular marker, n=3 individual experiments/group.

Fig. S8: Effects of BPA (100 μM) and Kinesin-1 activator Kinesore (50 μM), for 24h on mitochondrial trafficking in the NSCs-derived neurons. The representative images of fluorescent confocal microscopy representing mitochondrial movement were observed using staining of mitochondria with MitoTracker Green in hippocampal NSCs-derived neuron cultures. BPA-mediated impaired mitochondrial movement was recovered significantly after Kinesin-1(KIF5A) activation by Kinesore. Arrows show movement mitochondria in axon of neurons. Scale bar=10 μm**.**

Fig. S9 (A-C). BPA-induced caspase-3 activation was significantly attenuated by activation of KIF5A by Kinesore in NSCs-derived neuronal cultures. The quantitative relative protein density was normalized with β-actin. The data are represented as mean ± SEM (n=3 individual experiments/group). *$p<0.05$, ns=non-significant. Marker is represented as M.

Fig. S10: Panels in Figure S10 are uncropped, full-length, and raw versions of blots shown in the main Figure 9. Immunoblot analysis of KIF5A protein expression in the rat hippocampus following BPA and Kinesore treatment in the rat hippocampus. BPA exposure resulted in a significant reduction in KIF5A protein levels compared to control rats. Kinesore treatment alone significantly increased KIF5A expression, while co-administration of Kinesore in BPA-exposed rats effectively restored KIF5A protein levels relative to BPA-treatment alone. Relative protein levels were quantified by normalizing to β-actin. M denotes molecular marker, n=6 rats/group
